# A Comprehensive Atlas of Immunological Differences Between Humans, Mice, and Non-Human Primates

**DOI:** 10.3389/fimmu.2022.867015

**Published:** 2022-03-11

**Authors:** Zachary B. Bjornson-Hooper, Gabriela K. Fragiadakis, Matthew H. Spitzer, Han Chen, Deepthi Madhireddy, Kevin Hu, Kelly Lundsten, David R. McIlwain, Garry P. Nolan

**Affiliations:** ^1^ Department of Microbiology and Immunology, Stanford University, Stanford, CA, United States; ^2^ Department of Medicine, Division of Rheumatology, University of California San Francisco, San Francisco, CA, United States; ^3^ Bakar ImmunoX Initiative, University of California San Francisco, San Francisco, CA, United States; ^4^ University of California, San Francisco (UCSF) Data Science CoLab and University of California, San Francisco (UCSF) Department of Medicine, University of California, San Francisco, San Francisco, CA, United States; ^5^ Immunology Program, Stanford University, Stanford, CA, United States; ^6^ Departments of Otolaryngology – Head and Neck Surgery and Microbiology and Immunology, University of California, San Francisco, San Francisco, CA, United States; ^7^ Parker Institute for Cancer Immunotherapy, San Francisco, CA, United States; ^8^ Chan Zuckerberg Biohub, San Francisco, CA, United States; ^9^ BioLegend Inc, Advanced Cytometry, San Diego, CA, United States

**Keywords:** CyTOF mass cytometry, rhesus macaque (Macaca mulatta), cynomolgus monkey (Macaca fascicularis), African green monkey (AGM) (Chlorocebus aethiops), mouse, immune cell signaling

## Abstract

Animal models are an integral part of the drug development and evaluation process. However, they are unsurprisingly imperfect reflections of humans, and the extent and nature of many immunological differences are unknown. With the rise of targeted and biological therapeutics, it is increasingly important that we understand the molecular differences in the immunological behavior of humans and model organisms. However, very few antibodies are raised against non-human primate antigens, and databases of cross-reactivity between species are incomplete. Thus, we screened 332 antibodies in five immune cell populations in blood from humans and four non-human primate species generating a comprehensive cross-reactivity catalog that includes cell type-specificity. We used this catalog to create large mass cytometry universal cross-species phenotyping and signaling panels for humans, along with three of the model organisms most similar to humans: rhesus and cynomolgus macaques and African green monkeys; and one of the mammalian models most widely used in drug development: C57BL/6 mice. As a proof-of-principle, we measured immune cell signaling responses across all five species to an array of 15 stimuli using mass cytometry. We found numerous instances of different cellular phenotypes and immune signaling events occurring within and between species, and detailed three examples (double-positive T cell frequency and signaling; granulocyte response to *Bacillus anthracis* antigen; and B cell subsets). We also explore the correlation of herpes simian B virus serostatus on the immune profile. Antibody panels and the full dataset generated are available online as a resource to enable future studies comparing immune responses across species during the evaluation of therapeutics.

## Introduction

Animal models are core to drug development and evaluation: all candidate therapeutics are evaluated for toxicity, pharmacokinetics/pharmacodynamics (PK/PD), and efficacy using animals. Furthermore, in circumstances when human trials of efficacy cannot be conducted for ethical reasons or when an insufficient number of natural cases exists, such as for therapeutics for biothreat agents, the U.S. Food and Drug Administration may allow licensure after efficacy is evaluated only in animal models under 21 CFR 314 (“The Animal Rule”). This rule has been applied in a limited number of cases since its issuance in 2002, including Raxibacumab for inhalational anthrax, B12 for cyanide poisoning, pyridostigmine for nerve gas, and levofloxacin and moxifloxacin for bubonic plague (1), but therapeutics for an increasing number of conditions are being evaluated *via* this mode.

Unsurprisingly, animal models are not perfect surrogates for humans (see (2), among other reviews). From a wide view, most diseases affect only a limited number of species. Modeling these diseases is inherently challenging, and researchers use a variety of techniques to compensate for these imperfections, such as the use of mouse-adapted viruses (3) and immunocompromised or gene mutated mice (4) for viral and cancer models, respectively, that do not accurately recapitulate disease progression in humans.

Furthermore, even when a model appears reflective of a disease at a wide view, e.g. by physical examination, differences in finer details such as functions of cellular receptors or kinases between species may adversely affect the evaluation of therapeutics. For example, while human CD16 (type III FCγ receptor) interacts with IgG1 and IgG3, macaque CD16 instead interacts with IgG1 and IgG2 (5); additionally, CD16 is absent from macaque granulocytes (5, 6). These differences would likely confound the evaluation of therapeutics antibodies that act through this FCγ receptor (7). On a systems level, there is almost no correlation of transcriptomic responses to burn, trauma, and endotoxemia between humans and mice (8), underscoring that these species have evolved unique mechanisms to heal and combat disease. While expression patterns of some orthologous genes may be generally similar between species, many genes have divergent expression patterns; regulatory elements especially have a lower level of conservation across species, as do lineage-specific responses (9, 10). The increasing prevalence of biotherapeutics designed to target these receptors and pathways has emphasized the importance of understanding the molecular and cellular differences in immune signaling between animal models and humans.

Differences in protein expressions between species can also have profound effects on safety determination. For example, Fialuridine (TGN1412, CD28 superagonist antibody) was initially evaluated in four animal models (mice, rats, dogs, and monkeys), but when administered to humans at just 1/500^th^ of the dose found to be safe in animals, it caused catastrophic organic failure due to differences in CD28 expression between species (11). It is also important to consider differences between human demographic groups used in clinical trials in terms of ages, sex, ethnicities, immunological histories, genetics, etc. These differences are perhaps better recognized than those between animal models and humans: the US Food and Drug Administration since 1998 has required that new drug applications describe safety and effectiveness by sex, age, and race, and the labels for 38% of drugs approved from 2004 to 2007 include PK/PD data by ethnicity (12). Warfarin, rosuvastatin, tacrolimus, carbamazepine, and others are known to have ethnicity-dependent PK/PD (12). A variety of factors, including hormones, body fat, and blood flow, contribute to sex differences in bioavailability, distribution, metabolism, and excretion of drugs (13). These issues highlight the necessity of understanding immunological variability both within and between species.

Non-human primates (NHPs) are critical components of drug development because of their similarity to humans. Many key immunology assays make use of antibodies to demarcate specific cell types and quantify signaling moieties. Very few antibodies are raised against non-human primate antigens; instead, researchers typically use anti-human antibodies that are cross-reactive with the non-human primate species that they are studying. To help researchers find antibodies for NHP research, the National Institutes of Health supports a highly valuable database of the cross-reactivity of commercially available antibodies with 13 NHP species (http://www.nhpreagents.org). The database is derived from manufacturer and investigator reports, and typically provides a simple yes/no statement about whether a clone stains a species, with occasional comments about staining intensity or specificity. While an invaluable resource, the database is limited in its coverage. For example, prior to this study, only 28 CD markers had been evaluated in African green monkeys.

Additionally, with few exceptions, the database lacks information about the cell types bound by cross-reactive antibodies, and there are many known instances of antibody clones binding different cell types in different species. For example, granulocyte and monocyte marker expression is known to be substantially different in humans than in non-human primates. Anti-human CD33 clone AC104.3E3 was reported in the NIH database and manufacturer’s datasheet as cross-reactive with rhesus and cynomolgus macaques, but our lab and others determined that in those species, it prominently stains granulocytes (14, 15), while in humans it stains monocytes and classical dendritic cells. As another example, the Fcγ receptor CD16 is found on granulocytes in humans and sooty mangabeys, but not in macaques or baboons (5, 6), which will likely confound animal studies evaluating therapeutic antibodies, which may bind, transduce signals through and mediate internalization *via* this Fcγ receptor. Yet another example is CD56, which is expressed on monocytes in macaques (16), but is a canonical NK cell marker in humans. Thus, researchers must confirm that each clone they use is staining the cell population of interest through literature review or experimental verification.

To expand both the breadth and depth of primate cross-reactivity data. We screened 332 monoclonal antibodies in blood from humans and four NHP species: rhesus macaque (*Macaca mulatta*), cynomolgus macaque (*Macaca fascicularis*), African green monkey (*Chlorocebus aethiops*) and olive/yellow baboon (*Papio hamadryas anubis* x *Papio hamadryas cynocephalus* hybrid); and found more than 120 clones that stained one or more populations in each species. Furthermore, we included counter-stain antibodies that allowed us to determine staining specificity in at least five major immune cell populations. This dataset is available online at https://flowrepository.org (accession FR-FCM-Z2Z7).

We used the results from this screen to create the first universal cross-species mass cytometry phenotyping and signaling panels for humans and a set of frequently used animal models. As proof-of-principle for the ability of these panels to detect similarities and differences between species during the evaluation of therapeutics, we performed phospho-flow immune signaling profiling of whole blood from 86 healthy humans, 32 rhesus macaques (*Macaca mulatta*), 32 cynomolgus macaques (*Macaca fascicularis*), 24 African green monkeys (*Chlorocebus aethiops*) and 50 C57BL/6 mice (*Mus musculus*), measuring 16 signaling proteins and 24 surface markers per cell in 12 immune cell populations after treatment with a panel of 15 stimuli, using mass cytometry. The set of phenotyping panels was designed to demarcate orthologous populations in all species, while the signaling panel and complementary stimuli were targeted at innate immunity and cytokine responses. We present an overview of this cross-species analysis here, including several examples of specific differences between species. The complementary manuscript by Fragiadakis et al. (17) analyzes the human dataset in detail, including an examination of demographic differences, presenting a reference for human immune profiling studies. The entirety of the immunophenotyping and signaling dataset is available online at https://flowrepository.org (accession FR-FCM-Z2ZY).

## Results

### Establishing an Antibody Reactivity Screen

**Figure 1:**
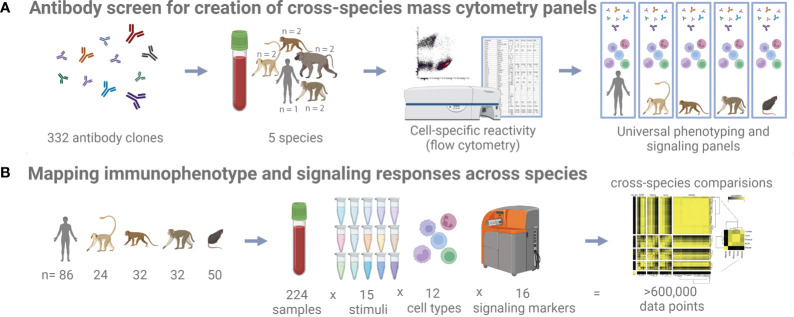
Study schematic. **(A)** 332 antibody clones were tested on five different species by flow cytometry to identify cross-reactive clones. **(B)** Whole blood samples from five different species and subjected to 15 different stimuli were profiled with a universal mass cytometry antibody panel capable of identifying 12 different cell types.

Towards the goal of creating universal cross-species mass cytometry panels, we first conducted a comprehensive flow cytometry antibody cross-reactivity screen to identify suitable clones (**Figure 1A**). To determine immune cell-type specific reactivity for a set of monoclonal antibodies across species, we designed a flow cytometry panel of counterstain antibodies that delineates major circulating immune cell types in human, cynomolgus macaque, rhesus macaque, African green monkey, and baboon blood (**Figure S1A**). We strategically selected the fluorophores for the counterstain antibodies to keep the PE channel, which was used for the screened antibody, free of bleed/compensation to avoid technical artifacts. This panel readily identified granulocytes, B cells, T cells, NK cells, and monocytes/dendritic cells. In all species, except for African green monkey, we could additionally separate the monocytes and dendritic cells based on CD11b expression. In African green monkeys, CD11b (ICRF44) was non-reactive, and we chose not to include a substitute marker to maintain technical consistency across all species. After testing many protocols aimed to preserve antigen staining and effectively lysing non-human primate blood (data not shown), fixation and red blood cell lysis conditions were selected. Fixation with 0.26% paraformaldehyde (PFA) was low enough to avoid obvious loss of staining and sufficient to minimize morphological changes of cells as observed as forward and side scatter signals changing over the course of acquisition on the cytometer. Enzymatic lysis with VersaLyse was highly effective for both human and NHP blood, whereas other methods such as hypotonic lysis were inadequate or inconsistent for NHP blood.

Using these processing conditions, we conducted a flow cytometry screen on fresh whole blood samples from humans and NHPs (total n=9, consisting of n=2 biological replicates per NHP species and n=1 human control), separately testing 332 antibody clones. An antibody clone was initially classified as reactive with a population if more than 10 percent of cells had a PE signal intensity greater than the 95th percentile of the intensity of the corresponding isotype control for the same species and population (**Table S1**). This threshold was found to accurately reflect the results of manual inspection: We verified 500 of the 14,940 clone x species x population results, taking into consideration reported staining patterns [references included (16, 18–24)] and the visual degree of separation from the isotype control, and calculated a false-positive rate of 7.4% and a false-negative rate of 1.6%, for an initial accuracy of 91% in our data. Then, we manually verified and corrected as necessary all discordant replicates and all clones that were classified as reactive in a non-human primate species but not in a human; thus, our final estimated accuracy exceeds 91%. In total, we identified 259, 148, 125, 159, and 147 clones that are reactive with one or more populations in human, cynomolgus macaque, rhesus macaque, African green monkey, and baboon, respectively (**Table S1**).

All data from this screen are publicly available for researchers to independently validate the reactivity of clones across species (https://flowrepository.org/id/FR-FCM-Z2Z7).

### Notable Examples in Differences in Expression Patterns Between Species Revealed by Screen

In macaques CD33 is found on granulocytes and CD16 is restricted to monocytes and dendritic cells, unlike in humans (5, 6, 14, 15). In this study, we found that African green monkeys share this same staining pattern to macaques, albeit with weaker CD33 staining. Another notable difference (out of numerous idiosyncratic expression patterns observed) is that CD172g (signal regulatory protein (SIRP) γ, also known as SIRPβ2) is expressed on CD11b+ monocytes and granulocytes, but not on T cells, in all of the NHP species examined. In contrast, this marker in humans is expressed on T cells, some B cells, and to some degree in granulocytes (**Figure S1B**). Because it lacks a cytoplasmic signaling domain, CD172g is postulated to signal unidirectionally by activating the CD47-expressing cell and inducing T cell migration and proliferation in humans, mice, and rats (25–27). Thus, our finding suggests a major difference in regulation of immune cell migration and adaptive response, which warrants further study.

Another notable example of the differences found is the presence of CD2 staining not only on T cells, but also on B cells of rhesus macaques, cynomolgus macaques, African green monkeys and to a slight extent baboons (**Figure S1C**). CD2 is involved in adhesion, co-stimulation, antigen recognition, and potentially differentiation (28, 29). In mice, virtually all circulating and bone marrow B cells express CD2 (28). Kingma et al. previously reported that a small subset (3.69 +/− 1.6%) of normal human peripheral blood B cells express CD2 (30), although we observed no CD2+ B cells in the human control, likely due to donor- or clone-specific differences between studies. B cell CD2 expression thus seems to have declined evolutionarily, with the most abundant expression in the oldest species (mouse), moderate expression in macaques and African greens, less expression in baboons, and essentially no expression in the youngest species (human). Interestingly, the ligand of CD2 is not conserved between species: CD2 exclusively binds CD48 in mice and rats, while in humans it strongly binds CD58 and only weakly binds CD48, which is also co-expressed with CD58 (29, 31).

### CyTOF Panel Design

We used the results of the antibody screen, along with the results of several targeted follow-up experiments, to craft a set of parallel CyTOF mass cytometry cell phenotyping panels for rhesus and cynomolgus macaque, African green monkey, and human (**Table S2**). Wherever possible, we used the same antibody clones for all species. In the case of erythrocytes, we used the common marker CD235a for humans and CD233 for NHPs, instead of using the less common CD233 for all species (no anti-CD235 clone reactive with NHPs could be found). In the case of CD11c, the Bu15 clone used for humans was non-reactive in NHPs and therefore clone 3.9 was used in place. However, clone 3.9 is reported to preferentially bind activated CD11c and requires the addition of magnesium as a cofactor during staining (32, 33). Therefore, we primarily use CD16 in lieu of CD11c during gating analysis for consistency. In the case of naïve and memory T cells, we observed a very broad distribution of CD45RA staining, as previously reported (34, 35), with two clones (5H9 and HI100), which was difficult to gate (see online dataset). Staining of this marker was superior with the VersaLyse-based method than with the Triton-X100-based method (see *Materials and Methods*) (data not shown).

It was generally possible to use the same clones for African green monkeys as for macaques, with several exceptions (**Table S2**). No reactive clones were found for CD11c; instead, we again rely on CD16 for gating monocyte subpopulations and CD123, CD1c, and BDCA3 for gating dendritic cell subpopulations. Additionally, using clones well established in previous studies, we created a similar panel for mice that delineates the same populations as those targeted in the primate panel, enabling parallel gating of all five species (**Table S2**).

A signaling antibody panel was designed to deliver cell signaling readouts for cell types identified by cross-species phenotyping panels. Signaling epitopes are highly conserved and, with only one exception, the same antibody clones were usable in all five species (**Table S2**).

Many steps were taken to reduce error and technical variability (see *Materials and Methods*). These CyTOF panels were exhaustively titrated to determine optimal staining concentrations for each antibody, then revalidated in at least two donors from each species. Antibodies were conjugated in bulk, and we subsequently contracted BioLyph LLC to lyophilize and package several thousand single-use pellets (“LyoSpheres”) of each of these panels for stability and to eliminate pipetting errors from repeatedly assembling cocktails.

### Phenotyping, Stimulation and Signaling Assays

To map similarities and differences in immunophenotype and signaling responses across species, we assayed fresh whole blood from 86 healthy humans, 32 rhesus macaques, 32 cynomolgus macaques, 24 African green monkeys, and 50 C57BL/6 mice after *ex vivo* stimulation with 15 separate treatments (**Figure 1B** and **Table S5** for list of stimuli). The stimuli and readouts were selected for their clinical relevance and their roles in disease and innate immunity (see *Materials and Methods*). Many of the stimuli are recombinant cytokines. These recombinant proteins were preferably species-matched; i.e. recombinant rhesus cytokines were used in rhesus macaque blood. Where no suitable proteins were found, we used the closest species available (e.g. macaque cytokines were frequently used in African green monkey blood). Because of the lack of standard assays and reference reagents for non-human primate cytokines that would have enabled using the same number of functional units between species, we instead used the same masses of reagents at concentrations exceeding the EC50 values, thereby minimizing the effect of variations in the cytokine potencies/activities on the assays. Again, to minimize technical variability and error, stimuli were pre-dispensed into single-use plates when possible. Fresh, whole blood was used instead of PBMCs to avoid perturbations and inconsistency from density gradient isolations and storage. We used mass-tag barcoding to combine staining of all stimulation conditions into one tube per donor, eliminating differences in staining volume and processing. Complete automation was used for stimulation, fixation, lysis, barcoding, and staining. CyTOF quality control tests were run before every sample, and internal normalization standards were included to control for variability during runs. Finally, all analysis code has been annotated in Wolfram Language Notebooks and is available upon request, along with the detailed records from antibody conjugations and sample processing.

### Cell Type Frequencies Vary Between Species and Recapitulate the Evolutionary Tree

**Figure 2:**
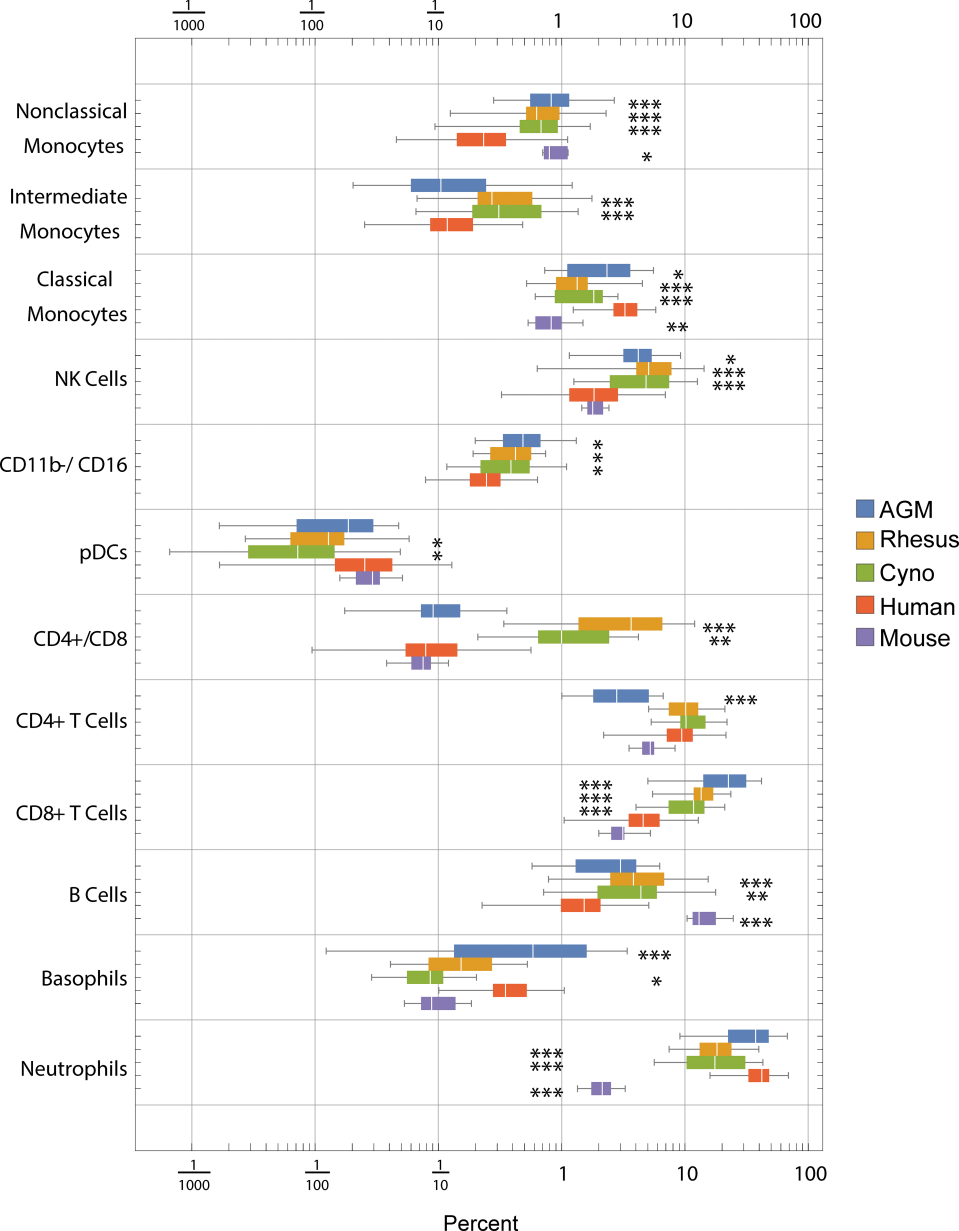
Frequencies (percent of total) of gated cell types by species. Center line: median; Box: 25th to 75th quantile; Whiskers: 1.5x interquartile range. Statistics: ANOVA with Bonferroni post-test was calculated for each population. Asterisks indicate species that are significantly different from humans *p < 5x10^-2^, **p < 1x10^-2^, ***p < 1x10^-3^ after Bonferroni correction.

Using the hierarchy shown in **Figure S2**, we gated blood cells from all species in parallel to determine the distributions of frequencies of these cell types (**Figure 2**). Note that these hierarchies only show the major populations and make use of a subset of our phenotyping panel. It is possible to further subset most of these populations—for example, NK cells can be further subset based on CD20, CCR7 and CD56—and it is our hope that interested researchers will explore the data on https://flowrepository.org (accession FR-FCM-Z2ZY). The frequencies of many cell populations were significantly different between species; notable differences include (a) macaques have more CD4+/CD8+ double-positive T cells than humans, mice or AGMs, (b) mice have approximately 10 times fewer neutrophils than all primates, (c) all non-human primates have approximately three times more B cells than humans, and mice have approximately 10 times more than humans, and (d) humans have a higher ratio of classical to non-classical monocytes than any other species (**Figure 2**).

**Figure 3:**
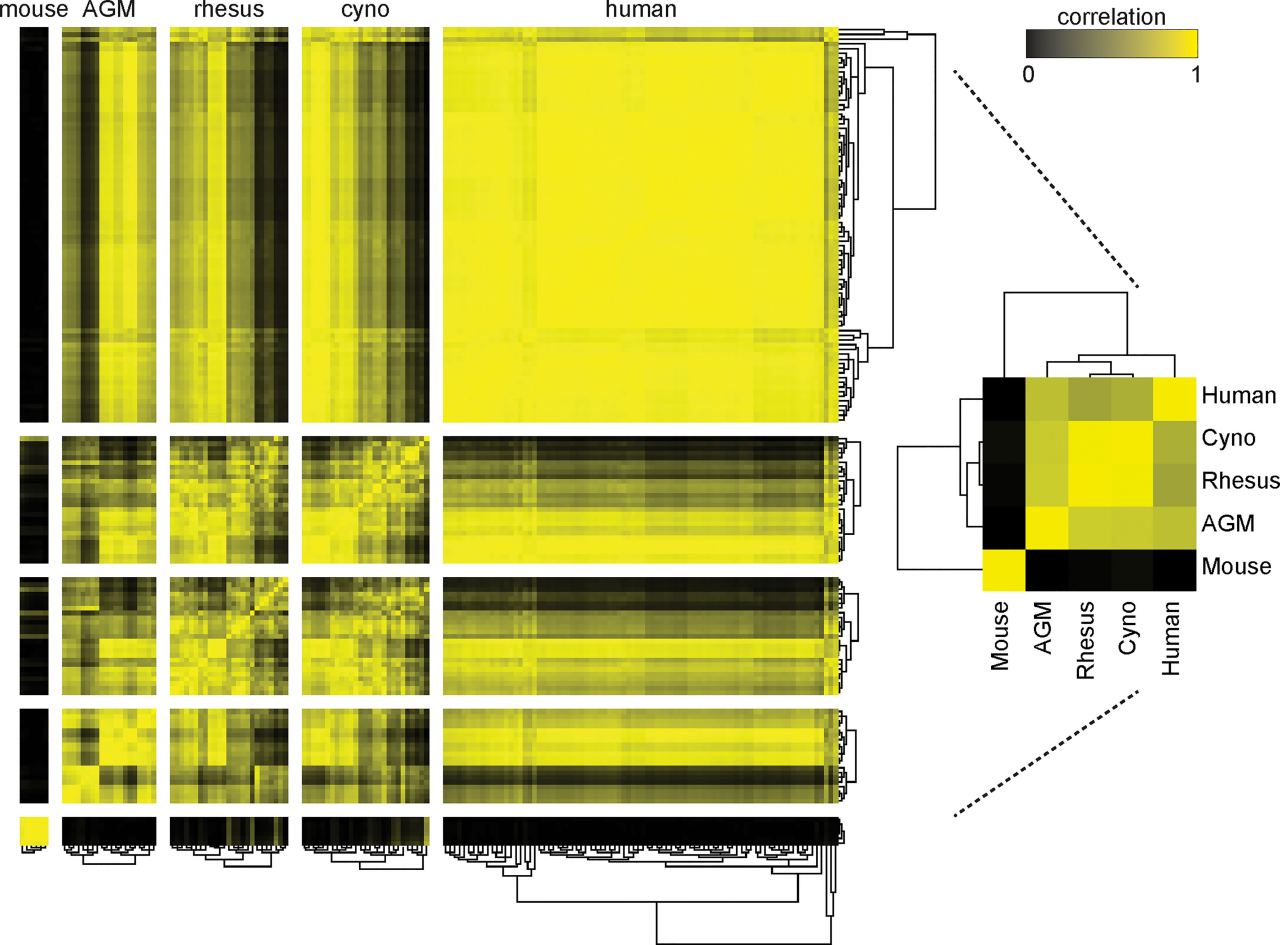
Clustering of species by their cell type frequencies recapitulates the evolutionary tree. The average frequencies of 10 cell types (Neutrophils, Basophils, B Cells, CD8+ T Cells, CD4+ T Cells, CD4+/CD8+ T Cells, pDCs, NK Cells, Classical Monocytes and Nonclassical Monocytes) in each species was calculated and then clustered according to their pairwise correlations between species (right). This major clustering order was preserved in the larger heatmap (left), in which each individual donor is displayed and clustered by their individual cell type frequencies. Metric: PearsonCorrelation[freqs_species_1, freqs_species_2]^2^; distance function: Euclidean; linkage: average.

When we clustered the species by the correlation between their average cell type frequencies, we recapitulated the evolutionary tree (**Figure 3**, right panel): rhesus and cynomolgus macaques diverged most recently, 1.5 to 3.5 million years ago (Ma); macaques and African green monkeys diverged 11.5 to 14 Ma; Old World monkeys and humans diverged 20-38 Ma and mice diverged more than 90 Ma (36–38). This result raises an intriguing possibility for future analysis of a large number of species to look for critical junctures in the evolution of the immune system.

While maintaining this major clustering order, we then sub-clustered individual donors within their species, again by their cell type frequencies (**Figure 3**) (Note that due to limited volume, blood from 50 mice was pooled into six tubes, separated by sex). The intra-species distances between clusters were the smallest for mice, followed by the non-human primates, followed by humans. This is unsurprising considering that the mice were a single inbred strain, the non-human primates were either wild-caught from isolated colonies or captive-bred, and the humans were specifically recruited to include a variety of ethnicities. For the most part, all individuals within a species had similar correlations to other species; nonetheless, some non-human primates were more similar to humans than others. Importantly, clustering did not correlate with the batches in which individuals were processed; that is, batch effects are not a driver of this clustering (data not shown). Full tables of population frequencies for each donor are provided in **Tables S6**, **S7**.

### Orthologous Cell Types in Different Species Have Unique Phenotypes

**Figure 4:**
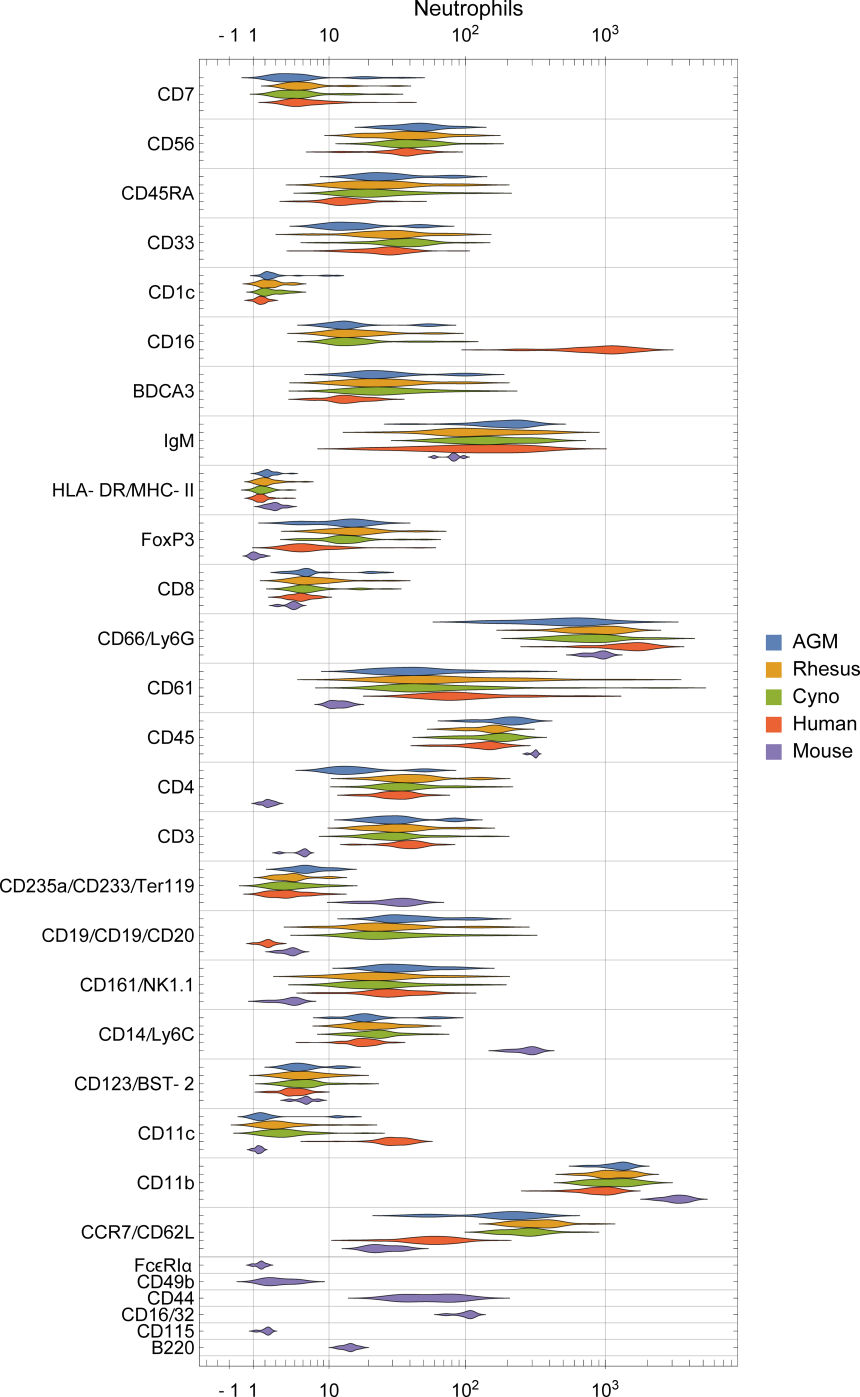
Distribution of surface marker expression for each species in neutrophils. (See **Figure S3** for other populations). Different markers are grouped together if they were on the same channel and stain similar cell types between species (e.g. CD235a/CD233/Ter119 are all on In113 and stain erythrocytes), and are labeled as “[all species]”, “[primates]/[mice]”, or “[humans]/[non-human-primates]/[mice]”.

To assess potential differences in phenotypes of the orthologous cell populations between species, we plotted the distributions of staining intensities of every surface marker in every species by population and provide this data as a reference for future studies (**Figures 4**, **S3**). While these populations express many of the same markers across species, a qualitative analysis found many potential differences. For example, neutrophils in humans express high levels of CD16 and moderate levels of CD11c, in contrast to all three NHP species; meanwhile, neutrophils in the three NHP species express higher levels of CCR7 and neutrophils in mice express moderate levels of CD16/32 (**Figure 4**). B cells in macaques express higher levels of CD1c than in humans or African green monkeys, as discussed later. CD4+ and CD8+ T cells in humans express higher levels of CD161 and CD7 than NHPs, while mouse T cells do not express NK1.1 (CD161). NK cells in NHPs express higher levels of CD8 than humans; this is consistent with Autissier et al. (18), although one must not ignore the fact that some human NK cells also express CD8 (39). Classical monocytes in African green monkeys stain brightly for BDCA3—a canonical dendritic cell subset marker in humans (**Figure S3**).

Differences in staining intensities should be interpreted with caution because genomic variation of antigens may affect binding affinity between species. One must also consider the possibility for differences in cross-reactivity with different epitopes and populations in NHPs than in humans, against which most of the primate panel antibodies were raised. For this reason, we primarily focus analysis on cases where a marker is present in one or more species and essentially absent in another, or cases that we can corroborate through further data mining and literature review.

### Species-Specific Signaling Profiles Have Implications for Drug Development

**Figure 5:**
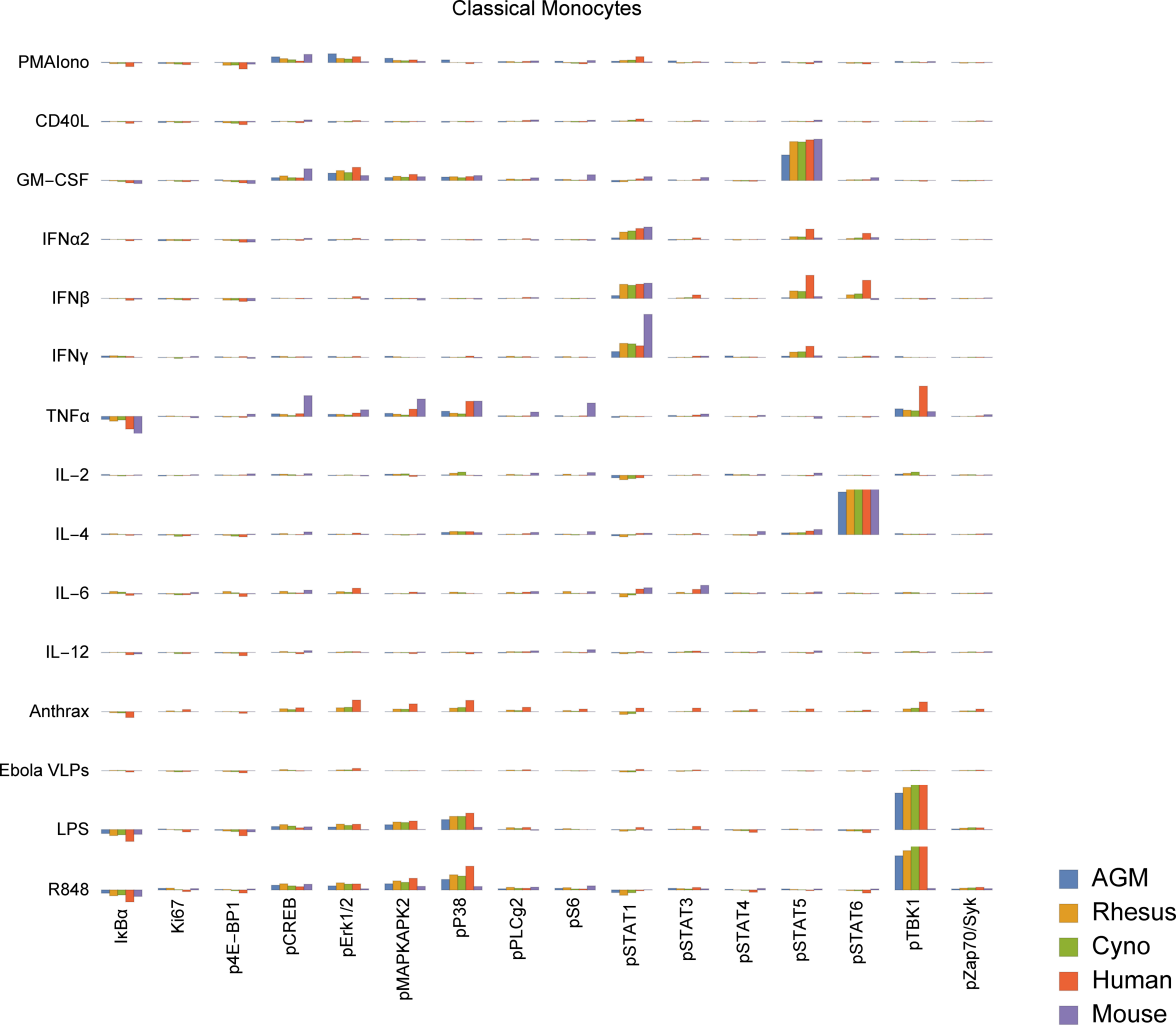
Signaling responses (difference of ArcSinh-transformed values; approximately equivalent to fold-change) in classical monocytes by stimulus, activation marker and species (other cell types in **Figure S4**). Note that *Bacillus anthracis* (“anthrax”) and Ebola VLPs were not available for use as stimuli in mice or AGMs; thus, values for these species are always displayed as zero. We could not gate intermediate monocytes or a “CD11b−/CD16−”-equivalent population in mice; these values are also zero. The Y axis range of all charts is -0.5 to +1.5.

To study functional differences of orthologous cell types, we visualized signaling behaviors by species (**Figures 5**, **S4**). These charts are meant to provide a compact, semi-quantitative overview as a reference point for future studies; full tables of marker medians by donor and population are provided in **Tables S8**, **S9**. It is important to recognize that because of the large size of this dataset, individual cell signaling observations require validation in subsequent studies to fully rule out false discovery. Nevertheless, we explore here and in the subsequent sections many potential differences in signaling behavior and how they relate to existing literature. In terms of innate immune responses, we observed an apparent absence of TLR7/8 (R848) and TLR4 (LPS) responses in mouse classical monocytes, as evidenced by a lack of pTBK1, pP38 and pMAPKAPK2 signaling. The TLR7/8 (R848) results are not unexpected since prior literature indicates that mouse TLR8 is defective (40), and TLR7 is primarily expressed in DCs (41) (and, consistent with that, we saw a response to R848 in mouse pDCs). The lack of TLR4 signaling in mouse monocytes is a potentially novel observation; expression and functionality of TLR4 in mouse blood monocytes under basal conditions is unclear from prior literature but are known to have significant differences from humans (42, 43). Considering these findings in addition to the fact that mouse monocytes essentially lack MHC-II and are thus not major antigen-presenting cells (44, 45), it is clear that classical monocytes, which are crucial to innate immune responses in humans, may serve a very different role in mice.

We also observed that all three NHP species appear to have an almost-absent pSTAT6 response to IL-4 in non-classical monocytes. This is in sharp contrast to humans and mice, where pSTAT6 responses to IL-4 are a major, canonical signaling response. Mice deficient in STAT6 are known to exhibit defective immune behaviors in response to IL-4 across the spectrum (46, 47). Despite the almost-absent response in non-classical monocytes, all three NHP species appear to have varying degrees of pSTAT6 responses to IL-4 in other cell types, including T cells, classical monocytes, intermediate monocytes, neutrophils, B cells, DCs and NK cells. These responses serve as positive technical controls for STAT6 and IL-4 in NHPs in our assays, but may also point towards biological compensation for the lack of response in non-classical monocytes in NHPs.

Finally, we observed that African green monkey pDCs appear to have essentially no IκBα response to R848, although they have a stronger pTBK1 response than any of the other species tested. Meanwhile, cynomolgus pDCs may have a unique pSTAT5 response to IL-6. Others have reported that IL-6 causes low-levels of phosphorylation of STAT5 in T cells and NK cells in mice to detectable levels by 15 minutes (48) (the time point we used); we too see a small response in mice and humans in those cell types, but the magnitude of these responses are dwarfed by that of cynomolgus pDCs.

These results highlight the importance of careful selection and interpretation of animal models for drug development and evaluation, as the substantial signaling differences between species could provide misleading results in terms of drug responsiveness at the cellular level. The remainder of this report details several specific examples of potential differences between species in B cells, T cells, granulocytes and monocytes, particularly focusing on several cell types and behaviors unique to macaques.

### Macaque Monocyte Abundance Correlates With Herpes Simian B Virus Status

Many macaques are infected with herpes simian B virus (formerly Cercopithecine herpes virus 1, or B virus) by natural exposure. Like herpes simplex viruses in humans, the virus is community or sexually acquired, persists for life and is essentially harmless to macaques. Whether or not herpes serostatus has an effect on general immunological health is unknown, and a vast number of published macaque studies do not report the serostatus of the animals or report using SPF animals.

**Figure 6:**
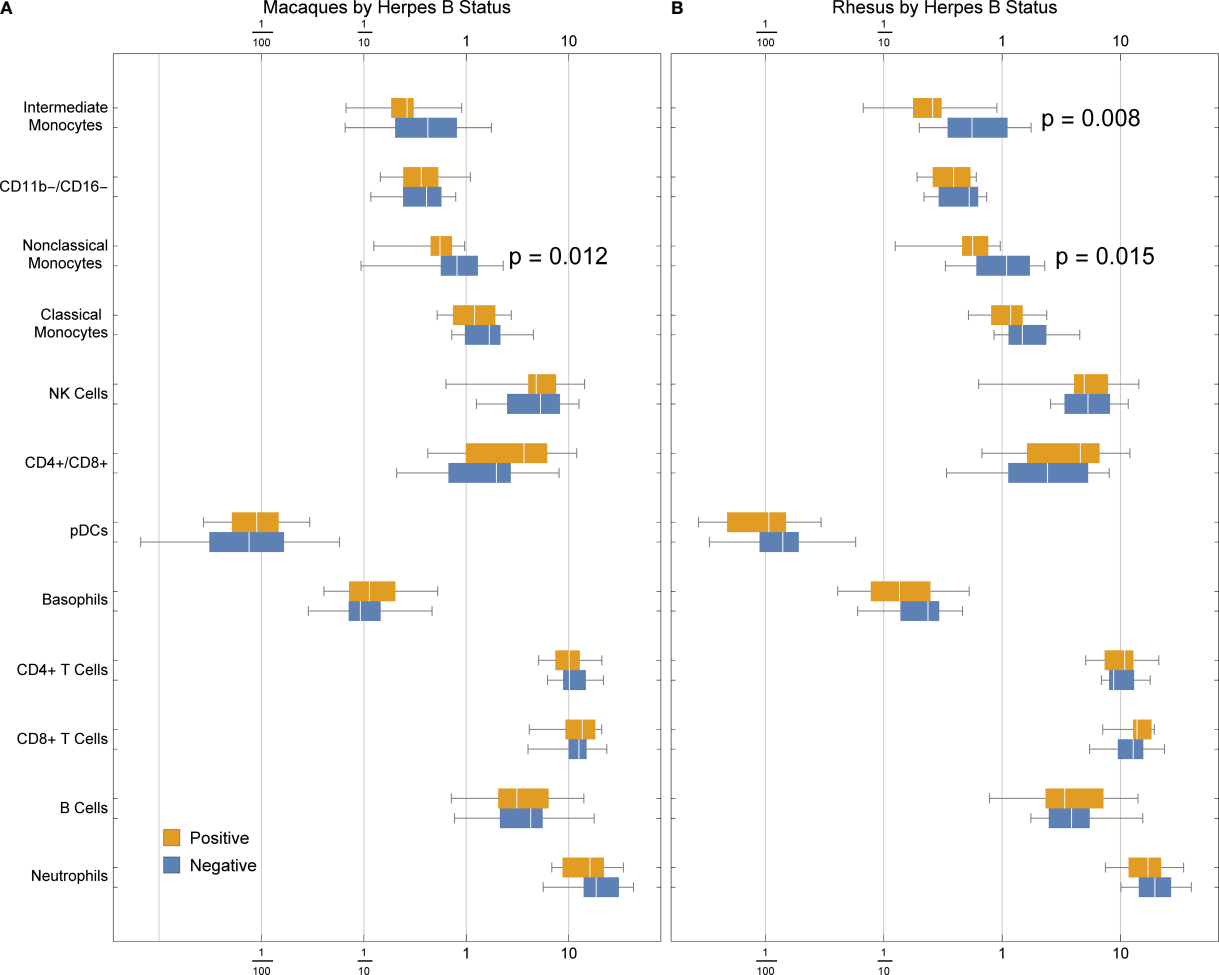
Frequencies of cell types in **(A)** rhesus and cynomolgus macaques or **(B)** rhesus macaques only, by herpes B virus status. P values were calculated between serostatus groups using a one-tailed Mann-Whitney U test.

We detected a significant association between B virus status and non-classical monocyte abundance in all macaques (**Figure 6A**), and a stronger association with both intermediate and non-classical monocyte frequency in rhesus macaques (**Figure 6B**). This finding suggests that herpes simian B virus serostatus can indeed affect immune function and may thus factor into innate immune responses observed during therapeutic evaluations performed in macaques.

### CD4+CD8+ Double-Positive T Cells Are More Abundant in Macaques

Macaques are known to have higher frequencies of peripheral CD4+CD8+ double-positive (DP) T cells than humans, the frequency of which furthermore increases with age (**Figure S5**). We found a median frequency of 5.3% in rhesus, 1.4% in cynomolgus and less than 0.2% in AGM, mouse and human (**Figure S5**).

### Macaque Granulocytes Are Rapidly Responsive to *Bacillus anthracis*

**Figure 7:**
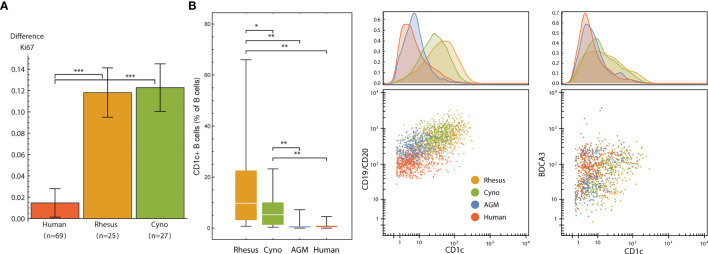
**(A)** Neutrophil Ki67 induction (mean and standard error of differences of ArcSinh-transformed values) by species after exposure to 22M CFU of gamma-irradiated *Bacillus anthracis* for 15 minutes. **(B)** CD1c+ B cells are more abundant in non-human primates than in humans, and CD1c is furthermore expressed at higher levels in NHP B cells, especially in macaques. Left: Abundance of CD1c+ B cells (expressed as % of total B cells) in each species. Middle and right: One representative individual from each species. Dot plots show 500 randomly selected B cells (middle) or 250 randomly selected CD11b-/CD16- DCs (right). Statistics **(A, B)**: Groups were compared using a one-tailed Mann-Whitney U test with asterisks indicating significant differences (*p < 5x10^-2^, **p < 1x10^-2^, ***p < 1x10^-3^).

We evaluated the signaling response to a 15-minute incubation with 22 × 10^6^ CFU of gamma-irradiated (inactivated), vegetative *Bacillus anthracis* Ames. Despite the short incubation period, we found a subtle, but significantly greater, level of neutrophil activation (Ki67) in macaques than in humans (**Figure 7A**). This indicates a very rapid response to infection in these animals; humans could lack this response outright or have delayed kinetics.

That this activation occurs in neutrophils is especially interesting not only because neutrophils kill *B. anthracis* (49), but also because macaque and several other NHP species’ neutrophils uniquely contain theta defensins (50), which are highly potent antibiotics against *B. anthracis* and its lethal factor (LF) (51, 52). This finding is particularly important considering that anthrax therapeutics are among those that are candidates for evaluation under The Animal Rule, with efficacy studies conducted in animal models.

For reasons beyond our control(Committee for Comprehensive Review of DoD Laboratory Procedures, 2015), we discontinued use of *Bacillus antigen* midway through the project; thus, none of the mouse or AGM samples were treated with *Bacillus* antigen, nor were 18 of the human samples. Accordingly, our analyses considered only the samples that were treated.

### Macaques Have Unique CD1c+ and CD8+ B Cell Subsets

We observed two unique subsets of B cells in macaques defined by either CD1c or CD8α expression. These populations were either rare or absent in humans, mice and African green monkeys.

CD1 is a family of lipid and glycolipid-presenting molecules—a counterpart to MHC class I and II found on a subset of B cells and dendritic cells that plays an important role in humans in defense against diseases such as tuberculosis. CD1c (BDCA-1) specifically presents mannosyl mycoketide and phosphomycoketide (53). A previous study reported that 21.4% of B cells in rhesus macaques were CD1c+ B cells, in contrast to humans with only 3.3% (18). We similarly found significantly more CD1c+ B cells in all three NHP species that we examined when compared to humans, and significantly higher amounts of CD1c therein (**Figure 7B**). Interestingly, mice and rats lack group 1 CD1 altogether (53). Mouse susceptibility to tuberculosis varies by strain, but at least several, including the common C57BL/6 and BALB/c strains, are resistant (54), and thus must have group 1 CD1-independent mechanisms for controlling infection. With regard to the higher intensity of CD1c staining, we initially considered that this could be due to a difference in antibody affinity between species; however, the levels of CD1c on DCs were similar between macaques and humans (**Figure 7B**).

In humans, CD8α is found almost exclusively on T cell and NK cell subsets; exceptions are limited to conditions such as HIV-1 (55), B-cell leukemia (56–58) and lymphomas (59), and potentially a very small subset in healthy individuals (55). In many rhesus macaques, however, CD8α is also found on a subset of B cells (24). We found 19 out of 25 animals had at least 0.5% of their B cells stain for CD8 and some animals had as much as 33% of B cells stained (mean: 6.5%, median: 3.3%) (**Figure S6A**). We gated these cells as CD45+ CD66− CD3− CD20+ CD7− CD8+, thereby excluding T cells and NK cells, which also stains for CD8 in macaques. We continued to determine that cynomolgus macaques, African green monkeys and mice do not appear to have CD8+ B cells (**Figure S6B**). Because we simultaneously measured phenotyping and signaling molecules, we were also able to evaluate the functional behavior of CD8+ B cells (**Figure S6C**). Considering several higher-magnitude responses, we found that these cells still display the hallmark signaling behaviors of CD8- B cells, albeit perhaps with stronger pSTAT1 signaling.

These findings are thus potentially important criteria for selecting not only which species to use for model development and therapeutic evaluation but given the high variance of CD8+ B cell frequency within rhesus macaques, which individual donors.

## Discussion

Animal models are commonplace in drug development, but are imperfect and may result in misleading false positives, when drugs work in models but fail in humans, and false negatives, when drugs fail to work in models but would work in humans. Aside from the economic and ethical burden, this creates a public health problem by diminishing researchers’ abilities to develop therapeutics and countermeasures for emerging diseases. To successfully develop the next generation of targeted therapeutics that affect specific pathways and cell types rather than a broad activation of the immune system or the infectious agent itself, careful selection of relevant models will be required. Towards that goal, we have created a comprehensive atlas of immunological differences between humans, mice, and non-human primates.

To construct this atlas we first substantially expanded the breadth and depth of available antibody cross-reactivity data between primate species, and have deposited the cross-reactivity dataset containing the primary flow cytometry files online so that any user can easily compare staining patterns in these species, and even look at subpopulations of cells to check specificity—information not typically reported in the NIH database (nhpreagents.org).

As a result of evaluating specific cell types, we have identified numerous antibodies that stain different populations in non-human primates than in humans. In light of this, researchers should take adequate steps to ensure they are evaluating the intended populations when using these reagents, and when evaluating immunotherapeutics targeting these proteins.

While we screened 332 different anti-human antibody clones, not all of the antigens targeted by those antibodies are expected to be present in the resting, peripheral blood cells that we tested. Indeed, only 78.3% (260/332) of the antibodies positively stained human blood. Thus, our screen did not evaluate markers that are found only in cells from bone marrow and other tissues, in progenitors, or in activated populations. Furthermore, antibodies that stain rare populations—especially populations that comprise less than 10 percent of one of the populations that we delineated, or that show dim expression that might be excluded by the threshold for expression and staining that we applied. We encourage diligence when interpreting markers such as CD41 and CD51/CD61, which are listed as reactive with all cell types, but in actuality are probably staining platelet fragments stuck to other cells, based on the known distribution of those markers in humans (60) (also see *Discussion* below). It is also important to keep in mind that actual antibody-antigen specificity may vary by species due to differences in gene sequence, protein structure, and post-translational modifications.

The cross-reactivity data described here are especially valuable for the advancement of African green monkey (AGM) immunology because there is a dearth of literature discussing immunophenotyping and only 28 reactive antibodies listed in the NIH database. Nonetheless, this species is important in drug development—especially SIV research—and is the subject of an international effort to make it the most comprehensively characterized NHP by phenotype and genomics (61). Notably, the usage of AGMs is increasing due to a shortage of rhesus macaques for research (62).

In the analysis of cell phenotypes presented here, we focused on high-confidence differences between species. There are several observed potential differences that we did not discuss for specific reasons: (a) While human NK cells, B cells, and non-classical monocytes appear to express higher amounts of CD45RA, we previously observed what could be a difference in affinity for this clone between NHPs and humans. (b) Because humans were stained with anti-CD19 and NHPs with anti-CD20, we cannot necessarily conclude that the difference in staining of these B cell markers is significantly different. (c) As discussed earlier, although CD11c is known to exist in AGMs (21), no CD11c clone could be found that is cross-reactive with AGMs and sensitive enough for our CyTOF panel. This marker is thus negative in all populations in AGMs. (d) The moderate, wide-spread staining of CD61, which canonically stains platelets, monocytes, and macrophages, could be due to platelet debris sticking to other cell types during processing and thus have little physiological meaning. However, this marker has also been proposed to be acquired by activated T cells from platelet-derived microvesicles (63), and in previous studies with cynomolgus macaque blood, we observed subsets of T cells, B cells, and NK cells that stain for CD61 (not shown), which could indicate that this is an activation marker in more than just T cells in macaques.

One must also take into consideration necessary technical caveats associated with dosing and cytokine activity before making precise, quantitative conclusions about signaling assays: When available, we used cytokines specific to each species, and we dosed at the same mass concentration (i.e. mg/mL) as we dosed for humans as opposed to attempting to coordinate species-specific ECx values. Thus, quantitative comparisons must be verified by close examination of controls and/or by performing additional experiments such as dose-response curves. To that end, we advise comparison with other signaling antibodies, stimulation conditions and/or other cell types within the dataset, which can usually serve as internal controls. Also, because of the very large size of this dataset and the potential for false discovery, individual cell signaling observations should be treated with caution prior to validation in subsequent studies.

While one cannot completely discount the complex interplay between multiple pathways and multiple cell types, a therapeutic targeting one pathway in one cell type should be confirmed to trigger relevant, similar signaling in humans as in the model species and evaluation of as many pathways as possible should be carefully considered. Here we show that there are conserved and differential qualities between species that must be carefully considered based on their relevance to the experiment at hand and provide a reference of these characteristics between species. The submitted dataset may be viewed online, and the accompanying article by Fragiadakis, et al. (17) provides a detailed analysis of the human data, including correlations with demographics and signaling networks.

Given immunological differences between species reported here, we feel researchers should continue to consider humans as early as possible in the drug development process, including in initial planning, screening, and evaluation stages. For example, using primary human cells (e.g. blood and tumor samples) in places of cell lines and cells from other species, as well as evaluating differences in mechanisms of disease and therapeutics between humans and model organisms will greatly improve the evaluation of therapeutic candidates.

## Materials and Methods

### Blood

Venous human blood was obtained from the Stanford Blood Center, AllCells Inc. (Alameda, CA) (exempt, non-human subjects research) or from volunteers from the Stanford community under an IRB-approved protocol (#28289).

All animal blood was collected under an approved animal care and use protocol. Macaque blood (*Macaca mulatta* and *M. fascicularis*) was obtained from Valley Biosystems, Inc. (location withheld) from conscious (not sedated), captive-born, Chinese-origin animals. Health reports for the macaques were obtained and are available in the experiment data repository. African green monkey (*Cercopithecus aethiops*) blood was obtained from Bioreclamation, LLC (Westbury, NY) and Worldwide Primates, Inc. (location withheld). The African green monkeys were wild-born in St. Kitts; age was estimated by capture date (assuming two years old at time of capture). Baboon blood was obtained from the Southwest National Primate Research Center, which is funded by the National Center for Research Resources (p51 RR013986) and supported by the Office of Research Infrastructure Programs/OD P51 OD011133. Mice were obtained from Charles River Laboratories. Animal work was done under an approved animal care and usage committee protocol (#26675). Complete blood counts (CBCs) were performed on a Sysmex XT-2000iv with the veterinary software module.

For all species, whole blood was collected in sodium heparin tubes, stored/shipped at ambient temperature (with insulation to protect from temperature changes) and processed within 24 hours of collection for the flow cytometry antibody screen or stimulation and staining for mass cytometry as described below.

### Flow Cytometry Antibody Screen

Eight to 10 ml of blood was gently fixed and lysed by incubating with 792 µl of 16% paraformaldehyde (Electron Microscopy Sciences, Hatfield, PA; final concentration approx. 0.3%) and 29.6 ml of VersaLyse (Beckman Coulter, Brea, CA) for 10 minutes at room temperature, then washed once with 0.01% BSA in PBS (“staining buffer”). Cells were resuspended in 6.5 ml of staining buffer, then incubated with 0.5 ml of human TruStain FcX Fc receptor blocking solution (Biolegend, San Diego, CA) for 10 minutes.

Five counterstain antibodies were then added: 500 µl CD3 (SP34.2)-Brilliant Violet 421 (BD Biosciences, San Jose, CA), 500 µl CD20 (2H7)-Brilliant Violet 605 (Biolegend), 1000 µl CD66 (TET2)-APC-Vio770 (Miltenyi, San Diego, CA), 500 µl CD11b (ICRF44)-PerCP/Cy5.5 (Biolegend) and 500 µl CD7 (M-T701)-APC (BD Biosciences), for a final volume of 10 ml.

Subsequent steps were performed on an automation platform including an Agilent Bravo 96-channel pipetting robot, centrifuges, BioTek ELx405 96-channel aspirator/dispenser and Thermo Scientific MultiDrop dispenser. Twenty microliters of cells in the antibody cocktail were dispensed into every well of a 384-well plate using the MultiDrop. LegendScreen Human PE antibody screen plates (Biolegend) were rehydrated with the manufacturer-recommended 25 µl of water. The screen consists of four 96-well plates; 5 µl from each well were transferred to the single 384-well plate quadrant-wise. Staining reactions were incubated in the dark at room temperature for 30 minutes with 2.0 mm-radius orbital shaking, then washed three times with staining buffer and acquired on a BD LSR II with 405, 488 and 633 nm lasers using an HTS autosampler.

Files were gated and populations exported using CellEngine (https://cellengine.com ; CellCarta; Montreal, QC). Every well was manually gated by time to exclude anomalies caused by air bubbles or debris: temporal regions where the signal in the PE channel over time were inconsistent were excluded. Cell populations were then gated as described in the Results section. Population gates were tailored to each species; tailoring to individual donors within a species was not necessary. The remaining analysis of the antibody screen (statistics calculations, discordant replicate resolution, verification and reporting) was performed using *Mathematica* (Wolfram Research, Champaign, IL).

#### Stimuli

Stimuli used are shown in **Table S5**. Human and non-primate stimuli were tested in human whole blood over a range of concentrations to select the working concentration. Mouse stimuli were likewise tested in mouse blood. All stimuli were diluted such that the same volume of each would achieve the desired stimulation concentration, then aliquoted into single-use stimuli plates and stored at -80, with the exception of those marked with *, which were dispensed at time of use due to storage requirements or the need to adjust the stimulation concentration. All cytokines were tested for endotoxin by the LAL method and verified to contain an amount less than that detectable by our phospho-flow assays (approximately 10 pg/ml) (data not shown).

LPS was commercially prepared by phenol-water extraction and contained small amounts of other bacterial components that activate TLR2. The particular type of LPS (long chain derived from *E. coli* O111:B4) was selected because it is from a pathogenic strain more commonly found in clinical cases (64).

Rhesus/cynomolgus IL-2 was obtained from the NIH/NCRR-funded Resource for Nonhuman Primate Immune Reagents.

Gamma-inactivated vegetative *Bacillus anthracis* Ames (ANG-BACI008-VE) was obtained from the Department of Defense Critical Reagents Program through the NIH Biodefense and Emerging Infections Research Resources Repository, NIAID, NIH.

Zaïre Ebolavirus-like particles were used within 48 hours of production, except where noted, and thus different lots were used throughout the course of the project. Particles were produced according to (Johnson et al., 2006) using calcium phosphate instead of Lipofectamine. Briefly, 1 µg of pCAGGS-GP, 1 µg of pCAGGS-VP35, 1.5 µg of pCAGGS-NP and 1.5 µg of pCAGGS-VP40 (courtesy of R. Johnson et al., NIH NIAID Integrated Research Facility) were transfected into 293T cells by the calcium phosphate method, harvested after 36 hours, then purified through a 20% sucrose cushion at 115,605 x *g* for two hours and stored at +4 degrees. The proteins self-assemble into a structure resembling the native virion (65, 66). These particles are inherently replication-defective and when administered to non-human primates evoke a vaccinating immune response (67–69), furthermore eliciting type I interferon and proinflammatory cytokine expression (70). To validate that the production method worked in our hands, preparations were verified by western blot to contain each of the four proteins and by electron microscopy for morphology and quantity. Subsequent preparations were spot-checked by electron microscopy. For particle quantitation and morphological observation, VLP particle preparations were mixed with 110 nm latex spheres (Structure Probe, Inc., West Chester, PA) at a known concentration, absorbed onto copper carbon-Formvar-coated 300 mesh electron microscopy grids prepared in duplicate and stained with uranyl acetate (protocol courtesy of J. Birnbaum, NIH NIAID Integrated Research Facility). Grids were imaged on a JEOL JEM1400 (funded by NIH grant 1Z10RR02678001). Due to the short shelf-life of the particles, grids were generally imaged after use as a stimulus.

### Mass Cytometry Antibodies

Purified antibodies were purchased and conjugated in-house using DVS/Fluidigim MaxPar X8 metal conjugation kits (**Table S2**). All antibodies were titrated for optimal signal-to-noise ratio, then re-confirmed in at least two different individuals per species (three humans, two cynomolgus macaques, two rhesus macaques, three mice). All conjugations and titrations were well-documented, and records are available upon request. Finally, antibodies were lyophilized into LyoSpheres by BioLyph LLC (Hopkins, MN) with excipient B144 as 4x cocktails. CyTOF antibody LyoSpheres were stress-tested for over one year and found to have no significant change in staining (not shown).

### Stimulation and Staining for Mass Cytometry

Stimulation and staining was carried out on a custom automation platform consisting of an Agilent Bravo pipetting robot, Agilent BenchBot robotic arm, Peak KiNeDx robotic arm, Thermo Cytomat C2 incubator, BioTek ELx405-UVSD aspirator/dispenser, BioTek MultiFlo FX four-reagent dispenser, Q.Instruments microplate shakers, Velocity11 VSpin centrifuges and a custom chilling system contained in a negative-pressure biosafety enclosure. The VWorks robotic programs and logs from protocol runs are available upon request.

Whole blood (330 𝜇l) was stimulated by adding to and mixing with stimuli (20 𝜇l) and incubating in a humidified 37-degree, 5% CO_2_ incubator for 15 minutes. Blood was fixed for 10 minutes at room temperature with 1.6% paraformaldehyde (PFA, Electron Microscopy Sciences) and lysed with 0.1% Triton-X100 in PBS for 30 minutes at room temperature per (71). Cells were washed twice with PBS, then each donor’s 16 conditions were barcoded according to (72, 73). Briefly, cells were permeabilized with 0.02% saponin, then stained with unique combinations of functionalized, stable palladium isotopes. The stimulation plate containing 6 donors x 16 conditions was then reduced to 6 wells, each containing the 16 conditions for one donor. Cells were washed once with staining media (CSM: 0.2% BSA in PBS with 0.02% sodium azide), blocked with human (humans, NHPs) or mouse (mice) TruStain FcX block (Biolegend) for 10 minutes at room temperature with shaking, then stained with rehydrated extracellular LyoSpheres for 30 minutes at room temperature with shaking in a final volume of 240 µl. (See **Table S2** for final staining concentrations.) Cells were washed once, then permeabilized in >90% methanol at 4 degrees C for 20 minutes. Cells were washed four times, then stained with intracellular lyospheres for 60 minutes at room temperature with shaking. Cells were washed once, then placed into 1.6% PFA and 0.1 µM natural iridium intercalator (Fluidigm) in PBS at 4 degrees C until acquisition on a CyTOF. With few exceptions, cells were acquired within seven days of staining. From prior validation experiments, this amount of time imparts no significant effect on staining.

### Mass Cytometry Acquisition

Prior to running, cells were washed twice with water. Samples were acquired on a single DVS/Fluidigm CyTOF 2 fitted with a Super Sampler sample introduction system (Victorian Airship & Scientific Apparatus LLC). QC reports were run on the CyTOF between every barcoded sample. Prior to beginning acquisition, the instrument must have demonstrated Tb159 dual counts > 1,000,000 and oxidation < 3%; if the instrument failed those criteria, it was cleaned, tuned or repaired as necessary. Approximately 4,800,000 events were acquired per sample. Data were normalized and debarcoded using the data normalization software (74) and the single cell debarcoder tool (73) as previously described. Data were then uploaded to CellEngine for analysis.

### Statistics

Statistical methods are described throughout the text and figure legends. Box plots, center line shows mean value; lower and upper box limits, lower and upper quartiles, respectively; and whiskers 1.5 x interquartile range. Tests between pairs were performed using one-tailed Mann-Whitney U tests, tests between groups were performed using ANOVA with Bonferroni correction for multiple hypotheses. A p-value less than 5x10^-2^ was considered significant. *p < 5x10^-2^, **p < 1x10^-2^, ***p < 1x10^-3^.

## Data Availability Statement

The datasets presented in this study can be found in online repositories. The names of the repository/repositories and accession number(s) can be found below: Flowrepository, accession number FR-FCM-Z2ZY, https://flowrepository.org/id/FR-FCM-Z2ZY.

## Ethics Statement

The studies involving human participants were reviewed and approved by Stanford University Institutional Review Board. The patients/participants provided their written informed consent to participate in this study. The animal study was reviewed and approved by Stanford University Administrative Panel on Laboratory Animal Care (APLAC).

## Author Contributions

ZB-H generated the data, performed the analysis and wrote the manuscript. GF contributed to data generation and edited the manuscript. MS contributed to data generation and edited the manuscript. DM, KH, and KL contributed to data generation and reagent optimization. HC and DRM edited the manuscript and advised on analysis. GN advised the study and edited the manuscript. All authors contributed to the article and approved the submitted version.

## Funding

The research discussed in this article was supported in part by the U.S. Food and Drug Administration (Contract No. HHSF223201210194C). Additional support was provided by NIH awards 5R01CA18496804, 5R25CA18099304, 1R01GM10983604, 5UH2AR06767603, 1R01NS08953304 and R01HL120724, and FDA contract HHSF223201610018C. ZB-H was supported in part by NIH grant T32GM007276. GF was supported in part by a Stanford Bio-X graduate research fellowship and NIH grant T32GM007276. MS was supported in part by NIH grant DP5OD023056.

## Author Disclaimer

This article reflects the views of the authors and should not be construed to represent the U.S. Food and Drug Administration or NIH’s views or policies.

## Conflict of Interest

Author KL was employed by BioLegend Inc.

The remaining authors declare that the research was conducted in the absence of any commercial or financial relationships that could be construed as a potential conflict of interest.

## Publisher’s Note

All claims expressed in this article are solely those of the authors and do not necessarily represent those of their affiliated organizations, or those of the publisher, the editors and the reviewers. Any product that may be evaluated in this article, or claim that may be made by its manufacturer, is not guaranteed or endorsed by the publisher.

## Supplementary Material

Supplementary Figure 1
**(A)** Five counterstains allow identification of at least five cell populations. Representative staining from a baboon shown. After gating by time to exclude artifacts, granulocytes were identified as CD66abce+/SSC-A+. Non-granulocytes were divided into B cells (CD20+/CD3−), T cells (CD3+/CD20−), NK cells (CD7+/CD3−/CD20−) and monocytes/dendritic cells (CD7−/CD3−/CD20−). *In species other than the African green monkey, dendritic cells could be separated on the basis of CD11b staining. **(B)** CD172g is expressed on monocytes and all granulocytes, but not on T cells, in examined NHP species. By comparison, CD172g is expressed on all T cells, a subset of granulocytes and a subset of B cells (data not shown) in humans. As discussed in the text, CD11b did not uniformly stain AGMs; thus, some of the CD11b-negative cells are monocytes. Blue: isotype control, orange: CD172g. **(C)** CD2 is expressed on B cells in NHPs. Blue: CD3+ T cells where expression is expected in humans; orange: CD20+ B cells.

Supplementary Figure 2 Universal phenotyping panels enable parallel gating in humans, macaques, African green monkeys and mice.

Supplementary Figure 3 Continuation of **Figure 4**: Distribution of surface marker expression for each species in all cell types.

Supplementary Figure 4 Continuation of **Figure 5**: Signaling responses in all cell types by stimulus, activation marker, and species.

Supplementary Figure 5 Frequency distribution of CD4+CD8+ double positive T cells for rhesus and cynomolgus macaques. Table: Median frequencies (percent of singlet cells) by species.

Supplementary Figure 6
**(A)** Histogram of percentages of B cells staining for CD8 in rhesus macaques. Population defined as CD45+ CD66− CD3− CD20+ CD7− CD8+. **(B)** CD8 staining in B and T cells for representative animals of each species. **(C)** Selected mean signaling responses in rhesus macaques with CD8+ B cells. cells (1) still respond to the canonical B cell stimulus CD40L by phosphorylating CREB and degrading IκBα, albeit to a lesser degree than CD8− B cells; (2) unlike T and NK cells, do not degrade IκBα in response to TNFα, do not phosphorylate STAT5 in response to IL-2 and do not phosphorylate STAT4 in response to IFNβ; and (3) have 47% greater pSTAT1 response to IFNβ than CD8−B cells.

Supplementary Table 1 Summary of cell type-specific cross-reactivity. Clones were considered reactive if at least 10 percent of cells had a signal greater than the signal of the 95th percentile of corresponding isotype control or if they were manually classified as positive. Presence of letter in column indicates reactive staining detected in that cell population, absence of letter indicates clone is unreactive in that cell population. B, B cells; T, T cells; N, CD7+ NK cells; M, CD7- monocytes and dendritic cells; G, granulocytes,?: inconclusive, insufficient number of cells acquired for a given population to make a clear assessment. Two of each non-human primate species and one human were assayed.

Supplementary Table 2 CyTOF Panel. Sheet 1-3: Extracellular antibody, clone, label, and staining concentrations for humans (sheet 1), macaques (sheet 2), AGMs (sheet 3), and mice (sheet 4). Sheet 5: Signaling and other post-permeabilization antibodies. *At time of rehydration, surface cocktail was supplemented with 8.64 µl of CD4 #201 (OKT4) per 12 LyoSpheres to increase signal.

Supplementary Table 3 Studies reporting frequencies of DP T cells in macaques. *SIV infected. †denotes frequencies calculated from Lee et al.’s Table 1; we report the mean of the means for each of the DP-high/middle/low-frequency groups to correct for bias arising from unequal group sizes.

Supplementary Table 4 Demographics of human donors. Age (years), height (cm), weight (kg), and BMI information of 39 females and 44 male human donors. For one individual, do not have additional information on.

Supplementary Table 5 Stimuli. Stimuli marked with an asterisk (*) were dispensed at time of usage due to production and/or storage requirements.

Supplementary Table 6 Population counts and frequencies for 13 populations for all primate samples.

Supplementary Table 7 Population counts and frequencies for 10 populations for all mouse samples.

Supplementary Table 8 Functional marker (e.g., phospho-specific antibody) medians for 13 populations for all primate samples.

Supplementary Table 9 Functional marker (e.g., phospho-specific antibody) medians for 10 populations for all mouse samples.

## References

[1] FDA. US Food and Drug Administration: Animal Rule Information. Available at: https://www.fda.gov/emergency-preparedness-and-response/mcm-regulatory-science/animal-rule-information (Accessed December 15, 2021).

[2] Davis MM. A Prescription for Human *Immunology* . Immunity (2008) 29:835–8. doi: 10.1016/j.immuni.2008.12.003 PMC2905652 19100694

[3] Bente D Gren J Strong JE Feldmann H. Disease Modeling for Ebola and Marburg Viruses. Dis Model Mech (2009) 2:12–7. doi: 10.1242/dmm.000471 PMC2615158 19132113

[4] Richmond A Su Y. Mouse Xenograft Models vs GEM Models for Human Cancer Therapeutics. Dis Model Mech (2008) 1:78–82. doi: 10.1242/dmm.000976 19048064 PMC2562196

[5] Rogers KA Scinicariello F Attanasio R. IgG Fc Receptor III Homologues in Nonhuman Primate Species: Genetic Characterization and Ligand Interactions. J Immunol (2006) 177:3848–56. doi: 10.4049/jimmunol.177.6.3848 16951347

[6] Rogers KA Scinicariello F Attanasio R. Identification and Characterization of Macaque CD89 (Immunoglobulin A Fc Receptor). Immunology (2004) 113:178–86. doi: 10.1111/j.1365-2567.2004.01949.x PMC1782566 15379978

[7] Nimmerjahn F Ravetch JV. Fcγ Receptors as Regulators of Immune Responses. Nat Rev Immunol (2008) 8:34–47. doi: 10.1038/nri2206 18064051

[8] Seok J Warren HS Cuenca AG Mindrinos MN Baker HV Xu W. Genomic Responses in Mouse Models Poorly Mimic Human Inflammatory Diseases. Proc Natl Acad Sci (2013) 110:3507–12. doi: 10.1073/pnas.1222878110 PMC3587220 23401516

[9] Barreiro LB Marioni JC Blekhman R Stephens M Gilad Y. Functional Comparison of Innate Immune Signaling Pathways in Primates. PloS Genet (2010) 6:e1001249. doi: 10.1371/journal.pgen.1001249 21187902 PMC3002988

[10] Shay T Jojic V Zuk O Rothamel K Puyraimond-Zemmour D Feng T. Conservation and Divergence in the Transcriptional Programs of the Human and Mouse Immune Systems. Proc Natl Acad Sci (2013) 110:2946–51. doi: 10.1073/pnas.1222738110 PMC3581886 23382184

[11] Eastwood D Findlay L Poole S Bird C Wadhwa M Moore M. Monoclonal Antibody TGN1412 Trial Failure Explained by Species Differences in CD28 Expression on CD4+ Effector Memory T-Cells. Brit J Pharmacol (2010) 161:512–26. doi: 10.1111/j.1476-5381.2010.00922.x PMC2990151 20880392

[12] Yasuda S Zhang L Huang S. The Role of Ethnicity in Variability in Response to Drugs: Focus on Clinical Pharmacology Studies. Clin Pharmacol Ther (2008) 84:417–23. doi: 10.1038/clpt.2008.141 18615002

[13] Gandhi M Aweeka F Greenblatt RM Blaschke TF. Sex Differences In Pharmacokinetics And Pharmacodynamics. Annu Rev Pharmacol (2004) 44:499–523. doi: 10.1146/annurev.pharmtox.44.101802.121453 14744256

[14] Uchida N Hargrove PW Lap CJ Evans ME Phang O Bonifacino AC. High-Efficiency Transduction of Rhesus Hematopoietic Repopulating Cells by a Modified HIV1-Based Lentiviral Vector. Mol Ther (2012) 20:1882–92. doi: 10.1038/mt.2012.159 PMC3464651 22871664

[15] Brown KN Barratt-Boyes SM. Surface Phenotype and Rapid Quantification of Blood Dendritic Cell Subsets in the Rhesus Macaque. J Med Primatol (2009) 38:272–8. doi: 10.1111/j.1600-0684.2009.00353.x PMC3073502 19344375

[16] Carter DL Shieh TM Blosser RL Chadwick KR Margolick JB Hildreth JEK. CD56 Identifies Monocytes and Not Natural Killer Cells in Rhesus Macaques. Cytometry (1999) 37:41–50. doi: 10.1002/(sici)1097-0320(19990901)37:1<41::aid-cyto5>3.0.co;2-4 10451505

[17] Fragiadakis GK Bjornson ZB Madhireddy D Sachs K Chen H McIlwain DR. Variation of Immune Cell Responses in Humans Reveals Sex-Specific Coordinated Signaling Across Cell Types. Front Immunol (2022) doi: 10.3389/fimmu.2022.867016 PMC8995898 35419006

[18] Autissier P Soulas C Burdo TH Williams KC. Immunophenotyping of Lymphocyte, Monocyte and Dendritic Cell Subsets in Normal Rhesus Macaques by 12-Color Flow Cytometry: Clarification on DC Heterogeneity. J Immunol Methods (2010) 360:119–28. doi: 10.1016/j.jim.2010.06.017 PMC2930593 20600075

[19] Autissier P Soulas C Burdo TH Williams KC. Evaluation of a 12-Color Flow Cytometry Panel to Study Lymphocyte, Monocyte, and Dendritic Cell Subsets in Humans. Cytom Part A (2010) 77A:410–9. doi: 10.1002/cyto.a.20859 PMC11742174 20099249

[20] Coates PTH Barratt-Boyes SM Zhang L Donnenberg VS O’Connell PJ Logar AJ. Dendritic Cell Subsets in Blood and Lymphoid Tissue of Rhesus Monkeys and Their Mobilization With Flt3 Ligand. Blood (2003) 102:2513–21. doi: 10.1182/blood-2002-09-2929 12829599

[21] Jesudason S Collins MG Rogers NM Kireta S Coates PTH. Non-Human Primate Dendritic Cells. J Leukocyte Biol (2012) 91:217–28. doi: 10.1189/jlb.0711355 22124138

[22] Reimann KA Waite BCD Lee-Parritz DE Lin W Uchanska-Ziegler B O’Connell MJ. Use of Human Leukocyte-Specific Monoclonal Antibodies for Clinically Immunophenotyping Lymphocytes of Rhesus Monkeys. Cytometry (1994) 17:102–8. doi: 10.1002/cyto.990170113 8001455

[23] Sopper S Stahl-Hennig C Demuth M Johnston ICD Dörries R ter Meulen V. Lymphocyte Subsets and Expression of Differentiation Markers in Blood and Lymphoid Organs of Rhesus Monkeys. Cytometry (1997) 29:351–62. doi: 10.1002/(sici)1097-0320(19971201)29:4<351::aid-cyto12>3.0.co;2-t 9415418

[24] Webster RL Johnson RP. Delineation of Multiple Subpopulations of Natural Killer Cells in Rhesus Macaques. Immunology (2005) 115:206–14. doi: 10.1111/j.1365-2567.2005.02147.x PMC1782152 15885126

[25] Brooke G Holbrook JD Brown MH Barclay AN. Human Lymphocytes Interact Directly With CD47 Through a Novel Member of the Signal Regulatory Protein (SIRP) Family. J Immunol (2004) 173:2562–70. doi: 10.4049/jimmunol.173.4.2562 15294972

[26] Barclay AN Brown MH. The SIRP Family of Receptors and Immune Regulation. Nat Rev Immunol (2006) 6:457–64. doi: 10.1038/nri1859 16691243

[27] Piccio L Vermi W Boles KS Fuchs A Strader CA Facchetti F. Adhesion of Human T Cells to Antigen-Presenting Cells Through Sirpβ2-CD47 Interaction Costimulates T-Cell Proliferation. Blood (2005) 105:2421–7. doi: 10.1182/blood-2004-07-2823 15383453

[28] Yagita H Nakamura T Asakawa J-I Matsuda H Tansyo S Yutakaligo. CD2 Expression in Murine B Cell Lineage. Int Immunol (1989) 1:94–8. doi: 10.1093/intimm/1.1.94 2577288

[29] Davis SJ van der Merwe PA. The Structure and Ligand Interactions of CD2: Implications for T-Cell Function. Immunol Today (1996) 17:177–87. doi: 10.1016/0167-5699(96)80617-7 8871350

[30] Kingma DW Imus P Xie XY Jasper G Sorbara L Stewart C. CD2 is Expressed by a Subpopulation of Normal B Cells and is Frequently Present in Mature B-Cell Neoplasms. Cytometry (2002) 50:243–8. doi: 10.1002/cyto.10131 12360573

[31] Davis SJ Ikemizu S Wild MK Merwe PA. CD2 and the Nature of Protein Interactions Mediating Cell-Cell Recognition. Immunol Rev (1998) 163:217–36. doi: 10.1111/j.1600-065x.1998.tb01199.x 9700513

[32] Sadhu C Hendrickson L Dick KO Potter TG Staunton DE. Novel Tools for Functional Analysis of CD11c: Activation-Specific, Activation-Independent, and Activating Antibodies. J Immunoass Immunochem (2007) 29:42–57. doi: 10.1080/15321810701735062 18080879

[33] Ihanus E Uotila LM Toivanen A Varis M Gahmberg CG. Red-Cell ICAM-4 is a Ligand for the Monocyte/Macrophage Integrin CD11c/CD18: Characterization of the Binding Sites on ICAM-4. Blood (2006) 109:802–10. doi: 10.1182/blood-2006-04-014878 16985175

[34] Valentine M Song K Maresh GA Mack H Huaman MC Polacino P. Expression of the Memory Marker CD45RO on Helper T Cells in Macaques. PloS One (2013) 8:e73969. doi: 10.1371/journal.pone.0073969 24023920 PMC3762710

[35] Pitcher CJ Hagen SI Walker JM Lum R Mitchell BL Maino VC. Development and Homeostasis of T Cell Memory in Rhesus Macaque. J Immunol (2002) 168:29–43. doi: 10.4049/jimmunol.168.1.29 11751943

[36] Glazko GV Nei M. Estimation of Divergence Times for Major Lineages of Primate Species. Mol Biol Evol (2003) 20:424–34. doi: 10.1093/molbev/msg050 12644563

[37] Pozzi L Hodgson JA Burrell AS Sterner KN Raaum RL Disotell TR. Primate Phylogenetic Relationships and Divergence Dates Inferred From Complete Mitochondrial Genomes. Mol Phylogenet Evol (2014) 75:165–83. doi: 10.1016/j.ympev.2014.02.023 PMC4059600 24583291

[38] Perelman P Johnson WE Roos C Seuánez HN Horvath JE Moreira MAM. A Molecular Phylogeny of Living Primates. PloS Genet (2011) 7:e1001342. doi: 10.1371/journal.pgen.1001342 21436896 PMC3060065

[39] Campbell JP Guy K Cosgrove C Florida-James GD Simpson RJ. Total Lymphocyte CD8 Expression is Not a Reliable Marker of Cytotoxic T-Cell Populations in Human Peripheral Blood Following an Acute Bout of High-Intensity Exercise. Brain Behav Immun (2008) 22:375–80. doi: 10.1016/j.bbi.2007.09.001 17949944

[40] Liu J Xu C Hsu L-C Luo Y Xiang R Chuang T-H. A Five-Amino-Acid Motif in the Undefined Region of the TLR8 Ectodomain is Required for Species-Specific Ligand Recognition. Mol Immunol (2010) 47:1083–90. doi: 10.1016/j.molimm.2009.11.003 PMC2815190 20004021

[41] Guiducci C Gong M Cepika A-M Xu Z Tripodo C Bennett L. RNA Recognition by Human TLR8 can Lead to Autoimmune Inflammation. J Exp Med (2013) 210:2903–19. doi: 10.1084/jem.20131044 PMC3865472 24277153

[42] Dunzendorfer S Lee H-K Soldau K Tobias PS. TLR4 Is the Signaling But Not the Lipopolysaccharide Uptake Receptor. J Immunol (2004) 173:1166–70. doi: 10.4049/jimmunol.173.2.1166 15240706

[43] Vaure C Liu Y. A Comparative Review of Toll-Like Receptor 4 Expression and Functionality in Different Animal Species. Front Immunol (2014) 5:316. doi: 10.3389/fimmu.2014.00316 25071777 PMC4090903

[44] León B del Hoyo GM Parrillas V Vargas HH Sánchez-Mateos P Longo N. Dendritic Cell Differentiation Potential of Mouse Monocytes: Monocytes Represent Immediate Precursors of CD8- and CD8+ Splenic Dendritic Cells. Blood (2004) 103:2668–76. doi: 10.1182/blood-2003-01-0286 14630812

[45] Rose S Misharin A Perlman H. A Novel Ly6C/Ly6G-Based Strategy to Analyze the Mouse Splenic Myeloid Compartment. Cytom Part A (2012) 81A:343–50. doi: 10.1002/cyto.a.22012 PMC3987771 22213571

[46] Kaplan MH Schindler U Smiley ST Grusby MJ. Stat6 Is Required for Mediating Responses to IL-4 and for the Development of Th2 Cells. Immunity (1996) 4:313–9. doi: 10.1016/s1074-7613(00)80439-2 8624821

[47] Takeda K Tanaka T Shi W Matsumoto M Minami M Kashiwamura S. Essential Role of Stat6 in IL-4 Signalling. Nature (1996) 380:627–30. doi: 10.1038/380627a0 8602263

[48] Tormo AJ Letellier M-C Sharma M Elson G Crabé S Gauchat J-F. IL-6 Activates STAT5 in T Cells. Cytokine (2012) 60:575–82. doi: 10.1016/j.cyto.2012.07.002 22854263

[49] Mayer-Scholl A Hurwitz R Brinkmann V Schmid M Jungblut P Weinrauch Y. Human Neutrophils Kill Bacillus Anthracis. PloS Pathog (2005) 1:e23. doi: 10.1371/journal.ppat.0010023 16292357 PMC1283252

[50] Tran D Tran P Roberts K Ösapay G Schaal J Ouellette A. Microbicidal Properties and Cytocidal Selectivity of Rhesus Macaque Theta Defensins▿. Antimicrob Agents Ch (2008) 52:944–53. doi: 10.1128/aac.01090-07 PMC2258523 18160518

[51] Wang W Mulakala C Ward SC Jung G Luong H Pham D. Retrocyclins Kill Bacilli and Germinating Spores of Bacillus Anthracis and Inactivate Anthrax Lethal Toxin*. J Biol Chem (2006) 281:32755–64. doi: 10.1074/jbc.m603614200 PMC2440672 16790431

[52] Kim C Gajendran N Mittrücker H-W Weiwad M Song Y-H Hurwitz R. Human α-Defensins Neutralize Anthrax Lethal Toxin and Protect Against its Fatal Consequences. P Natl Acad Sci USA (2005) 102:4830–5. doi: 10.1073/pnas.0500508102 PMC555714 15772169

[53] Rhijn IV Moody DB. CD1 and Mycobacterial Lipids Activate Human T Cells. Immunol Rev (2015) 264:138–53. doi: 10.1111/imr.12253 PMC4339259 25703557

[54] Chackerian AA Behar SM. Susceptibility to Mycobacterium Tuberculosis: Lessons From Inbred Strains of Mice. Tuberculosis (2003) 83:279–85. doi: 10.1016/s1472-9792(03)00017-9 12972341

[55] Schlesinger M Rabinowitz R Levy P Maayan S. The Expression of CD8 on B Lymphocytes in HIV-Infected Individuals. Immunol Lett (1996) 50:23–7. doi: 10.1016/0165-2478(96)02510-2 8793555

[56] Islam A Vladutiu AO Donahue T Akhter S Sands AM Ambrus JL. CD8 Expression on B Cells in Chronic Lymphocytic Leukemia. Arch Pathol Lab Med (2000) 124:1361–3. doi: 10.5858/2000-124-1361-ceobci 10975939

[57] Koelliker DD Steele PE Hurtubise PE Flessa HC Sheng Y-HP Swerdlow SH. CD8-Positive B-Cell Chronic Lymphocytic Leukemia: A Report of Two Cases. Am J Clin Pathol (1994) 102:212–6. doi: 10.1093/ajcp/102.2.212 8042591

[58] Mulligan SP Dao LP Francis SE Thomas ME Gibson J Cole-Sinclair MF. B-Cell Chronic Lymphocytic Leukaemia With CD8 Expression: Report of 10 Cases and Immunochemical Analysis of the CD8 Antigen. Brit J Haematol (1998) 103:157–62. doi: 10.1046/j.1365-2141.1998.00928.x 9792303

[59] Carulli G Stacchini A Marini A Ciriello MM Zucca A Cannizzo E. Aberrant Expression of CD8 in B-Cell Non-Hodgkin LymphomaA Multicenter Study of 951 Bone Marrow Samples With Lymphomatous Infiltration. Am J Clin Pathol (2009) 132:186–90. doi: 10.1309/ajcpncohs92arwrq 19605812

[60] Nurden AT George JN Phillips DR. Platelet Membrane Glycoproteins: Their Structure, Function, and Modification in Disease. In: Inc AP, editor. Biochemistry of Platelets. New York, NY, USA: Academic Press Inc (1986). p. 160–212.

[61] Jasinska AJ Schmitt CA Service SK Cantor RM Dewar K Jentsch JD. Systems Biology of the Vervet Monkey. Ilar J (2013) 54:122–43. doi: 10.1093/ilar/ilt049 PMC3814400 24174437

[62] Cohen J. Vaccine Studies Stymied by Shortage of Animals. Science (2000) 287(5455):959–60. doi: 10.1126/science.287.5455.959 10691568

[63] Brezinschek RI Oppenheimer-Marks N Lipsky PE. Activated T Cells Acquire Endothelial Cell Surface Determinants During Transendothelial Migration. J Immunol Baltim Md 1950 (1999) 162:1677–84. 9973429

[64] Coleman WG Goebel PJ Leive L. Genetic analysis of Escherichia coli O111:B4, a strain of medical and biochemical interest. J Bacteriol (1977) 130:656–60. doi: 10.1128/jb.130.2.656-660.1977 PMC235264 400785

[65] Johnson RF Bell P Harty RN. Effect of Ebola Virus Proteins GP, NP and VP35 on VP40 VLP Morphology. Virol J (2006) 3:31. doi: 10.1186/1743-422x-3-31 16719918 PMC1502131

[66] Licata JM Johnson RF Han Z Harty RN. Contribution of Ebola Virus Glycoprotein, Nucleoprotein, and VP24 to Budding of VP40 Virus-Like Particles. J Virol (2004) 78:7344–51. doi: 10.1128/jvi.78.14.7344-7351.2004 PMC434112 15220407

[67] Sun Y Carrion R Ye L Wen Z Ro Y-T Brasky K. Protection Against Lethal Challenge by Ebola Virus-Like Particles Produced in Insect Cells. Virology (2009) 383:12–21. doi: 10.1016/j.virol.2008.09.020 18986663 PMC2657000

[68] Warfield KL Swenson DL Olinger GG Kalina WV Aman MJ Bavari S. Ebola Virus-Like Particle-Based Vaccine Protects Nonhuman Primates Against Lethal Ebola Virus Challenge. J Infect Dis (2007) 196:S430–7. doi: 10.1086/520583 17940980

[69] Warfield KL Posten NA Swenson DL Olinger GG Esposito D Gillette WK. Filovirus-Like Particles Produced in Insect Cells: Immunogenicity and Protection in Rodents. J Infect Dis (2007) 196:S421–9. doi: 10.1086/520612 17940979

[70] Ayithan N Bradfute SB Anthony SM Stuthman KS Dye JM Bavari S. Ebola Virus-Like Particles Stimulate Type I Interferons and Proinflammatory Cytokine Expression Through the Toll-Like Receptor and Interferon Signaling Pathways. J Interf Cytokine Res (2014) 34:79–89. doi: 10.1089/jir.2013.0035 PMC3924795 24102579

[71] Chow S Hedley D Grom P Magari R Jacobberger JW Shankey TV. Whole Blood Fixation and Permeabilization Protocol With Red Blood Cell Lysis for Flow Cytometry of Intracellular Phosphorylated Epitopes in Leukocyte Subpopulations. Cytom Part A (2005) 67A:4–17. doi: 10.1002/cyto.a.20167 16080188

[72] Behbehani GK. Applications of Mass Cytometry in Clinical Medicine The Promise and Perils of Clinical CyTOF. Clin Lab Med (2017) 37:945–64. doi: 10.1016/j.cll.2017.07.010 29128078

[73] Zunder ER Finck R Behbehani GK Amir ED Krishnaswamy S Gonzalez VD. Palladium-Based Mass Tag Cell Barcoding With a Doublet-Filtering Scheme and Single-Cell Deconvolution Algorithm. Nat Protoc (2015) 10:316–33. doi: 10.1038/nprot.2015.020 PMC4347881 25612231

[74] Finck R Simonds EF Jager A Krishnaswamy S Sachs K Fantl W. Normalization of Mass Cytometry Data With Bead Standards. Cytom Part A (2013) 83A:483–94. doi: 10.1002/cyto.a.22271 PMC3688049 23512433

[75] Procedures C for CR of DL. Review Committee Report: Inadvertent Shipment of Live Bacillus Anthracis Spores by DoD (Department of Defense) (2015). Available at: https://dod.defense.gov/Portals/1/features/2015/0615_lab-stats/Review-Committee-Report-Final.pdf (Accessed December 15, 2021).

