# Supplementary figures and images for: A Comprehensive Atlas of Immunological Differences Between Humans, Mice, and Non-Human Primates
Source: Front Immunol. 2022 Mar 11;13:867015. doi: 10.3389/fimmu.2022.867015 (PMC8962947; doi:10.3389/fimmu.2022.867015)

Supplementary Figure 1.

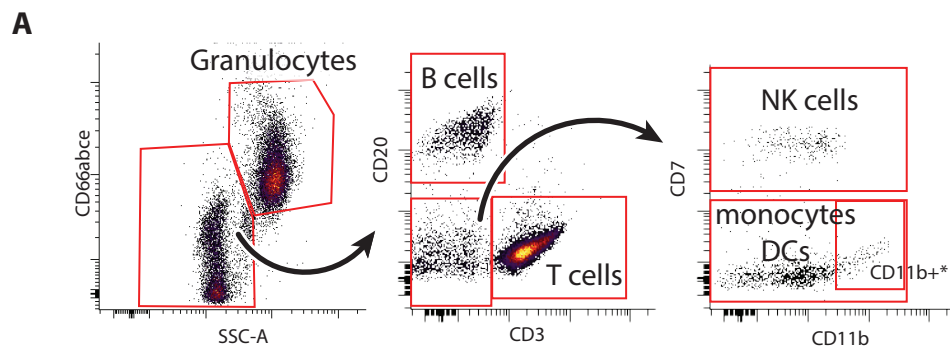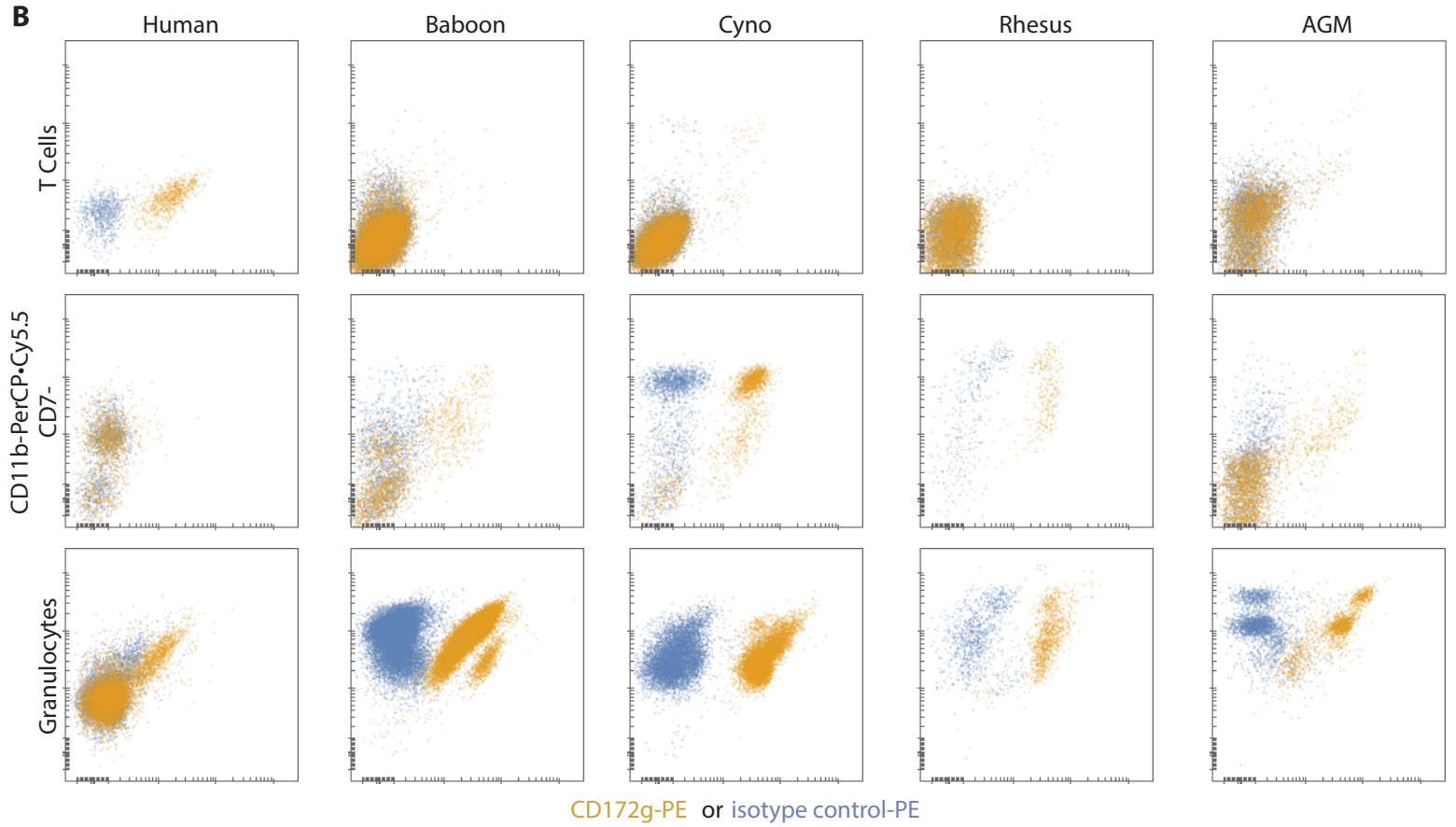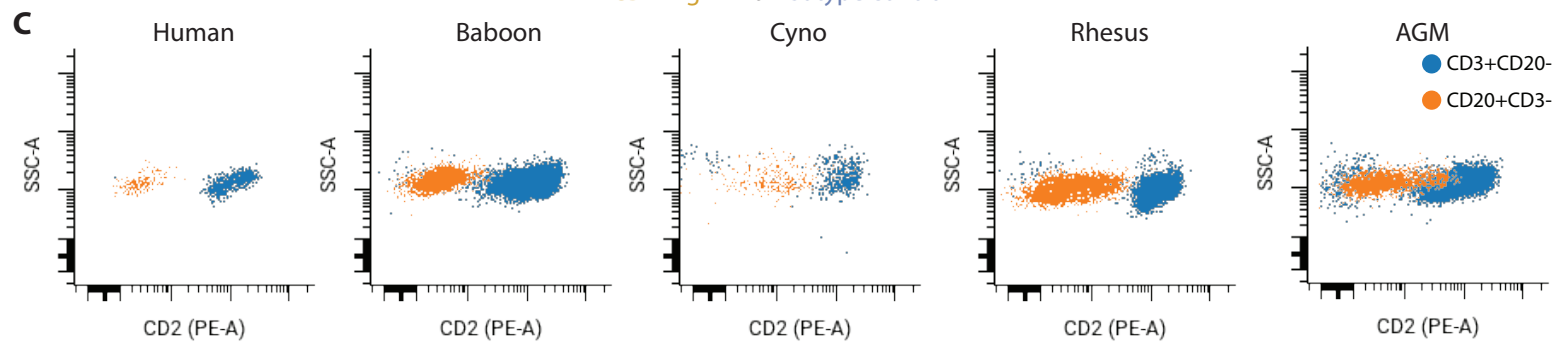

Supplement: Supplementary Figure 1 — (A) Five counterstains allow identification of at least five cell populations. Representative staining from a baboon shown. After gating by time to exclude artifacts, granulocytes were identified as CD66abce+/SSC-A+. Non-granulocytes were divided into B cells (CD20+/CD3−), T cells (CD3+/CD20−), NK cells (CD7+/CD3−/CD20−) and monocytes/dendritic cells (CD7−/CD3−/CD20−). *In species other than the African green monkey, dendritic cells could be separated on the basis of CD11b staining. (B) CD172g is expressed on monocytes and all granulocytes, but not on T cells, in examined NHP species. By comparison, CD172g is expressed on all T cells, a subset of granulocytes and a subset of B cells (data not shown) in humans. As discussed in the text, CD11b did not uniformly stain AGMs; thus, some of the CD11b-negative cells are monocytes. Blue: isotype control, orange: CD172g. (C) CD2 is expressed on B cells in NHPs. Blue: CD3+ T cells where expression is expected in humans; orange: CD20+ B cells. [file DataSheet_1.pdf]

Ungated

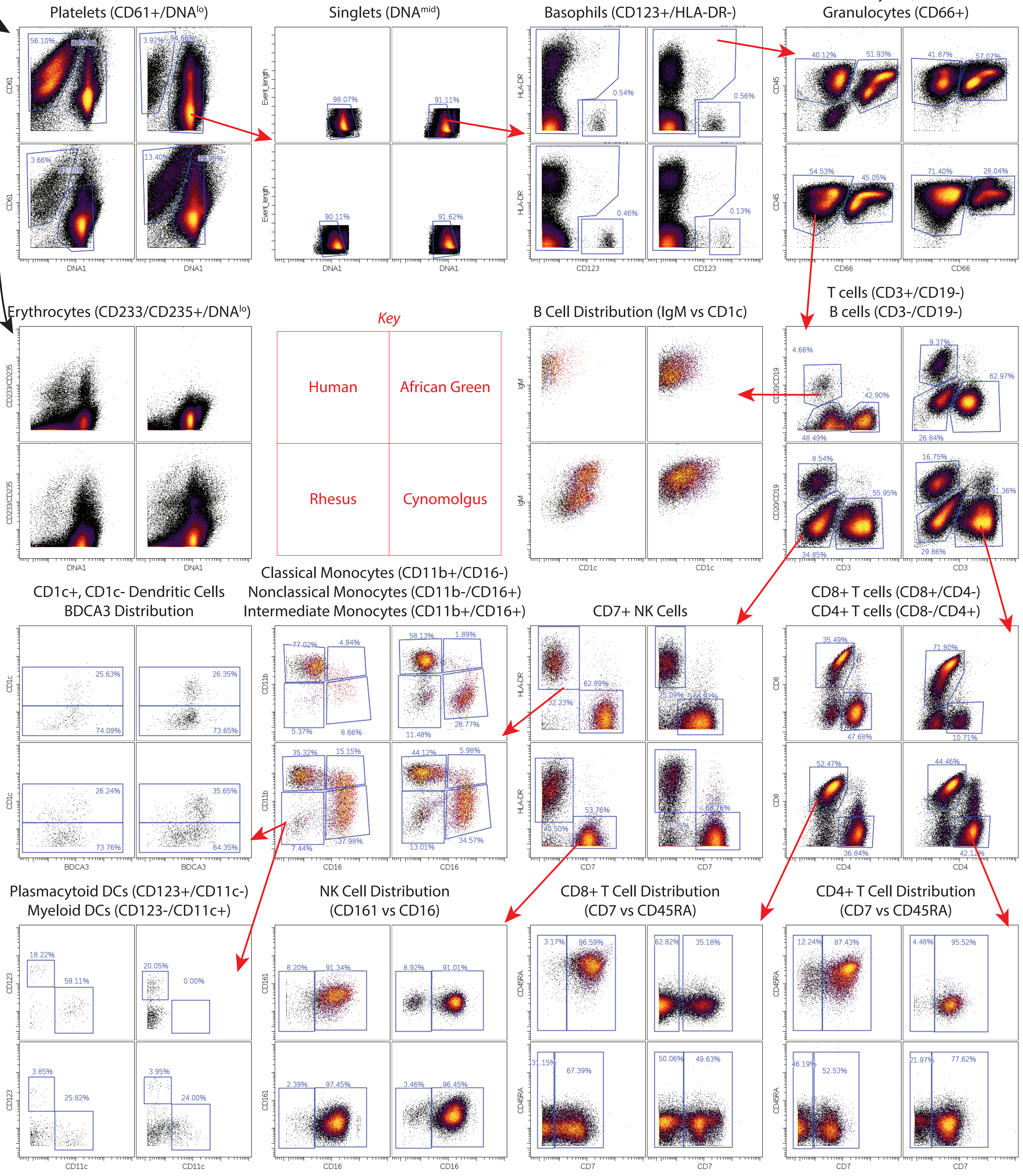

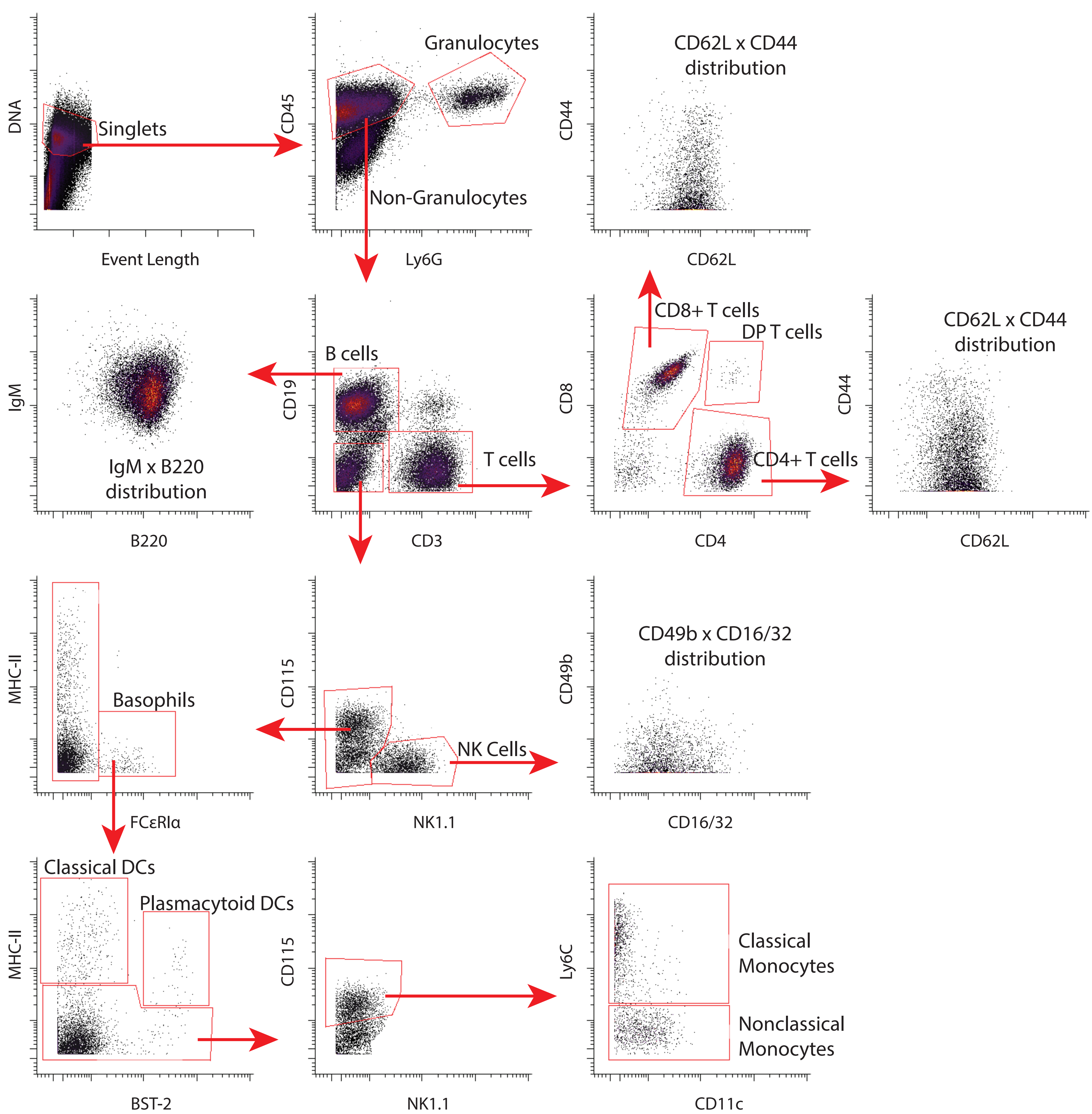

Supplement: Supplementary Figure 2 — Universal phenotyping panels enable parallel gating in humans, macaques, African green monkeys and mice. [file DataSheet_2.pdf]

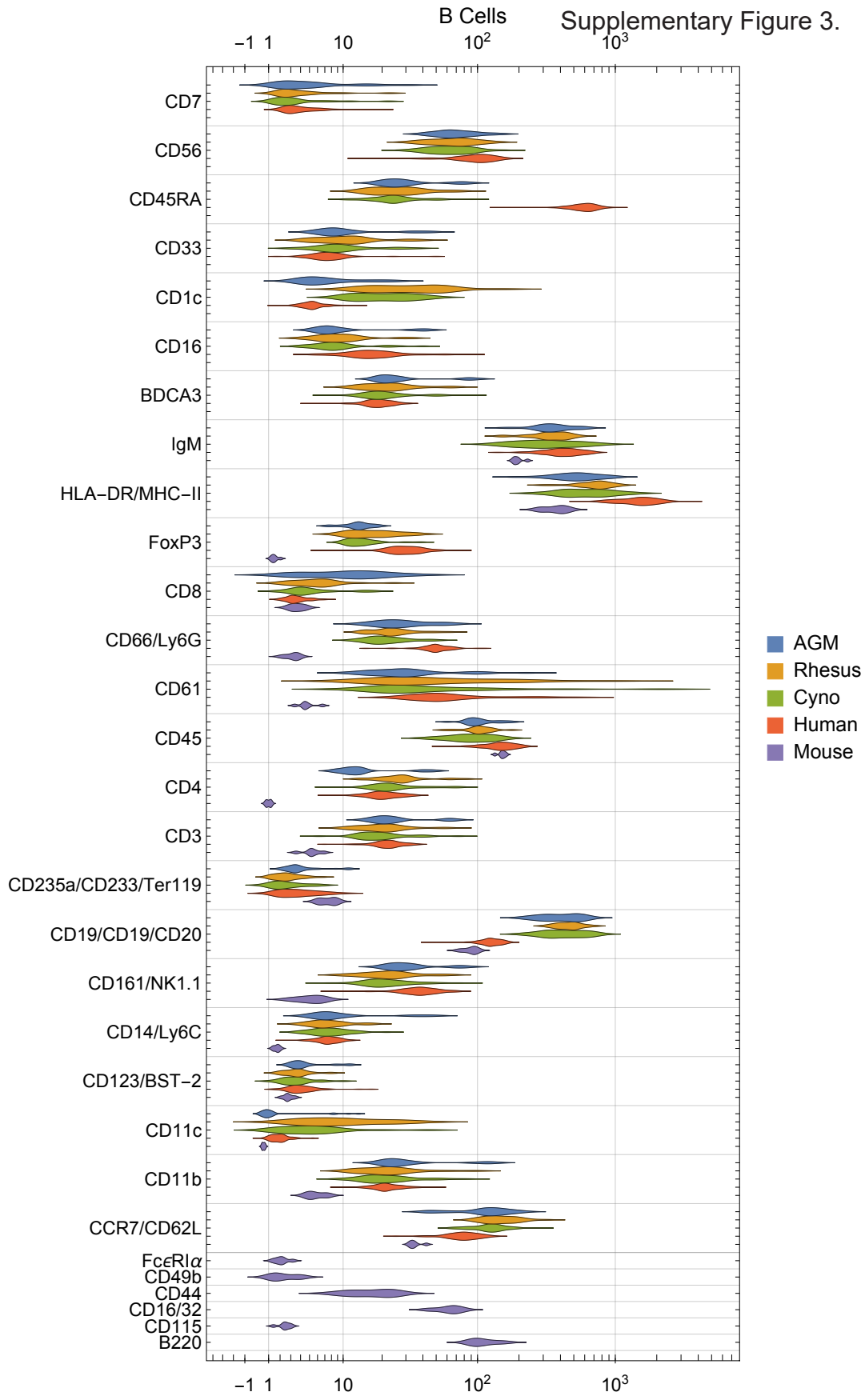

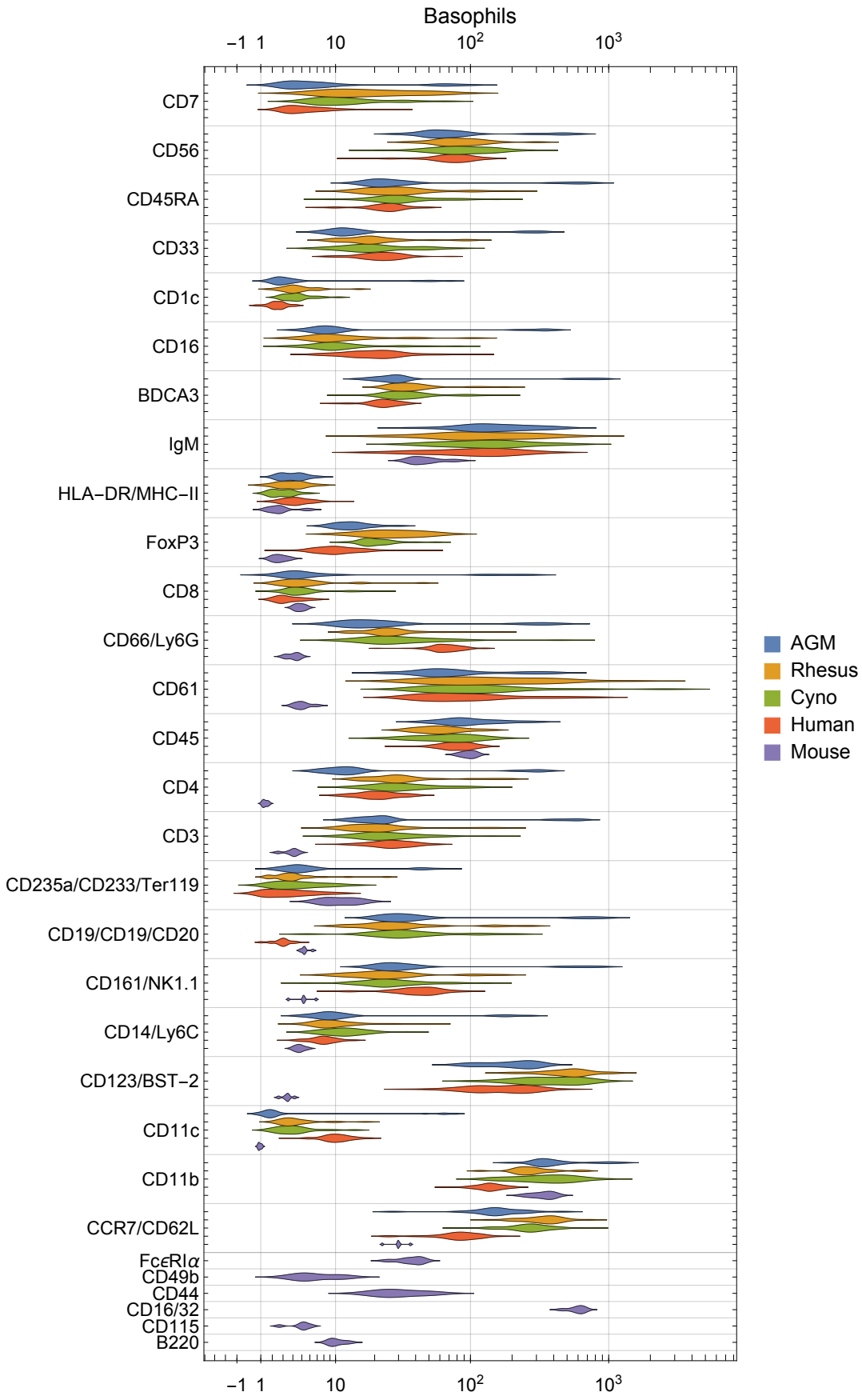

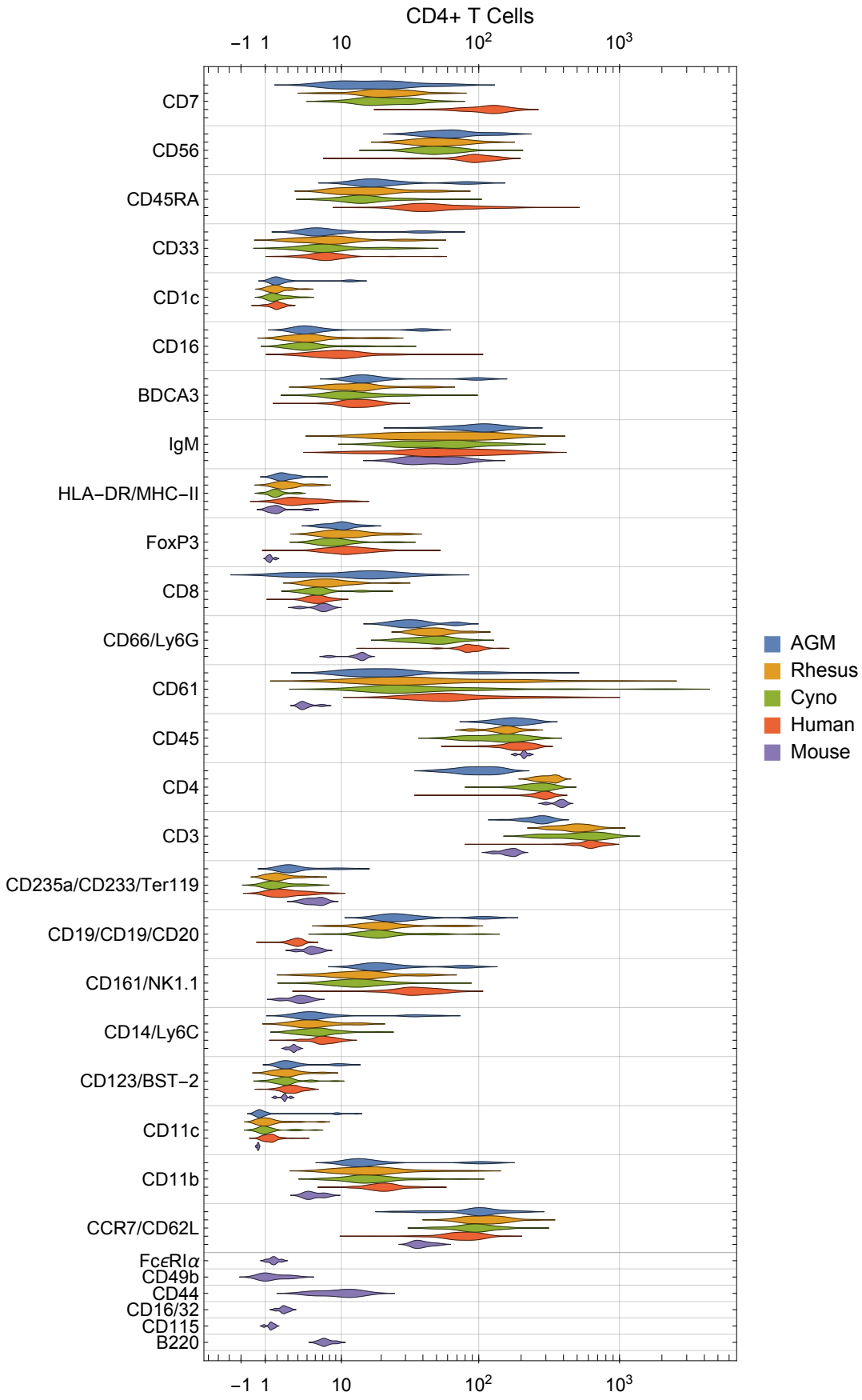

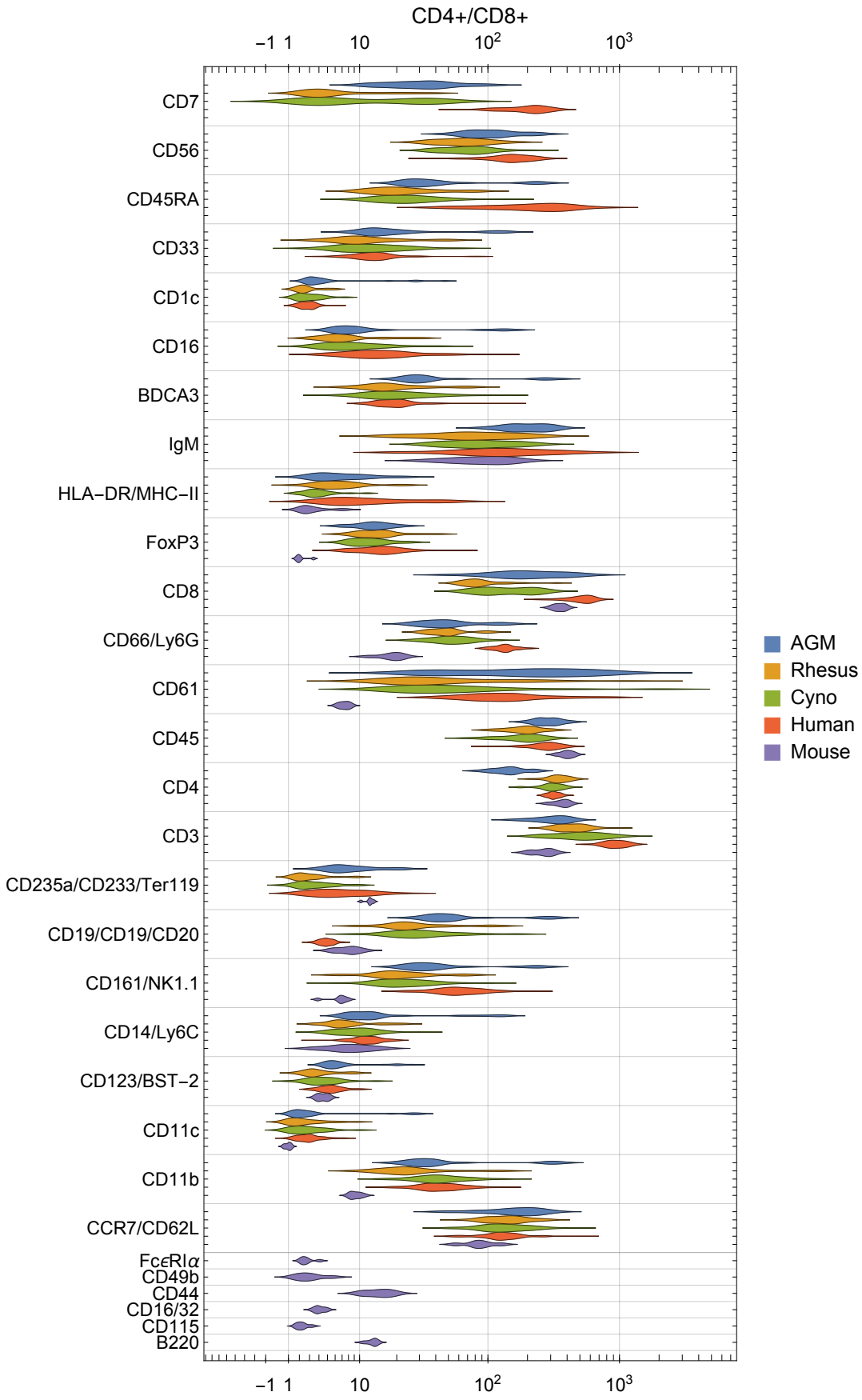

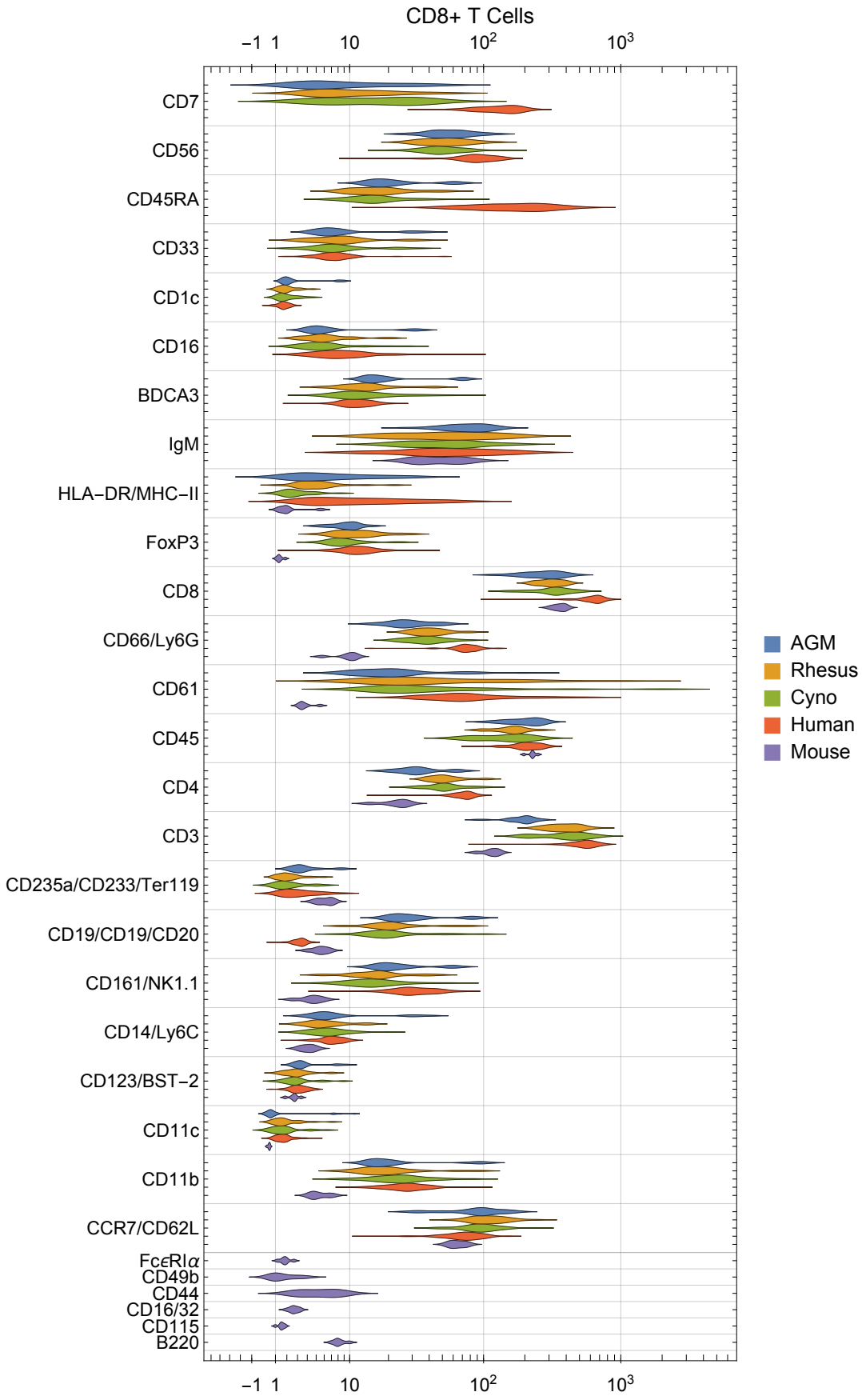

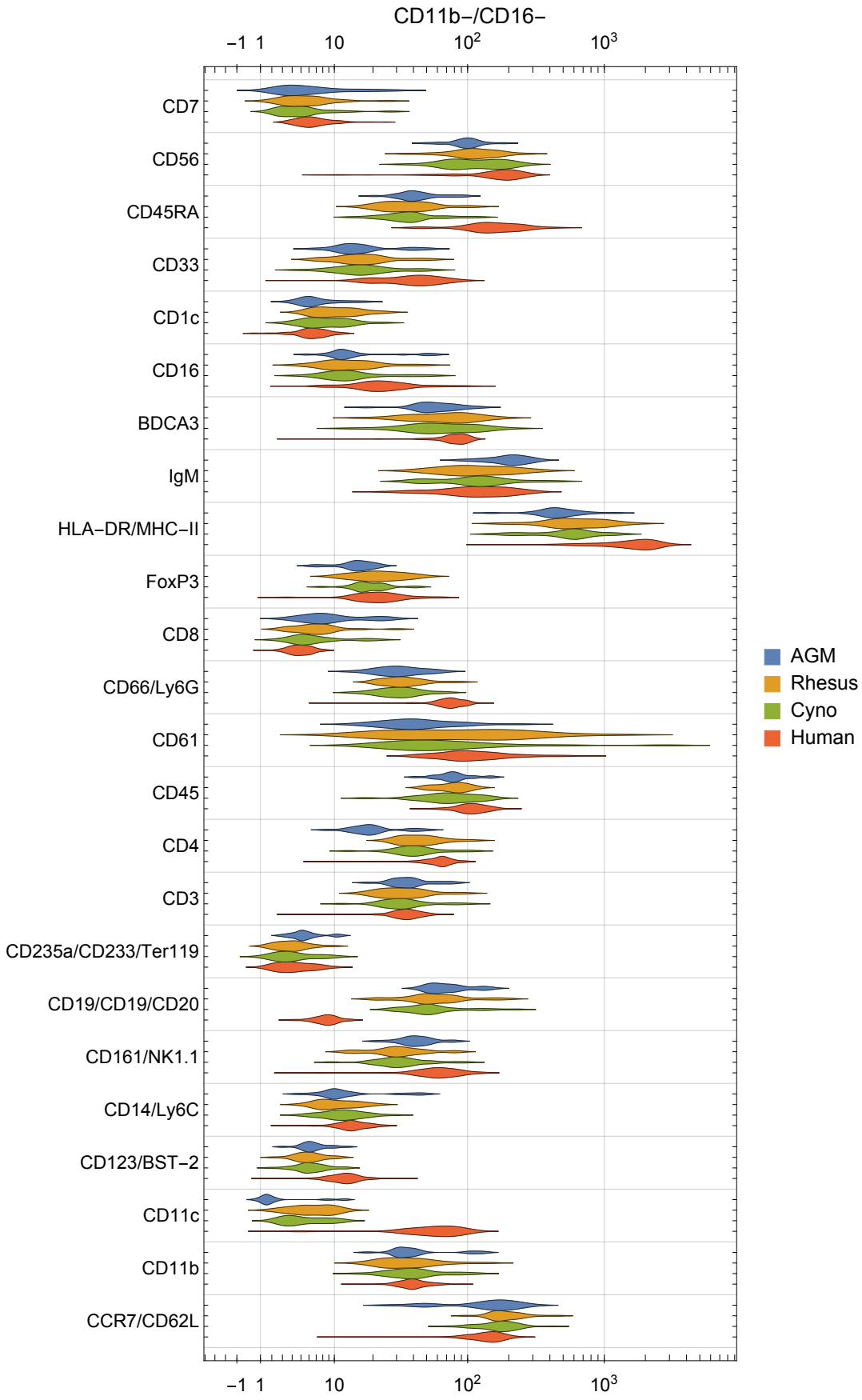

# Classical Monocytes

-1 1 10 10<sup>2</sup> 10<sup>3</sup>

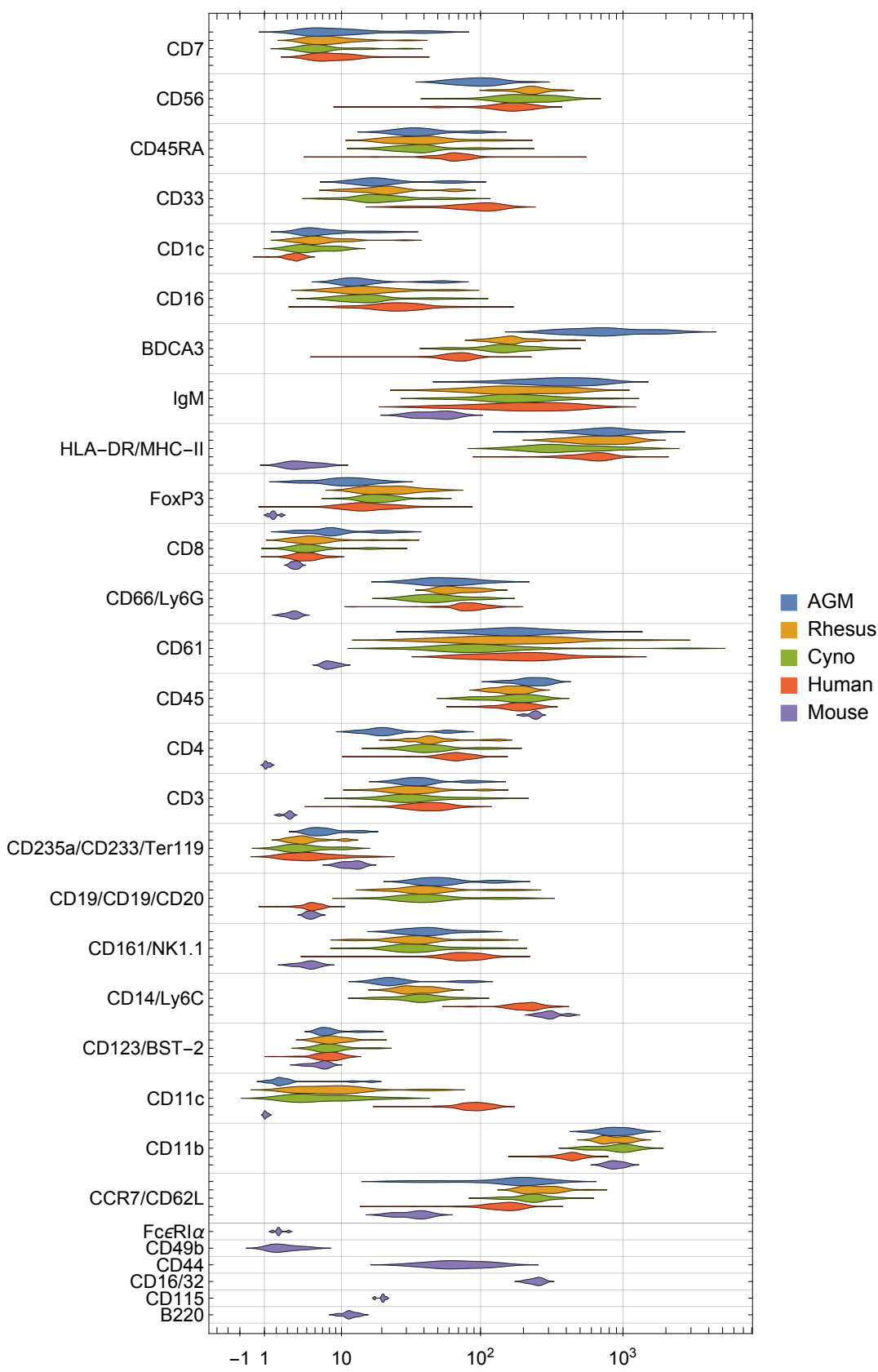

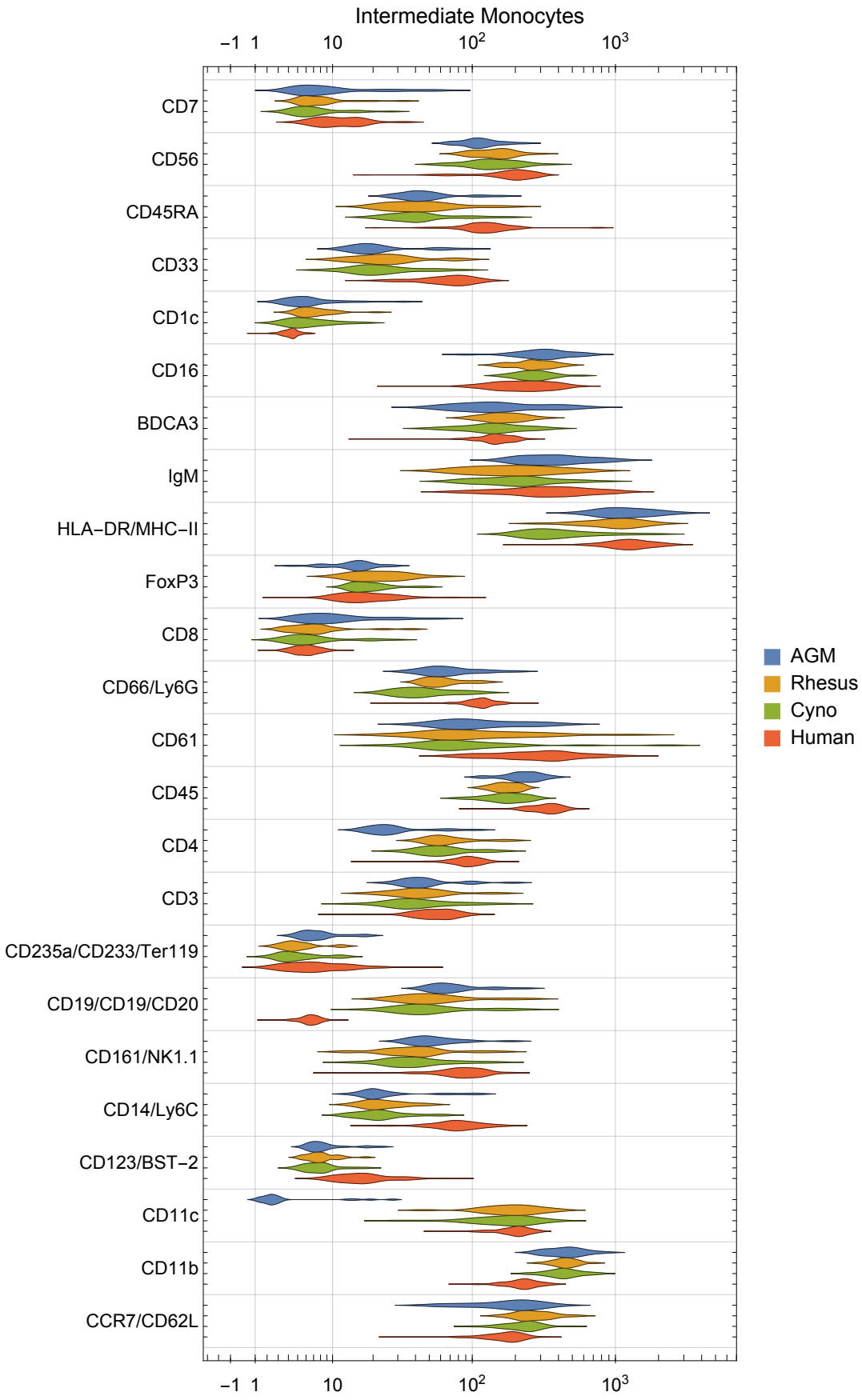

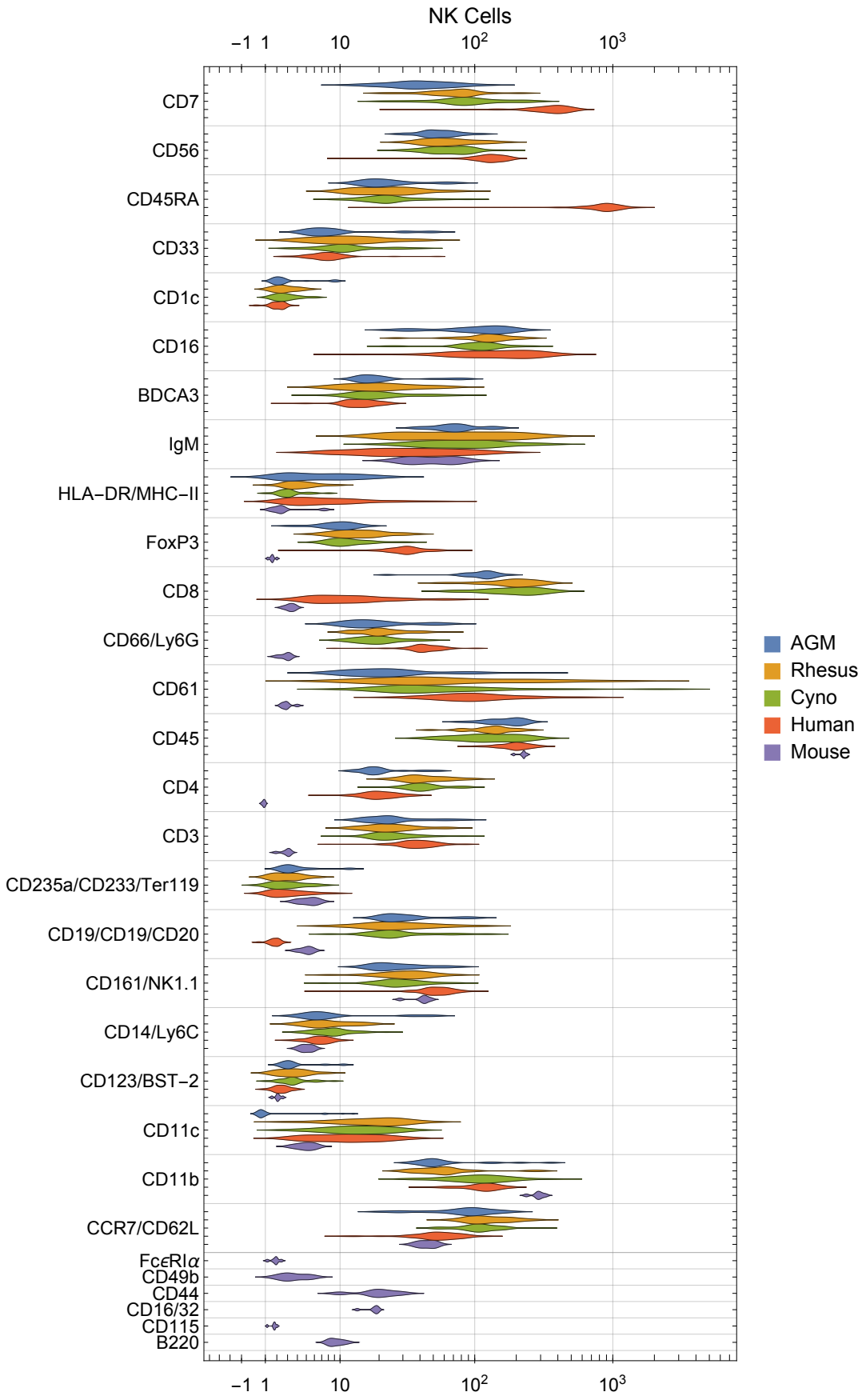

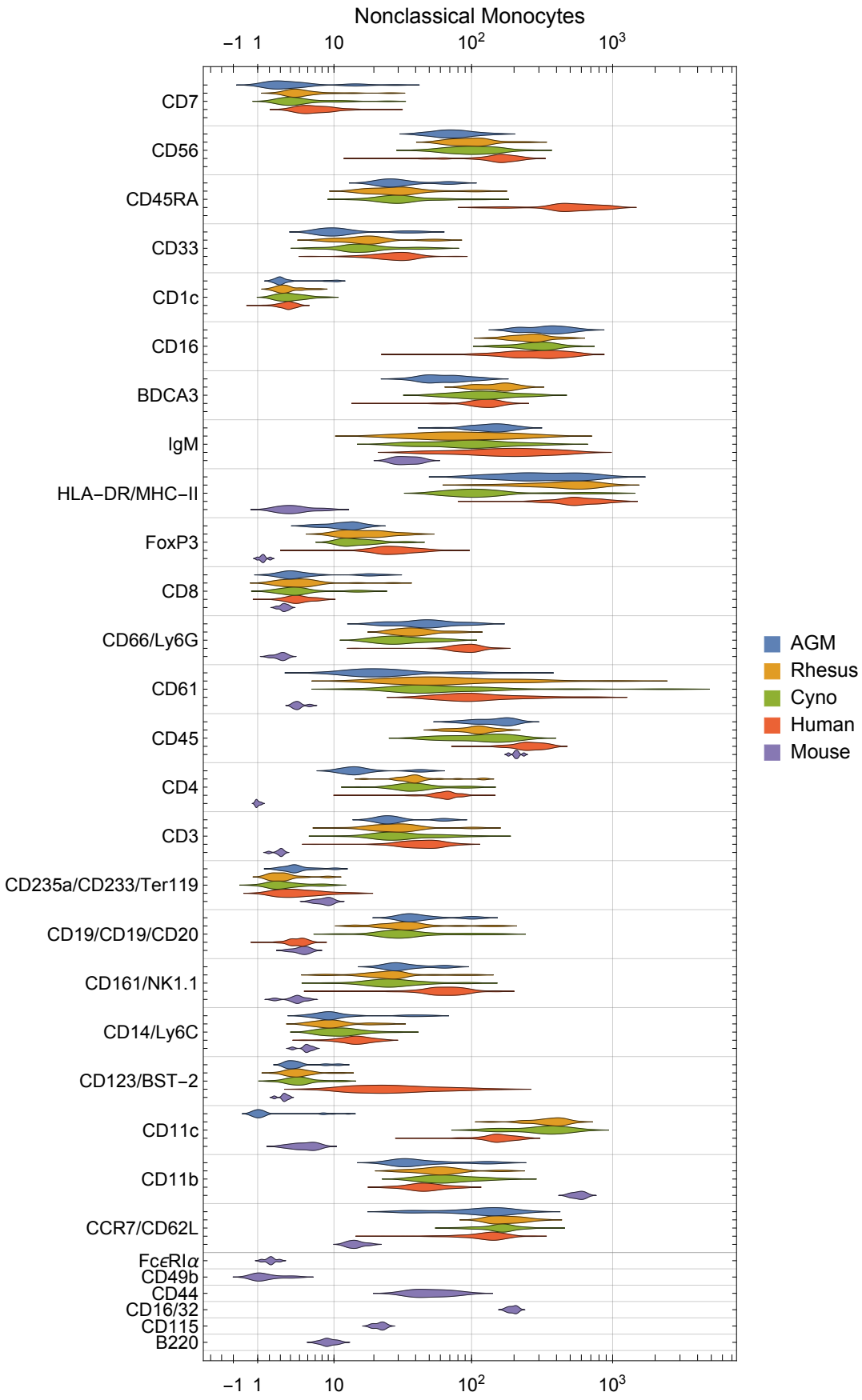

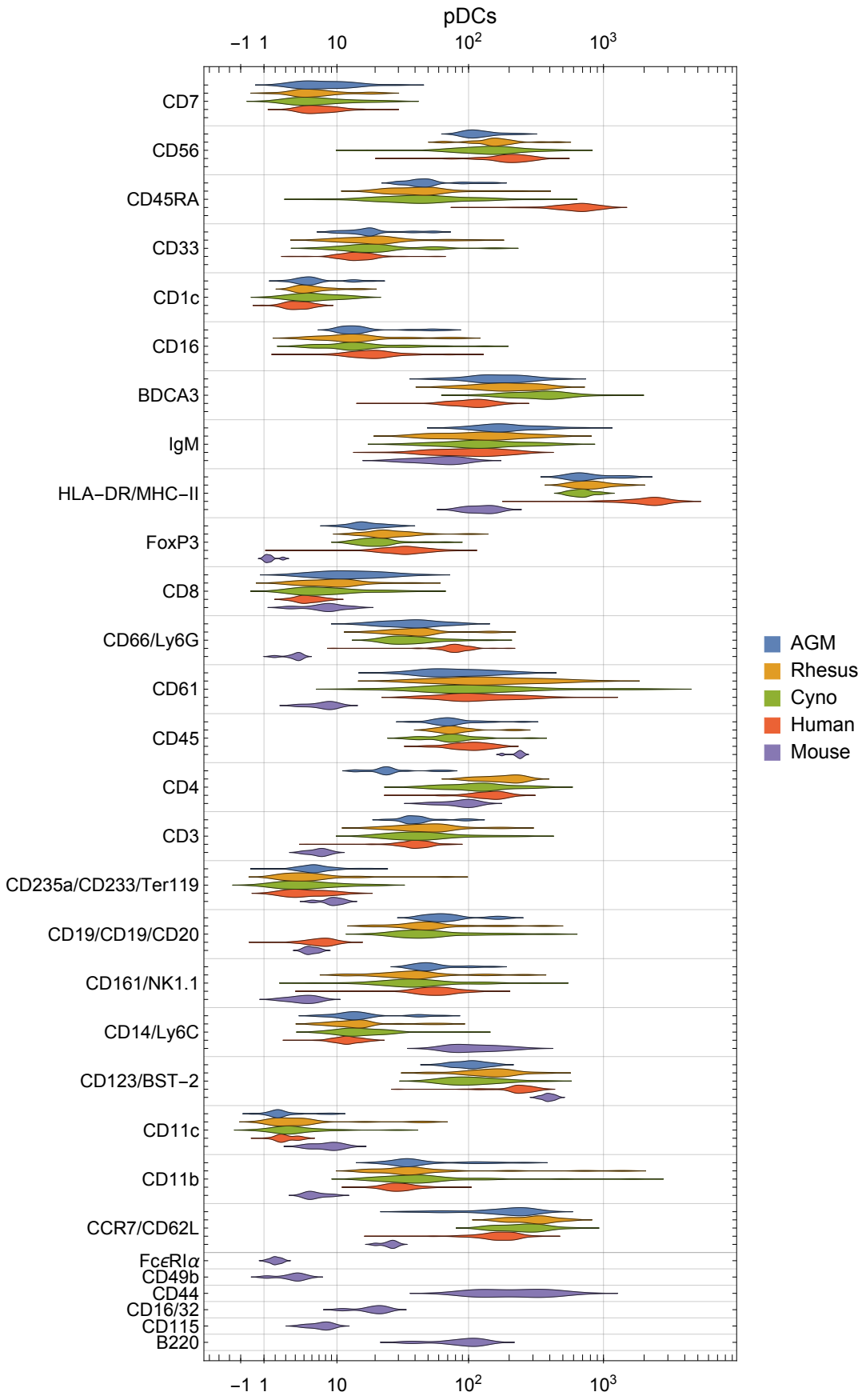

Supplement: Supplementary Figure 3 — Continuation of Figure 4 : Distribution of surface marker expression for each species in all cell types. [file DataSheet_3.pdf]

Supplementary Figure 5.

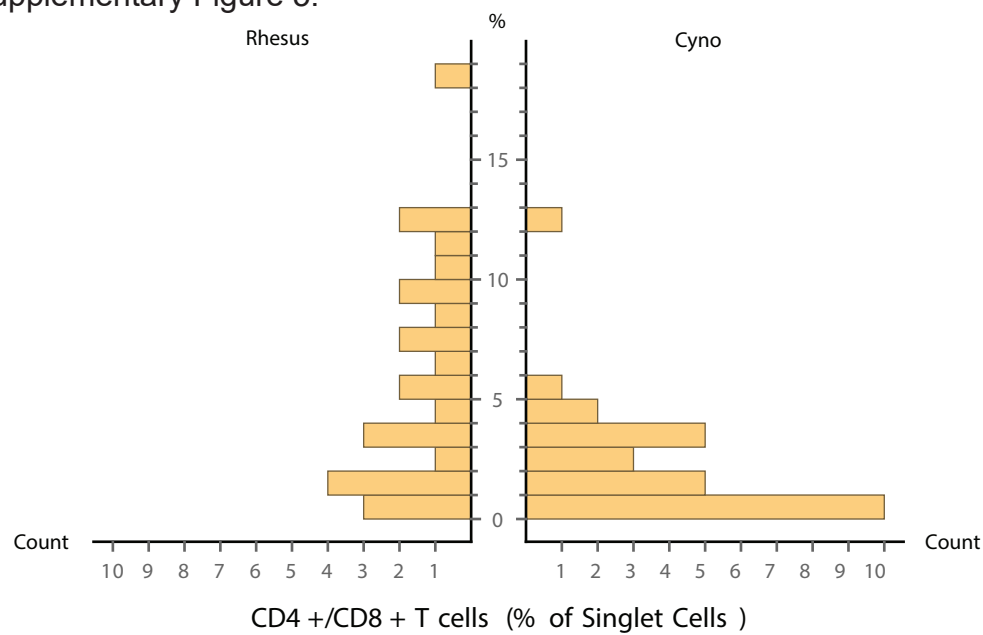

| Species | Percent  |
|---------|----------|
| Human   | 0.110735 |
| Rhesus  | 5.26584  |
| Cyno    | 1.42308  |
| AGM     | 0.111882 |
| Mouse   | 0.183403 |

Supplement: Supplementary Figure 5 — Frequency distribution of CD4+CD8+ double positive T cells for rhesus and cynomolgus macaques. Table: Median frequencies (percent of singlet cells) by species. [file DataSheet_5.pdf]

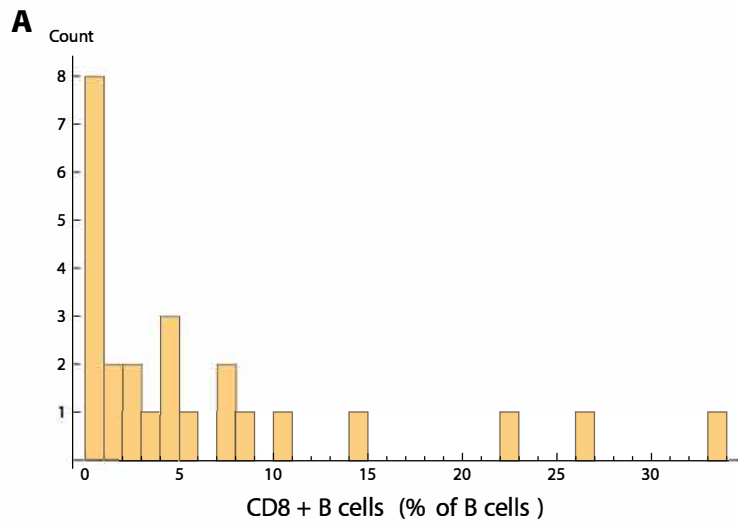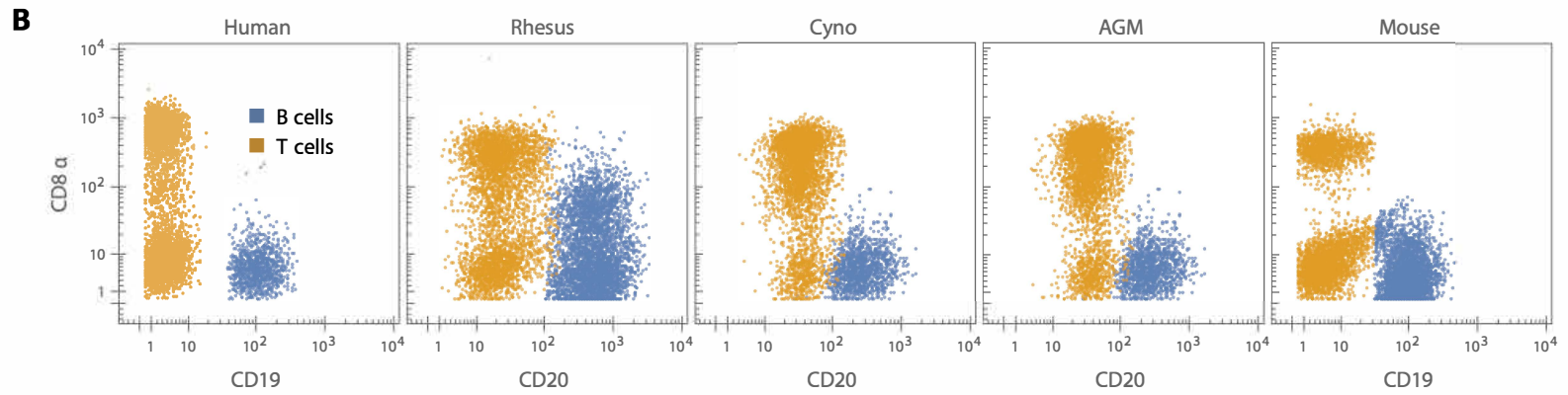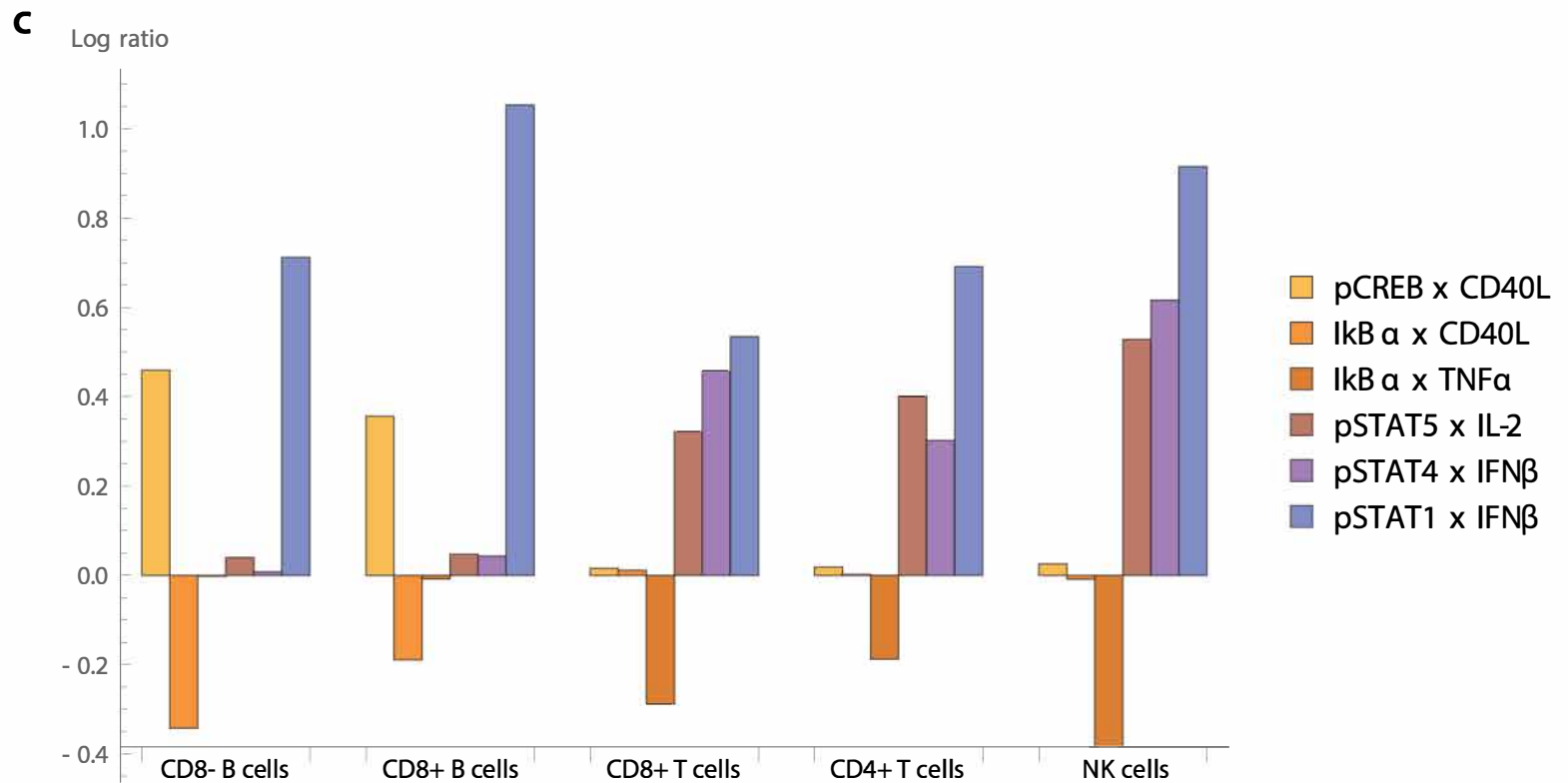

Supplement: Supplementary Figure 6 — (A) Histogram of percentages of B cells staining for CD8 in rhesus macaques. Population defined as CD45+ CD66− CD3− CD20+ CD7− CD8+. (B) CD8 staining in B and T cells for representative animals of each species. (C) Selected mean signaling responses in rhesus macaques with CD8+ B cells. cells (1) still respond to the canonical B cell stimulus CD40L by phosphorylating CREB and degrading IκBα, albeit to a lesser degree than CD8− B cells; (2) unlike T and NK cells, do not degrade IκBα in response to TNFα, do not phosphorylate STAT5 in response to IL-2 and do not phosphorylate STAT4 in response to IFNβ; and (3) have 47% greater pSTAT1 response to IFNβ than CD8−B cells. [file DataSheet_6.pdf]
