# Supplementary material for: A Comprehensive Atlas of Immunological Differences Between Humans, Mice, and Non-Human Primates
Source: Front Immunol. 2022 Mar 11;13:867015. doi: 10.3389/fimmu.2022.867015 (PMC8962947; doi:10.3389/fimmu.2022.867015)
Supplement: Supplementary Figure 4 — Continuation of Figure 5 : Signaling responses in all cell types by stimulus, activation marker, and species. [file DataSheet_4.pdf]

Supplementary Figure 4.

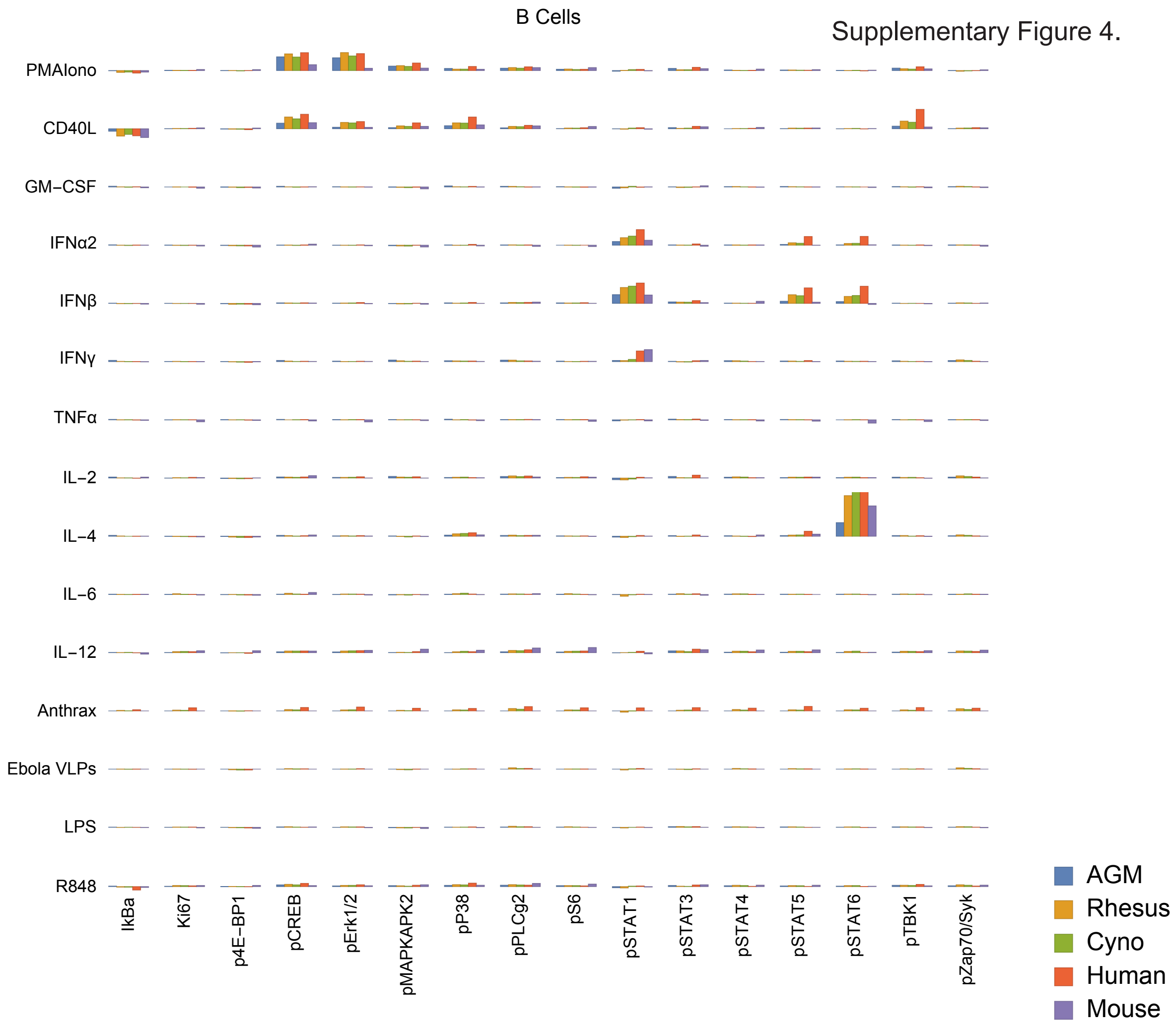

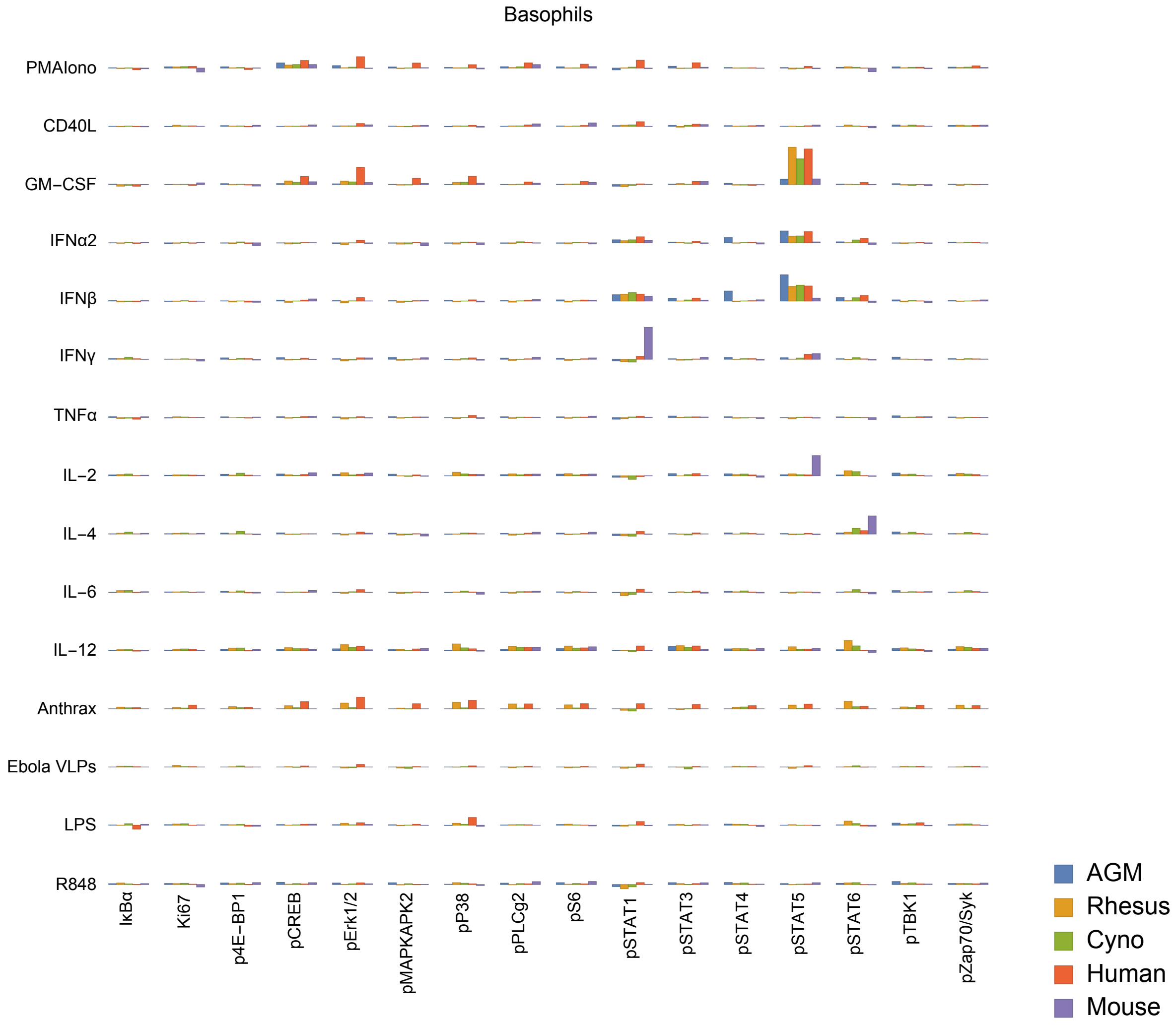

Basophils
PMAIono
CD40L
GM−CSF
IFNα2
IFNβ
IFNγ
TNFα
IL−2
IL−4
IL−6
IL−12
Anthrax
Ebola VLPs
LPS
R848
IκBα
Ki67
p4E−BP1
pCREB
pErk1/2
pMAPKAPK2
pP38
pPLCg2
pS6
pSTAT1
pSTAT3
pSTAT4
pSTAT5
pSTAT6
pTBK1
pZap70/Syk
AGM
Rhesus
Cyno
Human
Mouse

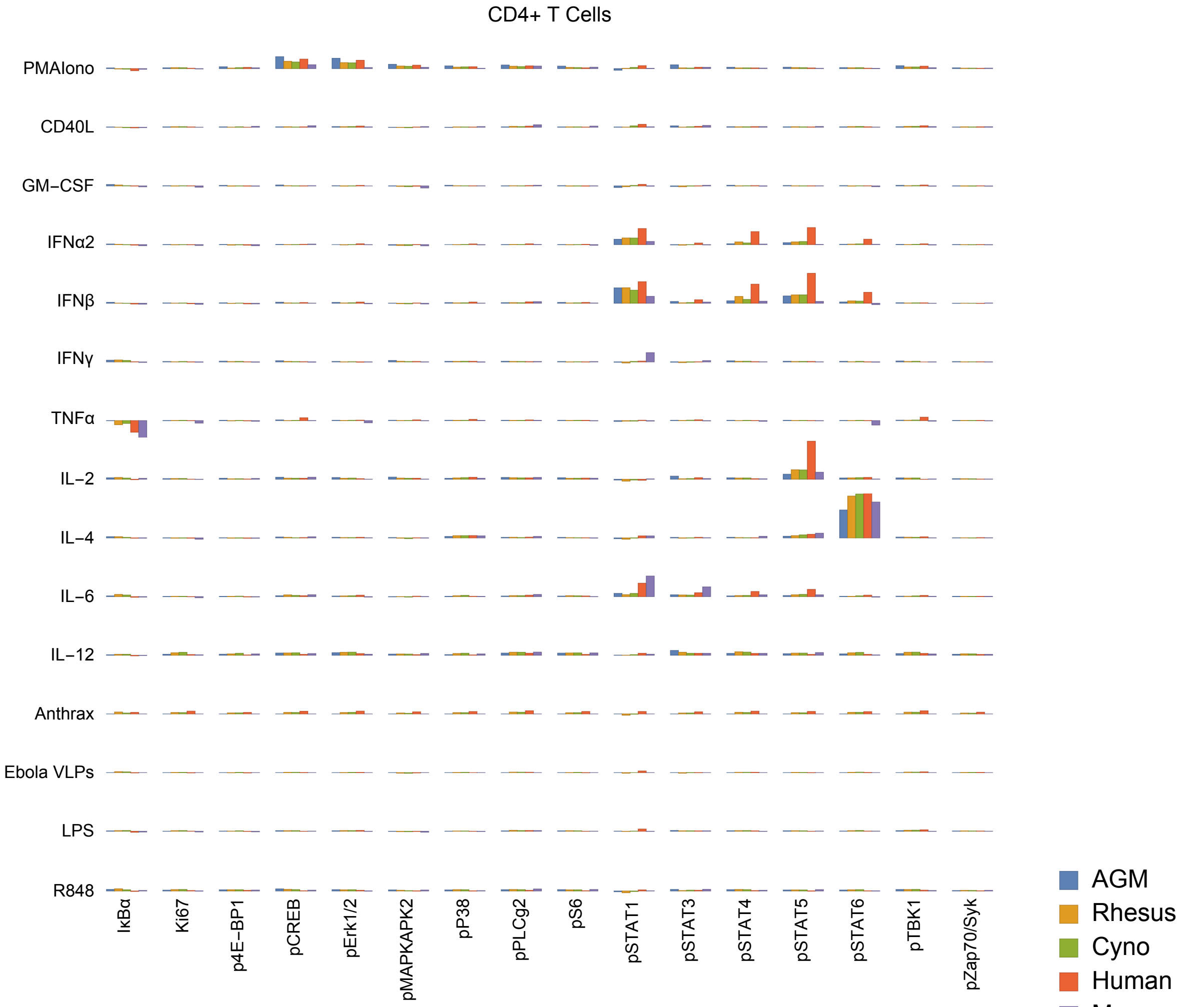
CD4+ T Cells
PMAIono
CD40L
GM−CSF
IFNα2
IFNβ
IFNγ
TNFα
IL−2
IL−4
IL−6
IL−12
Anthrax
Ebola VLPs
LPS
R848
IκBα
Ki67
p4E−BP1
pCREB
pErk1/2
pMAPKAPK2
pP38
pPLCg2
pS6
pSTAT1
pSTAT3
pSTAT4
pSTAT5
pSTAT6
pTBK1
pZap70/Syk
AGM
Rhesus
Cyno
Human
Mouse

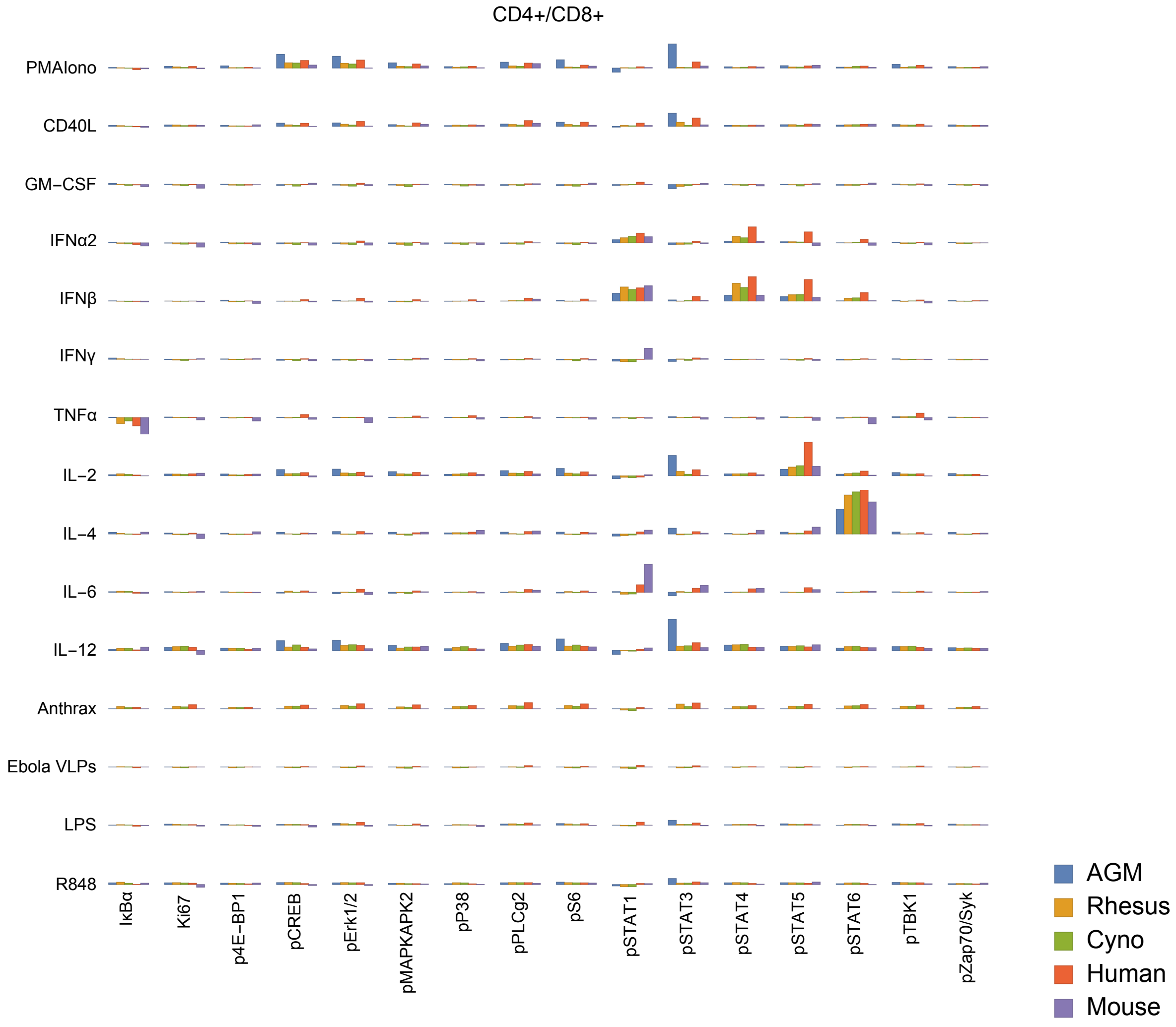
CD4+/CD8+
PMAIono
CD40L
GM−CSF
IFNα2
IFNβ
IFNγ
TNFα
IL−2
IL−4
IL−6
IL−12
Anthrax
Ebola VLPs
LPS
R848
IκBα
Ki67
p4E−BP1
pCREB
pErk1/2
pMAPKAPK2
pP38
pPLCg2
pS6
pSTAT1
pSTAT3
pSTAT4
pSTAT5
pSTAT6
pTBK1
pZap70/Syk
AGM
Rhesus
Cyno
Human
Mouse

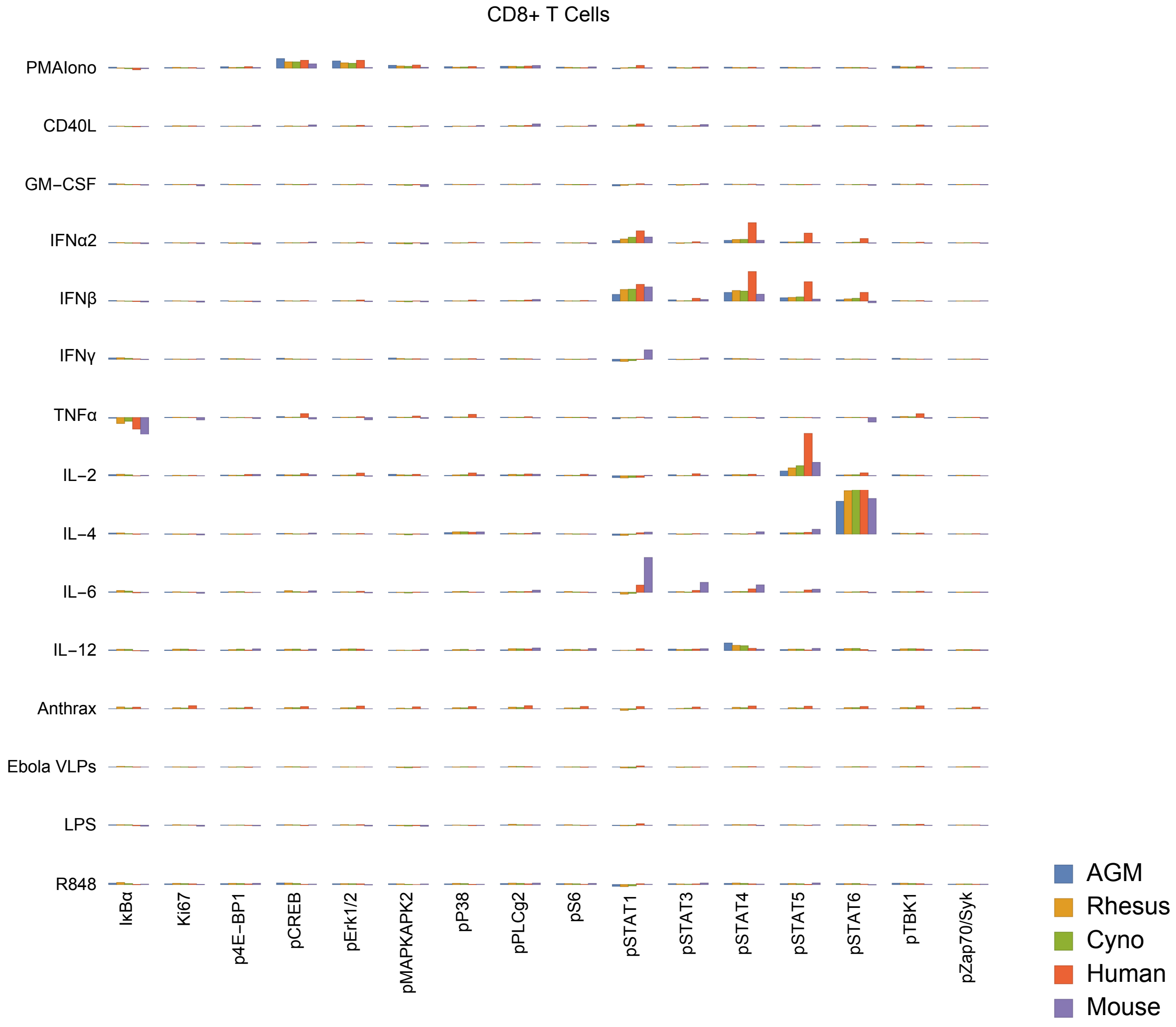
CD8+ T Cells
PMAIono
CD40L
GM−CSF
IFNα2
IFNβ
IFNγ
TNFα
IL−2
IL−4
IL−6
IL−12
Anthrax
Ebola VLPs
LPS
R848
IκBα
Ki67
p4E−BP1
pCREB
pErk1/2
pMAPKAPK2
pP38
pPLCg2
pS6
pSTAT1
pSTAT3
pSTAT4
pSTAT5
pSTAT6
pTBK1
pZap70/Syk
AGM
Rhesus
Cyno
Human
Mouse

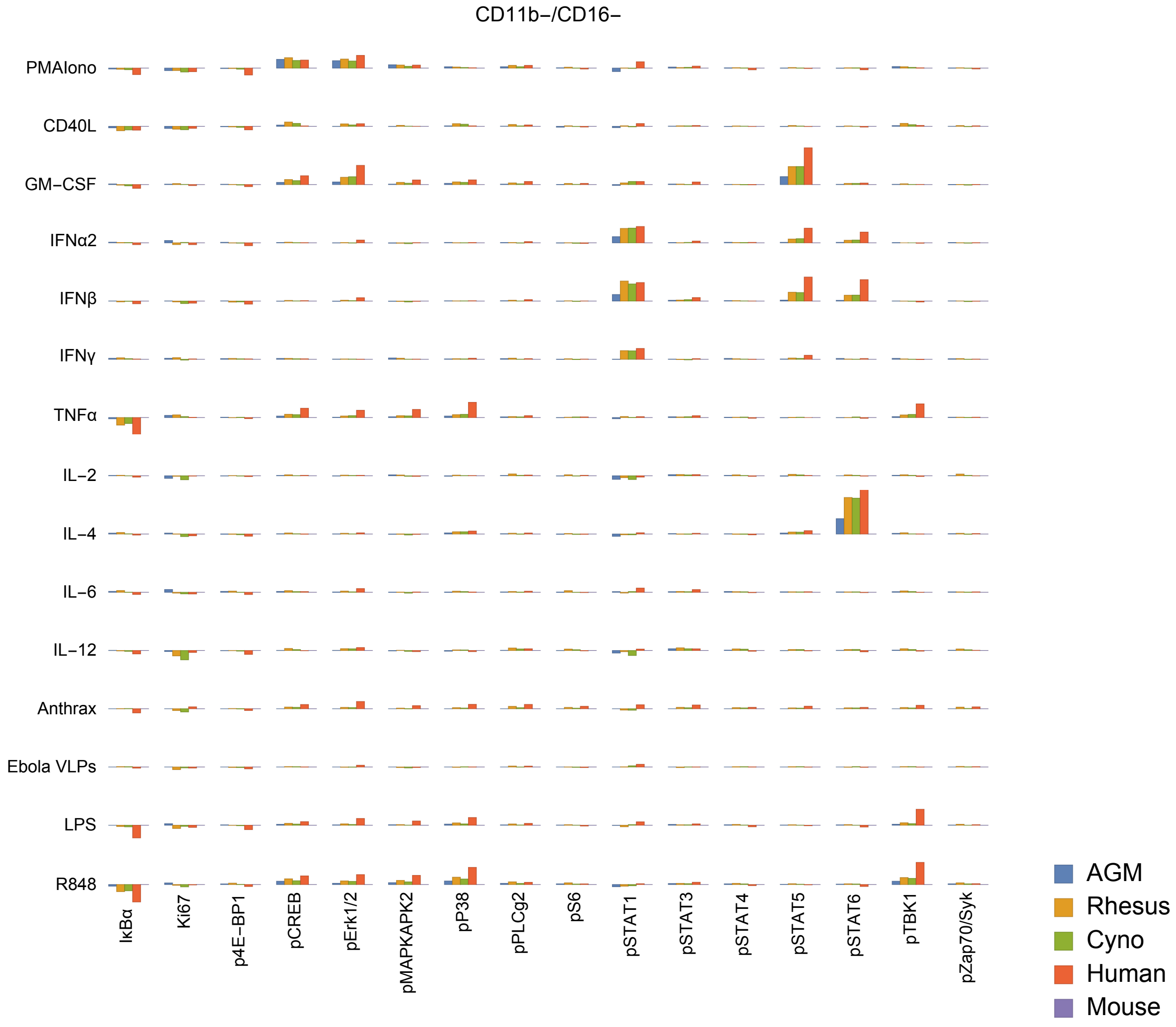
CD11b−/CD16−
PMAIono
CD40L
GM−CSF
IFNα2
IFNβ
IFNγ
TNFα
IL−2
IL−4
IL−6
IL−12
Anthrax
Ebola VLPs
LPS
R848
IκBα
Ki67
p4E−BP1
pCREB
pErk1/2
pMAPKAPK2
pP38
pPLCg2
pS6
pSTAT1
pSTAT3
pSTAT4
pSTAT5
pSTAT6
pTBK1
pZap70/Syk
AGM
Rhesus
Cyno
Human
Mouse

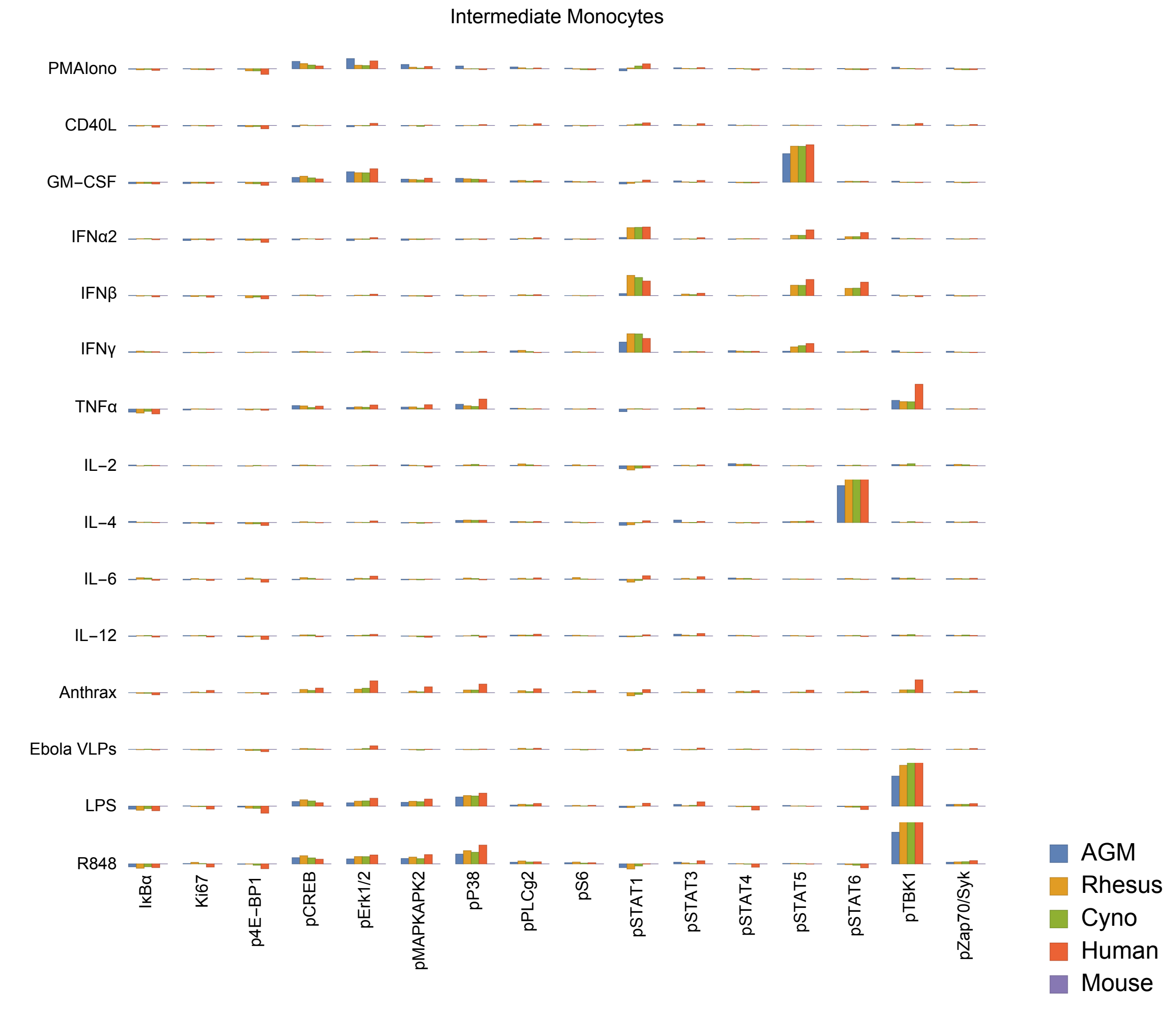

Intermediate Monocytes
PMAIono
CD40L
GM−CSF
IFNα2
IFNβ
IFNγ
TNFα
IL−2
IL−4
IL−6
IL−12
Anthrax
Ebola VLPs
LPS
R848
IkBα
Ki67
p4E−BP1
pCREB
pErk1/2
pMAPKAPK2
pP38
pPLCg2
pS6
pSTAT1
pSTAT3
pSTAT4
pSTAT5
pSTAT6
pTBK1
pZap70/Syk
AGM
Rhesus
Cyno
Human
Mouse

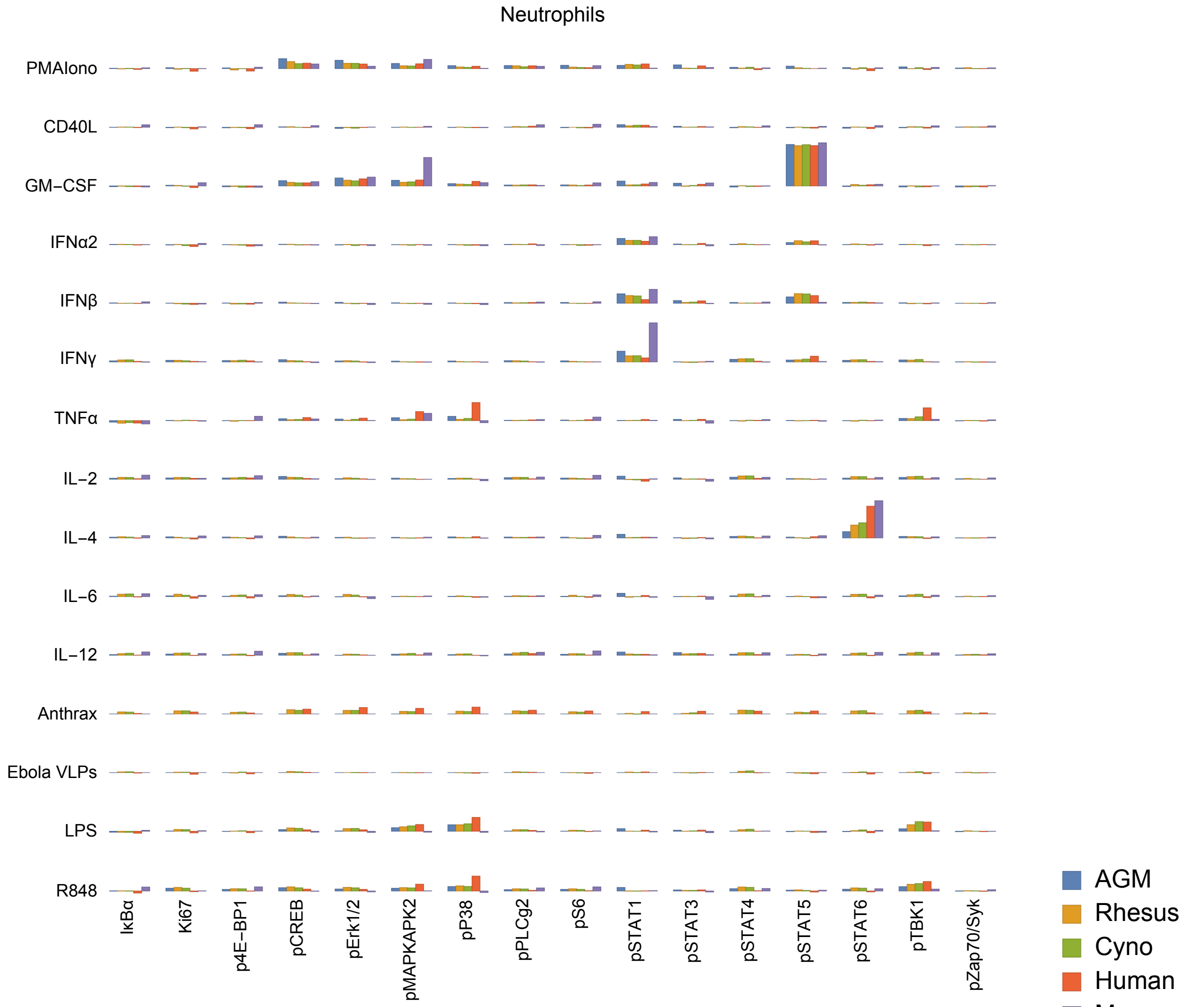
Neutrophils
PMAIono
CD40L
GM−CSF
IFNα2
IFNβ
IFNγ
TNFα
IL−2
IL−4
IL−6
IL−12
Anthrax
Ebola VLPs
LPS
R848
IκBα
Ki67
p4E−BP1
pCREB
pErk1/2
pMAPKAPK2
pP38
pPLCg2
pS6
pSTAT1
pSTAT3
pSTAT4
pSTAT5
pSTAT6
pTBK1
pZap70/Syk
AGM
Rhesus
Cyno
Human
Mouse

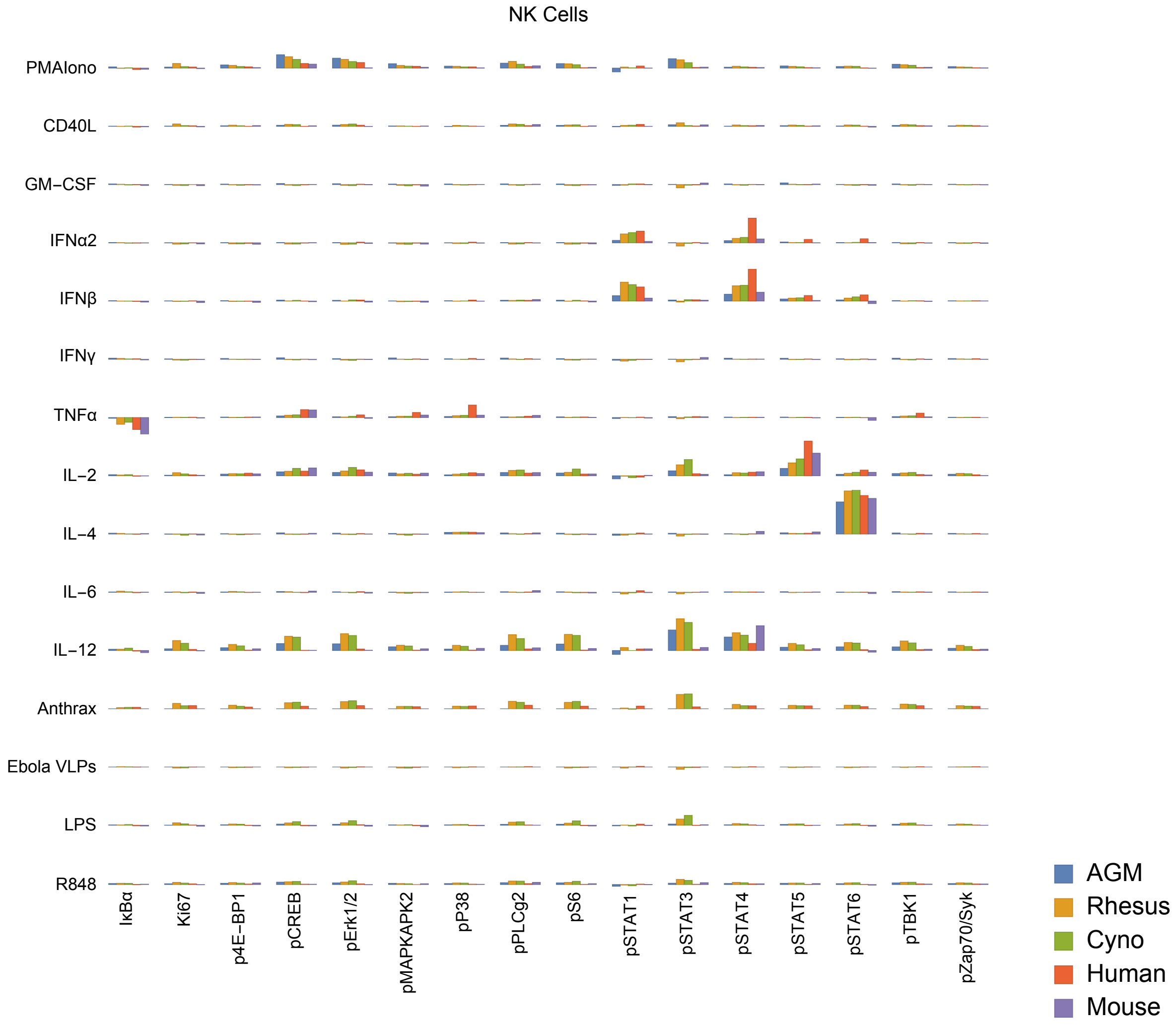

NK Cells
PMAIono
CD40L
GM−CSF
IFNα2
IFNβ
IFNγ
TNFα
IL−2
IL−4
IL−6
IL−12
Anthrax
Ebola VLPs
LPS
R848
IκBα
Ki67
p4E−BP1
pCREB
pErk1/2
pMAPKAPK2
pP38
pPLCg2
pS6
pSTAT1
pSTAT3
pSTAT4
pSTAT5
pSTAT6
pTBK1
pZap70/Syk
AGM
Rhesus
Cyno
Human
Mouse

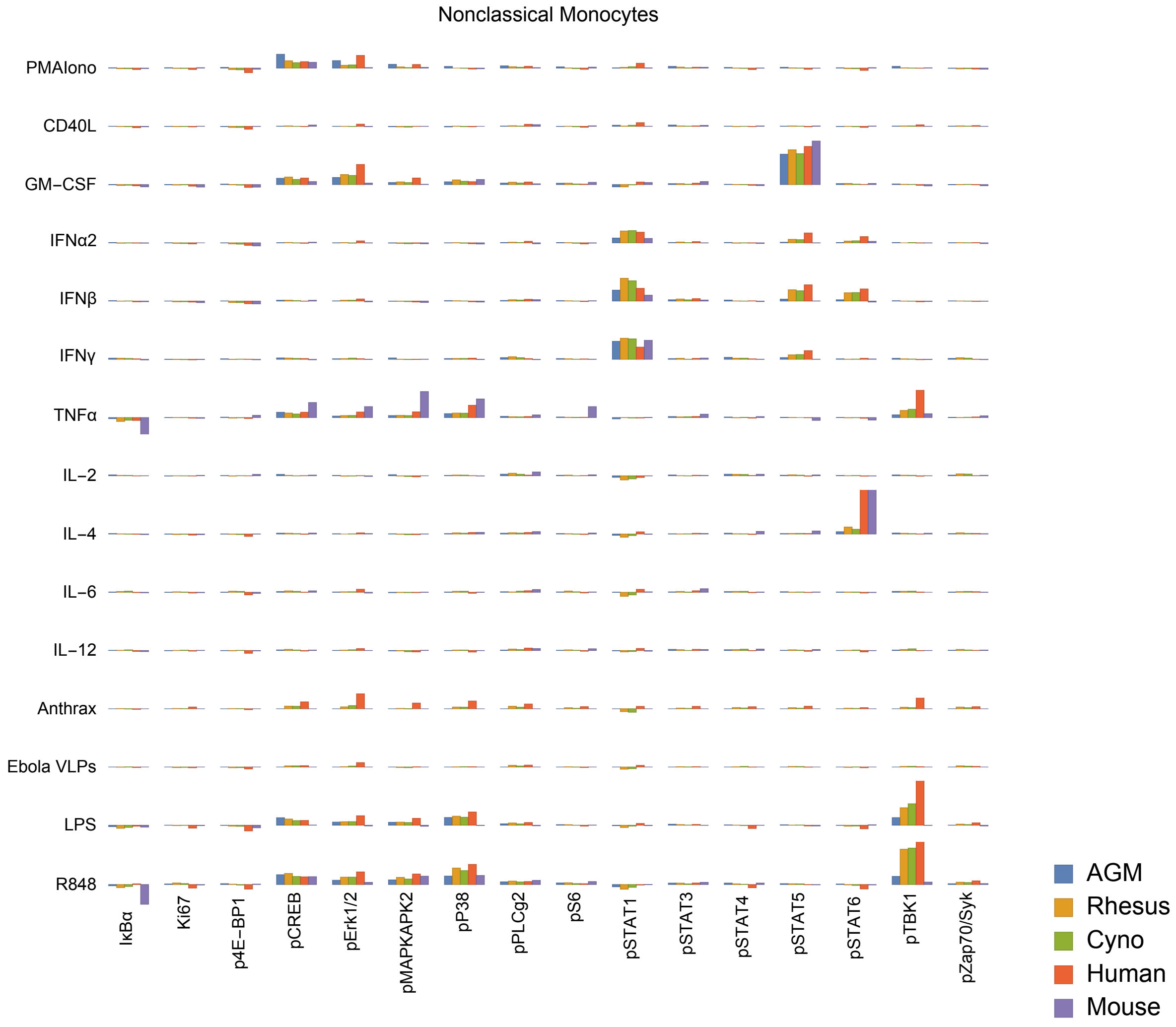
Nonclassical Monocytes
PMAIono
CD40L
GM−CSF
IFNα2
IFNβ
IFNγ
TNFα
IL−2
IL−4
IL−6
IL−12
Anthrax
Ebola VLPs
LPS
R848
IκBα
Ki67
p4E−BP1
pCREB
pErk1/2
pMAPKAPK2
pP38
pPLCg2
pS6
pSTAT1
pSTAT3
pSTAT4
pSTAT5
pSTAT6
pTBK1
pZap70/Syk
AGM
Rhesus
Cyno
Human
Mouse

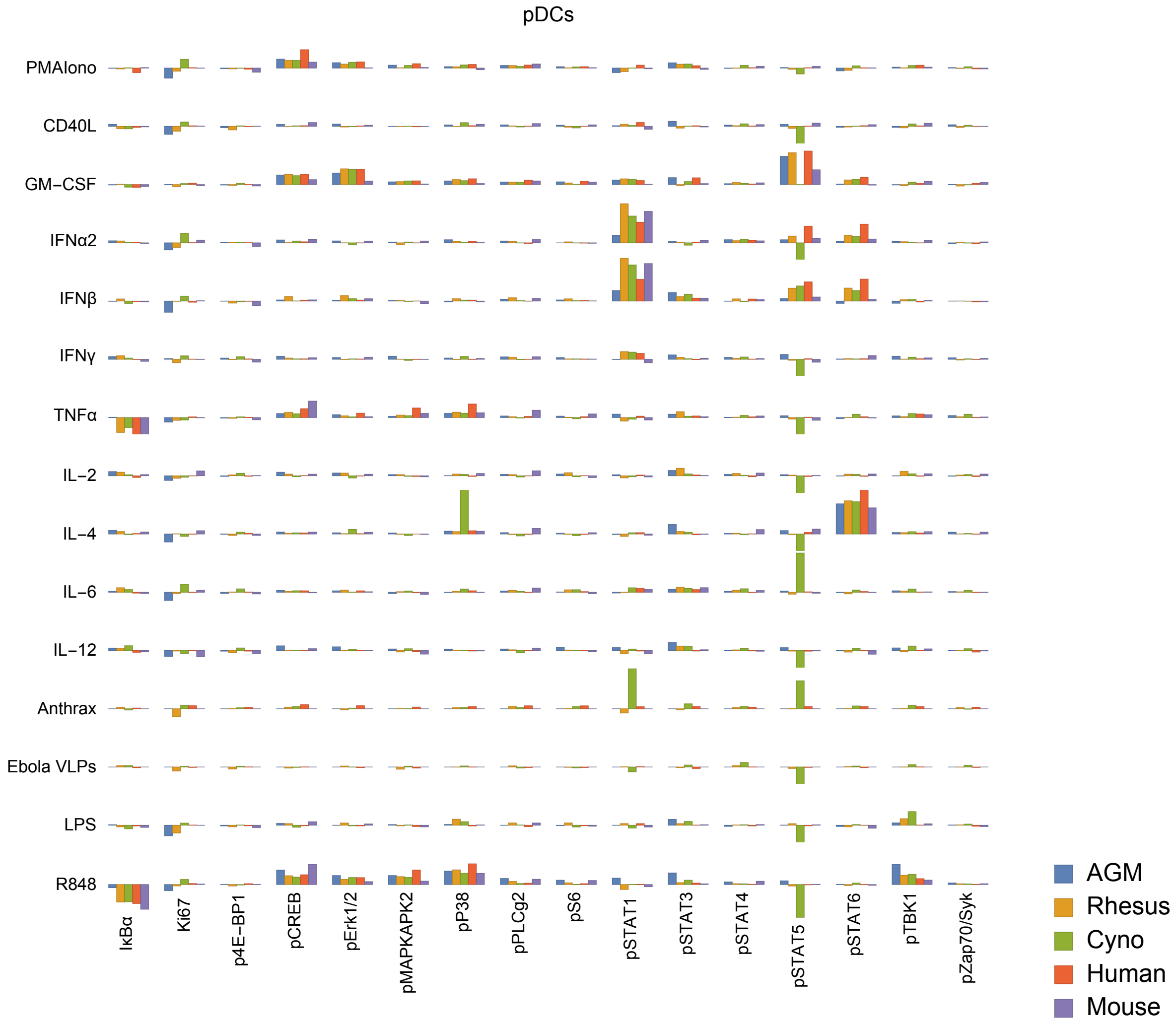

pDCs
PMAIono
CD40L
GM−CSF
IFNα2
IFNβ
IFNγ
TNFα
IL−2
IL−4
IL−6
IL−12
Anthrax
Ebola VLPs
LPS
R848
IκBα
Ki67
p4E−BP1
pCREB
pErk1/2
pMAPKAPK2
pP38
pPLCg2
pS6
pSTAT1
pSTAT3
pSTAT4
pSTAT5
pSTAT6
pTBK1
pZap70/Syk
AGM
Rhesus
Cyno
Human
Mouse
