# Supplementary material for: A Comprehensive Atlas of Immunological Differences Between Humans, Mice, and Non-Human Primates
Source: Front Immunol. 2022 Mar 11;13:867015. doi: 10.3389/fimmu.2022.867015 (PMC8962947; doi:10.3389/fimmu.2022.867015)
Supplement: Supplementary Table 1 — Summary of cell type-specific cross-reactivity. Clones were considered reactive if at least 10 percent of cells had a signal greater than the signal of the 95th percentile of corresponding isotype control or if they were manually classified as positive. Presence of letter in column indicates reactive staining detected in that cell population, absence of letter indicates clone is unreactive in that cell population. B, B cells; T, T cells; N, CD7+ NK cells; M, CD7- monocytes and dendritic cells; G, granulocytes,?: inconclusive, insufficient number of cells acquired for a given population to make a clear assessment. Two of each non-human primate species and one human were assayed. [file Table_1.docx]

Cell type abbreviations:

B: B cells

T: T cells

N: CD7+ NK cells

M: CD7- Myeloid cells (monocytes and dendritic cells)

G: Granulocytes

| **Antibody** | **Clone** | **Human** | **Cyno** | **Rhesus** | **Baboon** | **AGM** |
| --- | --- | --- | --- | --- | --- | --- |
| CD1a | HI149 | T G |  |  |  |  |
| CD1b | SN13 (K5-1B8) | T G | M |  |  | T G |
| CD1c | L161 | B M | B M | B M | B M | B |
| CD1d | 51.1 | B M |  |  |  |  |
| CD2 | RPA-2.10 | TN | BTN | BTN | BTNM | BTN |
| CD3 | HIT3a | T |  |  |  |  |
| CD4 | RPA-T4 | T M | T |  |  |  |
| CD5 | UCHT2 | BT G |  |  |  |  |
| CD6 | BL-CD6 | BTN G |  |  |  |  |
| CD7 | CD7-6B7 | TN |  |  |  |  |
| CD8a | HIT8a | TN |  |  |  |  |
| CD9 | HI9a | ?T?MG | BTNMG | BTNMG | BTNMG | BTNMG |
| CD10 | HI10a | BTN G | MG | G | MG | G |
| CD11a | HI111 | BTNMG | M |  |  |  |
| CD11b | CBRM1/5 | BT G |  |  |  |  |
| CD11b | ICRF44 | BTNMG | B NMG | BTNMG | B NMG | BTNMG |
| CD11c | 3.9 | B MG | B M | B | B M |  |
| CD13 | WM15 | T MG |  |  |  | T MG |
| CD14 | M5E2 | BT MG | MG | MG |  | G |
| CD15 | W6D3 | MG |  |  |  |  |
| CD16 | 3G8 | BTNMG | TNM | TNM | TNM | N |
| CD18 | TS1/18 | BTNMG |  |  |  |  |
| CD19 | HIB19 | B |  | B |  |  |
| CD20 | 2H7 | B | B | B | B N | B |
| CD21 | Bu32 | BT | B |  | B | B |
| CD22 | HIB22 | B |  |  |  |  |
| CD23 | EBVCS-5 | ????? |  |  |  |  |
| CD24 | ML5 | BTNMG |  |  |  |  |
| CD25 | BC96 | BTN |  |  | T |  |
| CD26 | BA5b | TN |  |  |  |  |
| CD27 | O323 | BTN | BTN | BTN | BTNM | BTN |
| CD28 | CD28.2 | TN | T | T | T | T |
| CD29 | TS2/16 | BTNMG | BTNMG | BTNMG | BTNMG | BTNMG |
| CD30 | BY88 |  |  |  |  |  |
| CD31 | WM59 | BTNMG | BTNMG | BTNMG | BTNMG | BTNMG |
| CD32 | FUN-2 | BTNMG | BT MG | BTNMG | BTNMG | B MG |
| CD33 | WM53 | NMG |  |  |  |  |
| CD34 | 581 |  |  |  |  |  |
| CD35 | E11 | ????? | BT MG | BT MG | BT MG | B G |
| CD36 | 5-271 | BTNMG |  |  |  |  |
| CD38 | HIT2 | BTNMG |  |  |  |  |
| CD39 | A1 | BTNMG | BTNMG | MG | BTNMG | B MG |
| CD40 | HB14 | B M | B M | B | B | B M |
| CD41 | HIP8 | BTNMG | BTNMG | BTNMG | BTNMG | BTNMG |
| CD42b | HIP1 | BTNMG |  |  |  |  |
| CD43 | CD43-10G7 | BT?MG | T G |  | T | T MG |
| CD44 | BJ18 | BTNMG | BTNMG | BTNMG | BTNMG | BTN G |
| CD45 | HI30 | BT?MG |  |  |  |  |
| CD45RA | HI100 | BT?M |  |  |  |  |
| CD45RB | MEM-55 | BTNM | BTNMG | BTNM | BTNM | BTN |
| CD45RO | UCHL1 | ????? |  |  |  |  |
| CD46 | TRA-2-10 | BTNMG |  |  |  |  |
| CD47 | CC2C6 | BTNMG | BTNMG | BTNMG | BTNMG | BTNMG |
| CD48 | BJ40 | BTNMG | B M | B MG | B MG | BTNMG |
| CD49a | TS2/7 | TNM | TN | T | T G | TN |
| CD49c | ASC-1 | BTN |  |  |  |  |
| CD49d | 9F10 | BTNMG | BTNMG | BTNMG | BTNMG | BTNMG |
| CD49e | NKI-SAM-1 | TNMG | TNMG | TNMG | BTNMG | BTNMG |
| CD49f | GoH3 | BTNMG | BTNMG | BTNMG | BTNMG | BTNMG |
| CD50 | CBR-IC3/1 | BTNMG | TNM |  | TNMG |  |
| CD51 | NKI-M9 |  |  |  |  |  |
| CD51,CD61 | 23C6 | B M | T |  |  | M |
| CD52 | HI186 | BTNMG | M | M |  |  |
| CD53 | HI29 | BTNMG |  |  |  |  |
| CD54 | HA58 | BTNMG |  |  |  |  |
| CD55 | JS11 | BTNMG | G |  | G | G |
| CD56 | HCD56 | N | NM | NM | NM | N |
| CD57 | HCD57 | N |  |  |  |  |
| CD58 | TS2/9 | BTNMG | BTNMG | BTNMG | BTNMG | BTNMG |
| CD59 | p282 (H19) | BTNMG | BTNMG | BTNMG | BTNMG |  |
| CD61 | VI-PL2 | BTNMG | BTNMG | BTNMG | BTNMG | BTNMG |
| CD62E | HAE-1f |  |  |  |  |  |
| CD62L | DREG-56 | BTNMG |  |  |  |  |
| CD62P | AK4 |  |  |  |  |  |
| CD63 | H5C6 | BTNMG | BTNMG | BTNMG | BTNMG | BTNMG |
| CD64 | 10.1 | MG | MG | NMG | MG | MG |
| CD66a/b/c/e | TET2* | G | G | G | G | G |
| CD66a/c/e | ASL-32 | G |  |  |  |  |
| CD66b | G10F5 | G |  |  |  |  |
| CD69 | FN50 | TNM | TN | TN | G | B N |
| CD70 | 113-16 | T |  |  |  |  |
| CD71 | CY1G4 | BTNM |  |  |  |  |
| CD73 | AD2 | BT G |  |  | TN | TN G |
| CD74 | LN2 | BT MG | B M | B MG | B M | BT MG |
| CD79b | CB3-1 | BTNMG | T G | G | T G | T M |
| CD80 | 2D10 | BTN |  |  |  |  |
| CD81 | 5A6 | BTNMG | T M | BT M | BT MG | BTNMG |
| CD82 | ASL-24 | BTNMG | T G |  | T G | T G |
| CD83 | HB15e | T G |  | T | BT M | BT |
| CD84 | CD84.1.21 | BTNMG | BTNMG | BTNMG | BTNMG | BTNMG |
| CD85 | 17G10.2 |  |  |  |  |  |
| CD85 | GHI/75 | BTNMG |  |  |  |  |
| CD85 | MKT5.1 | T MG | MG | MG | G | G |
| CD85d | 42D1 | T MG |  |  |  |  |
| CD85h | 24 | MG |  | G |  |  |
| CD85k | ZM4.1 | M |  |  |  |  |
| CD86 | IT2.2 | BT MG | B M | B MG | BT MG | BT MG |
| CD87 | VIM5 | T MG | MG | BTNMG | MG | NMG |
| CD88 | S5/1 | BTNMG |  |  | G | T |
| CD89 | A59 | NMG | NMG | NMG | MG | T G |
| CD90 | 5E10 |  | BTNMG | BTNMG | BTNMG | BTNMG |
| CD93 | VIMD2 | M | MG | MG | TNMG | TNMG |
| CD94 | DX22 | TN | T G |  | T | T |
| CD95 | DX2 | BTNMG | BTNMG | BTNMG | BTNMG | BTNMG |
| CD96 | NK92.39 | TN G |  |  |  |  |
| CD97 | VIM3b | BTNMG |  | B |  |  |
| CD99 | HCD99 | BT?MG | TN |  | T | T MG |
| CD100 | A8 | BTNMG | BTNM | BTNM | BTNMG | BTNM |
| CD101 | BB27 | BT MG | T MG | BTNMG | T MG | MG |
| CD102 | CBR-IC2/2 | BTNMG | BTNMG | BTNM | BTNM | BTNMG |
| CD103 | Ber-ACT8 |  |  |  |  |  |
| CD104 | 58XB4 |  |  |  |  |  |
| CD105 | 43A3 | NM |  |  |  |  |
| CD106 | STA |  |  |  |  |  |
| CD107a | H4A3 | BTNMG | T G | BTN | BT MG | BTNMG |
| CD108 | MEM-150 | B NM | B | B | B | B |
| CD109 | W7C5 | TNM | NM |  |  | TN |
| CD111 | R1.302 | N |  |  |  |  |
| CD112 | TX31 | T MG |  |  |  |  |
| CD114 | LMM741 | NMG | G |  |  |  |
| CD115 | 9-4D2-1E4 | G | G | TN G | M | T G |
| CD116 | 4H1 | T MG |  |  |  | T |
| CD117 | 104D2 | N |  |  |  |  |
| CD119 | GIR-208 | BTNMG | BTNMG | BTNMG | BTNMG | BTNMG |
| CD122 | TU27 | TN | N | N | N | N |
| CD123 | 6H6 | B NM |  |  |  |  |
| CD124 | G077F6 | B |  |  |  |  |
| CD126 | UV4 | TNMG | T M | T | T | T |
| CD127 | A019D5 | TN | T | T | TN | T |
| CD129 | AH9R7 | TN |  | TN |  | T G |
| CD131 | 1C1 | MG | MG | MG | MG | G |
| CD132 | TUGh4 | BTNMG | MG | G |  | T MG |
| CD134 | Ber-ACT35 (ACT35) | T |  |  |  |  |
| CD135 | BV10A4H2 |  |  |  |  |  |
| CD137 | 4B4-1 |  |  |  |  |  |
| CD138 | DL-101 | TNMG | T G |  | T MG | T G |
| CD140a | 16A1 |  |  |  |  |  |
| CD140b | 18A2 | G | G |  | T G | T G |
| CD141 | M80 | MG |  |  | M | MG |
| CD143 | 5-369 | T G |  |  |  |  |
| CD144 | BV9 |  |  |  |  |  |
| CD146 | SHM-57 | BTNMG | MG |  | BTNMG | T MG |
| CD148 | A3 | BTNMG |  |  |  |  |
| CD150 | A12 (7D4) | BT | BT | BT | BT | BT |
| CD152 | L3D10 |  |  |  |  |  |
| CD154 | 24-31 | BTNMG |  |  |  |  |
| CD155 | SKII.4 | NMG |  |  |  |  |
| CD156c | SHM14 | BTNMG | BTNMG | BTNMG | BTNMG | BTNMG |
| CD158a/h | HP-MA4 | N | N |  | N G |  |
| CD158b | DX27 | N |  |  |  |  |
| CD158d | mAb 33 (33) | TN G |  |  |  |  |
| CD158e1 | DX9 | N |  |  |  |  |
| CD158f | UP-R1 | T MG |  |  |  |  |
| CD161 | HP-3G10 | TNMG | TN | TNM | TNMG | N |
| CD162 | KPL-1 | BTNMG | BTNMG | BTNMG | BTNMG | BTNMG |
| CD163 | GHI/61 | M | MG | MG |  | G |
| CD164 | 67D2 | BTNMG | BTNMG | BTNMG | BTNMG | BTNMG |
| CD165 | SN2 (N6-D11) | BTNMG | BTNMG | B | BTNMG | BTNMG |
| CD166 | 3A6 | BTNMG | BTNMG | BT MG | BT MG | BTNMG |
| CD167a | 51D6 |  |  |  |  |  |
| CD169 | 7-239 |  |  |  |  |  |
| CD170 | 1A5 | NMG |  |  |  |  |
| CD172a | SE5A5 | T MG | T MG | TNMG | MG | MG |
| CD172b | B4B6 | MG |  |  |  |  |
| CD172g | LSB2.20 | T G | MG | TNMG | TNMG | MG |
| CD178 | NOK-1 | G |  |  | MG |  |
| CD179a | HSL96 |  |  |  |  | T |
| CD179b | HSL11 |  |  |  |  | BT M |
| CD180 | MHR73-11 | B M | B M | B M | B M | B M |
| CD181 | 8F1/CXCR1 | TNMG |  |  |  |  |
| CD182 | 5E8/CXCR2 | NMG |  |  |  |  |
| CD183 | G025H7 | BTNMG | BTNMG | BTNM | BTNMG | BTNMG |
| CD184 | 12G5 | BTNMG | BTNMG | BTNMG | BTNMG | BTNMG |
| CD193 | 5E8 | T M |  |  |  |  |
| CD195 | T21/8 | BTNMG | T MG | MG | T M | T MG |
| CD196 | G034E3 | BTNM | BTN G | BTN | BTN | BTNMG |
| CD197 | G043H7 | BT G | BT | BT | BTN | BT |
| CD200 | OX-104 | BT G |  |  | BT | B |
| CD200R | OX-108 | BTNMG |  |  | BT MG |  |
| CD201 | RCR-401 |  |  |  |  |  |
| CD202b | 33.1 (Ab33) |  |  |  |  |  |
| CD203c | NP4D6 |  |  |  |  |  |
| CD205 | HD30 | G | BT MG | T MG | T MG | TNMG |
| CD206 | 15-2 |  |  |  |  |  |
| CD207 | 10E2 |  |  |  |  |  |
| CD209 | 9E9A8 |  |  |  |  |  |
| CD210 | 3F9 | BTNMG | BTNM | M | BTN | BTNMG |
| CD213a2 | SHM38 | T G |  | T |  |  |
| CD215 | JM7A4 | T G | T G | TN G | T G | T G |
| CD218a | H44 | TNMG | N | TN | TN | TN G |
| CD220 | B6.220 | M | MG | M | M | MG |
| CD221 | 1H7/CD221 | TNMG | T MG | T | BTN G | N G |
| CD226 | 11A8 | BTNMG | BTNMG | BTNMG | BTNMG | BTNMG |
| CD229 | HLy-9.1.25 | BTN |  |  |  |  |
| CD231 | SN1a (M3-3D9) | T G | G |  | T | T |
| CD235ab | HIR2 |  |  |  |  | B |
| CD243 | UIC2 | BTNMG |  |  | T | T |
| CD244 | C1.7 | TNM |  |  |  |  |
| CD245 | DY12 | TNMG | M |  | TN | M |
| CD252 | 11C3.1 | G |  |  |  | T G |
| CD253 | RIK-2 | TN G |  |  |  | N |
| CD254 | MIH24 | G |  |  |  |  |
| CD255 | CARL-1 |  |  |  | T | T |
| CD257 | T7-241 | BT MG | T G | TN G | T MG | BT MG |
| CD258 | T5-39 |  |  | TN |  |  |
| CD261 | DJR1 | G | T |  |  | T |
| CD262 | DJR2-4 (7-8) | T MG |  |  |  |  |
| CD263 | DJR3 | G |  |  |  |  |
| CD266 | ITEM-1 |  |  |  |  | T |
| CD267 | 1A1 | B | B | B | B | B |
| CD268 | 11C1 | BT | BT M | B | BT MG | BT MG |
| CD271 | ME20.4 | N | TNM | TNM | BTNM | TNM |
| CD272 | MIH26 | BT |  |  |  | G |
| CD273 | 24F.10C12 | T G | TNM | TNM | T M | M |
| CD274 | 29E.2A3 | BTNMG | T G | N G | T MG | BTNMG |
| CD275 | 9F.8A4 | B |  |  |  |  |
| CD276 | MIH42 | TN G | N | N |  | TN |
| CD277 | BT3.1 | BTNMG | B | B | BTNM | BTN |
| CD278 | C398.4A | TN | T | T | T | T |
| CD279 | EH12.2H7 | T | T | T | T | T |
| CD282 | TL2.1 | B MG |  |  |  |  |
| CD284 | HTA125 | T M |  |  |  |  |
| CD286 | TLR6.127 | MG |  |  |  |  |
| CD290 | 3C10C5 | B |  |  | B |  |
| CD294 | BM16 | MG |  |  |  |  |
| CD298 | LNH-94 | BTNMG | BTNMG | BTNMG | BTNMG | BTNMG |
| CD300e | UP-H2 | M |  |  |  |  |
| CD300F | UP-D2 | M |  |  |  |  |
| CD301 | H037G3 | BT MG | T MG |  | BT MG | BT MG |
| CD303 | 201A |  |  |  |  |  |
| CD304 | 12C2 |  |  |  |  |  |
| CD307e | 509f6 | B |  |  |  |  |
| CD314 | 1D11 | TN G | TN | TN | TN | N |
| CD317 | RS38E | BTNMG | BTNMG | BTNM | BTNMG | BTNMG |
| CD318 | CUB1 |  |  |  |  |  |
| CD319 | 162.1 | BTNM | G |  |  |  |
| CD324 | 67A4 |  |  |  |  |  |
| CD325 | 8C11 | G |  |  |  |  |
| CD326 | 9C4 |  |  |  |  |  |
| CD328 | 6-434 | NMG |  |  |  |  |
| CD334 | 4FR6D3 |  |  |  |  |  |
| CD335 | 9E2 | N |  |  |  |  |
| CD336 | P44-8 |  |  |  |  |  |
| CD337 | P30-15 | N | N | N | N | N |
| CD338 | 5D3 |  |  |  |  |  |
| CD340 | 24D2 |  |  |  |  |  |
| CD344 | CH3A4A7 | TNMG |  |  |  | TN G |
| CD351 | TX61 | N G |  |  |  |  |
| CD352 | NT-7 | BTNMG | T MG |  | T G | T |
| CD354 | TREM-26 | TNMG | MG | T G | MG | G |
| CD355 | Cr24.1 |  |  |  |  |  |
| CD357 | 621 | TNMG | T G | TN G | T G | T MG |
| CD360 | 2G1-K12 | BTNM | BTNM | BTNM | BTNM | B NM |
| 4-1BB Ligand | 5F4 | TNMG | T G | TN | T G | TNMG |
| C3aR | hC3aRZ8 | TNMG |  |  |  |  |
| C5L2 | 1D9-M12 | TNMG | BTNMG | BTNMG | BTNMG | BTNMG |
| CCR10 | 6588-5 | BTNMG | B MG | BTNMG | BT MG | BT MG |
| CLEC12A | 50C1 | MG |  |  |  |  |
| CLEC9A | 8F9 |  |  |  |  |  |
| CX3CR1 | 2A9-1 | TNMG | MG | G |  | M |
| CXCR7 | 8F11-M16 |  |  |  |  |  |
| Delta Opioid Receptor | DOR7D2A4 |  |  |  |  |  |
| DLL1 | MHD1-314 |  |  |  |  |  |
| DLL4 | MHD4-46 |  |  |  |  |  |
| DR3 | JD3 |  |  |  |  |  |
| EGFR | AY13 |  |  |  |  |  |
| erbB3 | 1B4C3 |  |  |  |  |  |
| FcRL4 | 413D12 |  |  |  |  |  |
| FcRL6 | 2H3 | N |  |  |  | B |
| FcεRIα | AER-37 (CRA-1) | M | M | M | M |  |
| Galectin-9 | 9M1-3 | T G | BT G |  | BT G | T |
| GARP | 7B11 |  |  |  |  |  |
| HLA-A2 | BB7.2 |  |  |  |  |  |
| HLA-A,B,C | W6/32 | BTNMG | BTNMG | BTNMG | BTNMG | BTNMG |
| HLA-DQ | HLADQ1 | B |  |  |  |  |
| HLA-DR | L243 | BTNM | B MG | BTNMG | BTNMG | BTNMG |
| HLA-E | 3D12 | BTNMG |  |  |  |  |
| HLA-G | 87G |  | MG | MG |  | M |
| HVEM | 122 | BTNMG | BTNMG | BTNM | BTNMG | BTNMG |
| IFN-γ R b chain | 2HUB-159 | T MG | G | T | T | T MG |
| IgD | IA6-2 | B |  |  |  |  |
| IgG1, κ Isotype Ctrl | MOPC-21 |  |  |  |  |  |
| IgG1, κ Isotype Ctrl | RTK2071 |  |  |  |  |  |
| IgG2a, κ Isotype Ctrl | MOPC-173 |  |  |  |  |  |
| IgG2a, κ Isotype Ctrl | RTK2758 |  |  |  |  |  |
| IgG2b, κ Isotype Ctrl | MPC-11 |  |  |  |  |  |
| IgG2b, κ Isotype Ctrl | RTK4530 |  |  |  |  |  |
| IgG3,k Isotype Ctrl | MG3-35 |  |  |  |  |  |
| IgG Isotype Ctrl | HTK888 |  |  |  |  |  |
| Ig light chain κ | MHK-49 | B MG | G |  |  |  |
| Ig light chain λ | MHL-38 | B M | B MG | B MG | B MG |  |
| IgM | MHM-88 | B NMG | B MG | B NMG | BTNMG | BTNMG |
| IgM, κ Isotype Ctrl | MM-30 |  |  |  |  |  |
| IgM, κ Isotype Ctrl | RTK2118 |  |  |  |  |  |
| IL-28RA | MHLICR2a |  |  |  |  |  |
| Integrin α9β1 | Y9A2 | MG | G | N | N | N G |
| integrin β5 | AST-3T |  | G |  |  |  |
| Integrin β7 | FIB504 | BTNMG | BTNM | BTNMG | BTNMG | BTNMG |
| Jagged 2 | MHJ2-523 | N |  |  |  |  |
| LAP | TW4-6H10 | M | MG |  | M | T M |
| Lymphotoxin β Receptor | 31G4D8 | M | M | M |  | BT MG |
| Mac-2 | Gal397 | T G | T G |  | T G | T G |
| MAIR-II | TX45 | ????? | T G | T | T | T G |
| MICA/MICB | 6D4 |  |  |  |  |  |
| MSC | W7C6 | T | T | T G | T M | T |
| MSC,NPC | W4A5 | BT MG | T G | BTNMG | BT MG | BT MG |
| NKp80 | 5D12 | N | TN | TNM | TNM | TN |
| Notch 1 | MHN1-519 | N |  |  |  |  |
| Notch 2 | MHN2-25 | M |  |  |  | M |
| Notch3 | MHN3-21 |  |  |  |  |  |
| Notch 4 | MHN4-2 |  |  |  |  |  |
| NPC | 57D2 | ????? |  |  |  | T |
| Podoplanin | NC-08 |  |  |  |  |  |
| Pre-BCR | HSL2 | MG | M | M |  |  |
| PSMA | LNI-17 |  |  |  |  |  |
| Siglec-10 | 5G6 | B M |  |  |  | MG |
| Siglec-8 | 7C9 | N |  |  |  |  |
| Siglec-9 | K8 | NMG |  |  |  |  |
| SSEA-1 | MC-480 | G |  |  |  |  |
| SSEA-3 | MC-631 |  |  |  |  | B |
| SSEA-4 | MC-813-70 |  | MG | G | G |  |
| SSEA-5 | 8E11 | T |  |  |  | T |
| SUSD2 | W3D5 | B |  | TN |  | BT MG |
| SUSD2 | W5C5 | N | TNM |  | G | BTNMG |
| TCR gamma/delta | B1 | T G | T | TN | TN | T |
| TCR Vα24-Jα18 | 6B11 |  |  |  |  |  |
| TCR Vα7.2 | 3C10 | T |  |  |  |  |
| TCR Vβ13.2 | H132 | ????? |  |  |  |  |
| TCR Vβ23 | αHUT7 |  |  |  |  |  |
| TCR Vβ8 | JR2 (JR.2) |  |  |  |  |  |
| TCR Vβ9 | MKB1 |  |  |  |  |  |
| TCR Vδ2 | B6 |  |  |  |  |  |
| TCR α/β | IP26 | T |  |  |  |  |
| Tim-1 | 1D12 | N G |  |  |  |  |
| Tim-3 | F38-2E2 | N |  |  |  |  |
| Tim-4 | 9F4 |  |  |  |  |  |
| TLT-2 | MIH61 | ????? |  |  |  |  |
| TNAP | W8B2 | G | G |  | B MG | B G |
| TRA-1-60-R | TRA-1-60-R |  |  |  |  |  |
| TRA-1-81 | TRA-1-81 | N |  |  |  |  |
| TSLPR | 1B4 |  |  |  | M |  |
| Vγ9 | B3 |  |  |  |  |  |
| β2-microglobulin | 2M2 | BT?MG | B N | B | B | B |
