# Supplementary material for: A Comprehensive Atlas of Immunological Differences Between Humans, Mice, and Non-Human Primates
Source: Front Immunol. 2022 Mar 11;13:867015. doi: 10.3389/fimmu.2022.867015 (PMC8962947; doi:10.3389/fimmu.2022.867015)
Supplement: Supplementary Table 3 — Studies reporting frequencies of DP T cells in macaques. *SIV infected. †denotes frequencies calculated from Lee et al.’s Table 1; we report the mean of the means for each of the DP-high/middle/low-frequency groups to correct for bias arising from unequal group sizes. [file Table_3.docx]

| **Study** | **PMID** | **Species** | **Origin** | **Age** | **% PBL** |
| --- | --- | --- | --- | --- | --- |
| This |  | Rhesus | China | 5-9 years | 5.3 |
| This |  | Cyno | China | 5-9 years | 1.4 |
| Akari 1997 | 9138020 | Cyno | Indonesia, Philippines, Malaysia | 0-4 years  5-9 years  10-14 years  >15 years | 1.2  2.7  9.4  8.7 |
| Lee 2003 | 12757616 | Cyno | Not specified | 6.5 years  7.3 years  7.7 years  8.8 years  9.4 years  10.0 years  11.0 years | 3.1†  5.1  4.5  4.7  7.1  5.0  6.0 |
| Reimann 1994 | 8001455 | Rhesus | Not specified | Not specified | <10 |
| Dean 1996 | 8764081 | Rhesus | Not specified | Not specified | 5.8* |
| Wang 2008 | 18820133 | Rhesus | India | 0 to 3 days  12 to 21 days | ~2  ~4 |
