# Supplementary material for: A Comprehensive Atlas of Immunological Differences Between Humans, Mice, and Non-Human Primates
Source: Front Immunol. 2022 Mar 11;13:867015. doi: 10.3389/fimmu.2022.867015 (PMC8962947; doi:10.3389/fimmu.2022.867015)
Supplement: Supplementary Table 4 — Demographics of human donors. Age (years), height (cm), weight (kg), and BMI information of 39 females and 44 male human donors. For one individual, do not have additional information on. [file Table_4.docx]

|  |  | **Mean** | **SD** | **Min** | **Max** |
| --- | --- | --- | --- | --- | --- |
| **Female (n = 39)** | **Age (years)** | 38.16 | 11.13 | 22.00 | 63.00 |
|  | **Height (cm)** | 164.84 | 6.16 | 152.40 | 177.80 |
|  | **Weight (kg)** | 71.81 | 18.76 | 51.71 | 127.01 |
|  | **BMI** | 26.32 | 6.02 | 19.57 | 42.59 |
| **Male (n = 44)** | **Age (years)** | 33.86 | 11.55 | 19.00 | 61.00 |
|  | **Height (cm)** | 176.59 | 9.27 | 152.40 | 193.04 |
|  | **Weight (kg)** | 81.54 | 15.95 | 54.88 | 151.50 |
|  | **BMI** | 26.15 | 4.44 | 19.63 | 40.67 |
| **Total (n = 86)** | **Age (years)** | 35.85 | 11.49 | 19.00 | 63.00 |
|  | **Height (cm)** | 171.07 | 9.87 | 152.40 | 193.04 |
|  | **Weight (kg)** | 76.97 | 17.90 | 51.71 | 151.50 |
|  | **BMI** | 26.23 | 5.21 | 19.57 | 42.59 |
