# Supplementary material for: A Comprehensive Atlas of Immunological Differences Between Humans, Mice, and Non-Human Primates
Source: Front Immunol. 2022 Mar 11;13:867015. doi: 10.3389/fimmu.2022.867015 (PMC8962947; doi:10.3389/fimmu.2022.867015)
Supplement: Supplementary Table 5 — Stimuli. Stimuli marked with an asterisk (*) were dispensed at time of usage due to production and/or storage requirements. [file Table_5.docx]

| **Stimulus** | **Host Species** | **Used In** | **Produced In** | **Vendor** | **Catalog** | **Lot** | **Stimulation Concentration** |
| --- | --- | --- | --- | --- | --- | --- | --- |
| GM-CSF | Rhesus | Rhesus  Cyno | E. coli | Cell Sciences | CRG134B | 3600102 | 100 ng/ml |
| GM-CSF | Human | Human | E. coli | Cell Sciences | CRG100B | 3112711 | 100 ng/ml |
| GM-CSF | Mouse | Mouse | E. coli | Cell Sciences | CRG101A | 3201617 | 100 ng/ml |
| IFNα2 | Rhesus/Cyno (identical) | Rhesus  Cyno | E. coli | PBL Assay Science | 14110-1 | 5967, 5616 pooled | 150 ng/ml |
| IFNα2 | Human | Human | E. coli | PBL Assay Science | 11100-1 | 5962 | 150 ng/ml |
| IFNα2 | Mouse | Mouse | E. coli | eBioscience | 14-8312-80 | E05729-1634 | 150 ng/ml |
| LPS | N/A | Human  Rhesus  Cyno  Mouse | E. coli O111:B4 | InVivoGen | tlrl-eblps | LEB-36-01 | 1 μg/ml |
| IL-6 | Rhesus | Rhesus  Cyno | E. coli | Cell Sciences |  | 3600502 | 500 ng/ml |
| IL-6 | Human | Human | E. coli | Cell Sciences | CRI106B | 3103810 | 500 ng/ml |
| IL-6 | Mouse | Mouse | E. coli | Cell Sciences | CRI130B | 3201609 | 500 ng/ml |
| Resiquimod (R848) | N/A | Human  Rhesus  Cyno  Mouse | N/A | InVivoGen | tlrl-r848-5 | 848-35-14 | 10 μg/ml |
| IFNγ | Rhesus | Rhesus  Cyno | E. coli | R&D Systems | 961-RM-025 | ETZ0213071 | 330 ng/ml |
| IFNγ | Human | Human | E. coli | R&D Systems | 285-IF-100 | RAX1814011 | 330 ng/ml |
| IFNγ | Mouse | Mouse | E. coli | PeproTech | 315-05A | 061398 | 330 ng/ml |
| TNFα | Rhesus | Rhesus  Cyno | E. coli | R&D Systems | 1070-RM-025 | DCSD0113081 | 100 ng/ml |
| TNFα | Human | Human | E. coli | R&D Systems | 210-TA-020 | DDHB0113062 | 100 ng/ml |
| TNFα | Mouse | Mouse | E. coli | R&D Systems | 410-MT-010 | CS1313081 | 100 ng/ml |
| IFNβ* | Human | Human  Rhesus  Cyno | CHO | PBL Assay Science | 11415-2 | 5886 | 5 ng/ml |
| IFNβ | Mouse | Mouse | Human cell line | PBL Assay Science | 12405-1 | 5900 | 5 ng/ml |
| CD40L soluble dimer (“MegaCD40L”) | Human | Human  Rhesus  Cyno | CHO | Enzo Life Sciences | ALX-522-110-C010 | 05281412, 03041401 pooled | 125 ng/ml |
| CD40L soluble dimer (“MegaCD40L”) | Mouse | Mouse | CHO | Enzo Life Sciences | ALX-522-120-C010 | 01151321 | 125 ng/ml |
| PMA and ionomycin* | N/A | Human  Rhesus  Cyno  Mouse | N/A | eBioscience | 00-4970 | E13495-116 | 0.081 μM PMA and 1.34 μM ionomycin |
| IL-12 | Rhesus | Rhesus  Cyno | CHO | R&D Systems | 3216-RL-025 | OQM0210071 | 400 ng/ml |
| IL-12 | Human | Human | CHO | PeproTech | 200-12B | 0210596, 0707596-2 pooled | 400 ng/ml |
| IL-12 | Mouse | Mouse | CHO | PeproTech | 210-12 | 0407S97 | 400 ng/ml |
| IL-4 | Rhesus | Rhesus  Cyno | E. coli | R&D Systems | 1577-IL-010 | IXV0113111 | 125 ng/ml |
| IL-4 | Human | Human | E. coli | R&D Systems | 204-IL-010 | AG1314021 | 125 ng/ml |
| IL-4 | Mouse | Mouse | E. coli | R&D Systems | 404-ML-010 | BC1613061 | 125 ng/ml |
| IL-2 | Rhesus/Cyno (identical) | Rhesus  Cyno | E. coli | Villinger | N/A | 04/12/2008 | 2 μg/ml |
| IL-2 | Human | Human | E. coli | Peprotech | 200-02 | 101312, 041412 pooled | 2 μg/ml |
| IL-2 | Mouse | Mouse | E. coli | Peprotech | 212-12 | 0608108 | 2 μg/ml |
| *Gamma-inactivated vegetative *Bacillus anthracis* Ames | N/A | Human  Rhesus  Cyno | N/A | BEI Resources/CRP |  | AGD0001331 | 400,000 CFU/ml |
| *Zaïre Ebolavirus-like particles | N/A | Human  Rhesus  Cyno | 293T cells | (in house) | N/A | N/A | Varied |
